# Supplementary material for: Spotting what’s important: Priority areas, connectivity, and conservation of the Northern Tiger Cat (Leopardus tigrinus) in Colombia
Source: PLoS One. 2022 Sep 13;17(9):e0273750. doi: 10.1371/journal.pone.0273750 (PMC9469974; doi:10.1371/journal.pone.0273750)

***Spot*ting what´s important: priority areas, connectivity, and conservation of the Northern Tiger Cat (*Leopardus tigrinus*) in Colombia**

José F. González-Maya, Diego A. Zárrate-Charry, Andrés Arias-Alzate, Leonardo Lemus-Mejía, Angela P. Hurtado-Moreno, Magda Gissella Vargas-Gómez, Teresa Andrea Cárdenas, Victor Mallarino, Jan Schipper

**SUPPORTING INFORMATION**

**S2 Fig**

**Supporting Information 2 (S2 Figure).** Map of ecoregions selected for the species distribution modeling of *Leopardus tigrinus* in Colombia. Background shows topography [79]; map created using ArcGIS [75].


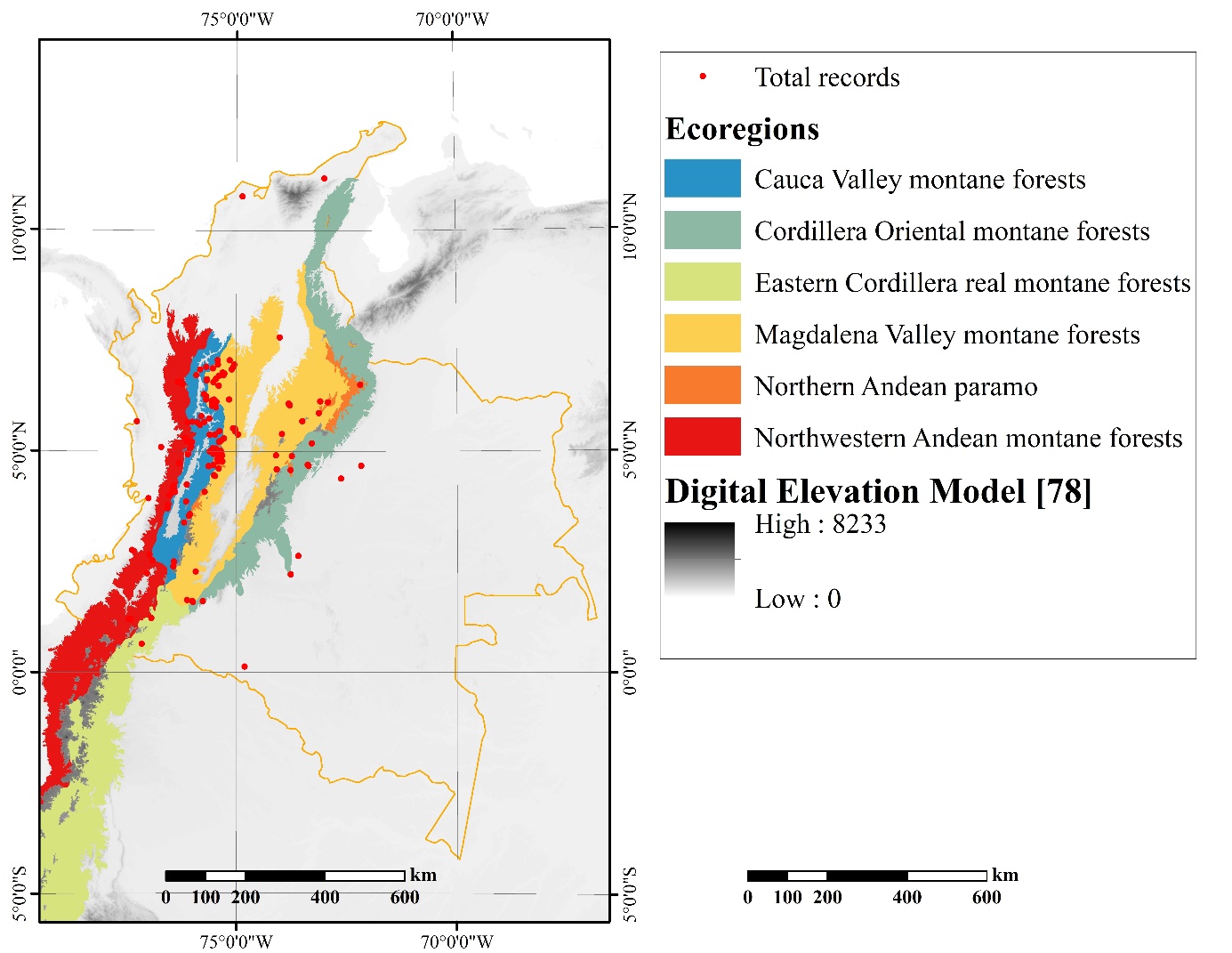

Supplement: S1 Fig — Background shows topography [79]; map created using ArcGIS [75]. (DOCX) [file pone.0273750.s001.docx]
