# Supplementary material for: Spotting what’s important: Priority areas, connectivity, and conservation of the Northern Tiger Cat (Leopardus tigrinus) in Colombia
Source: PLoS One. 2022 Sep 13;17(9):e0273750. doi: 10.1371/journal.pone.0273750 (PMC9469974; doi:10.1371/journal.pone.0273750)
Supplement: S1 Table — (DOCX) [file pone.0273750.s003.docx]

***Spot*ting what´s important: priority areas, connectivity, and conservation of the Northern Tiger Cat (*Leopardus tigrinus*) in Colombia**

José F. González-Maya, Diego A. Zárrate-Charry, Andrés Arias-Alzate, Leonardo Lemus-Mejía, Angela P. Hurtado-Moreno, Magda Gissella Vargas-Gómez, Teresa Andrea Cárdenas, Victor Mallarino, Jan Schipper

**SUPPORTING INFORMATION**

**S1 TABLE**

**Supporting Information 1 (S1 Table).** Records used for species distribution modeling of *Leopardus tigrinus* in Colombia classified according to filters for type of evidence, source, and geographic precision.

| **Source** | **Year** | **Long** | **Lat** | **Department** | **Locality** | **Municipality** | **Evidence** | **Evidence**  **category** | **Source**  **category** | **Geographic**  **category** |
| --- | --- | --- | --- | --- | --- | --- | --- | --- | --- | --- |
| Museum | 1959 | -77.01000 | 2.24000 | Cauca | - | - | Preserved Specimen | High | Medium | Medium |
| Tecnical info | 2010 | -76.98050 | 3.94243 | Valle Del Cauca | Chicoral | La Cumbre | Human Observation | Low | Low | Medium |
| Museum | 1958 | -74.08333 | 4.60000 | Cundinamarca | - | Bogota | Preserved Specimen | High | Medium | High |
| Museum | 1958 | -74.08333 | 4.60000 | Cundinamarca | - | Bogota | Preserved Specimen | High | Medium | High |
| Museum | 1958 | -74.08333 | 4.60000 | Cundinamarca | - | Bogota | Preserved Specimen | High | Medium | High |
| Museum | 1958 | -76.95000 | 2.66667 | Cauca | Charguayaco | El Tambo | Preserved Specimen | High | Medium | High |
| Museum | 1955 | -76.88333 | 2.53333 | Cauca | Sabanetas | El Tambo | Preserved Specimen | High | Medium | High |
| Museum | 1955 | -76.41667 | 2.40000 | Cauca | La Quintana | Totoro | Preserved Specimen | High | Medium | Medium |
| Museum | 1958 | -76.40000 | 2.50000 | Cauca | - | Totoro | Preserved Specimen | High | Medium | High |
| Tecnical info | 2010 | -76.29667 | 4.74167 | Valle Del Cauca | Cerro el Ingles | El Cairo | Human Observation | High | Low | High |
| Tecnical info | 2010 | -76.29667 | 4.74167 | Valle Del Cauca | Cerro el Ingles | El Cairo | Material Sample | Medium | Low | High |
| Tecnical info | 2010 | -76.29667 | 4.74167 | Valle Del Cauca | Cerro el Ingles | El Cairo | Human Observation | Low | Low | High |
| Museum | 1951 | -75.98333 | 1.61667 | Huila | San Adolfo | Acevedo | Preserved Specimen | High | Medium | Low |
| Peer-reviewed article | 1992 | -75.60000 | 5.00000 | Caldas | Reserva Natural Planalto | Manizales | Preserved Specimen | High | High | Medium |
| Peer-reviewed article | 1992 | -75.48333 | 5.38333 | Caldas | Area Urbana | Manizales | Preserved Specimen | High | High | Medium |
| Museum | 1997 | -73.09861 | 6.13806 | Santander | - | Encino | Preserved Specimen | High | Medium | High |
| Museum | 2018 | -75.44942 | 6.12932 | Antioquia | - | Rionegro | Preserved Specimen | High | Medium | High |
| Museum | 2013 | -73.12847 | 5.86881 | Boyaca | - | Paipa | Preserved Specimen | High | Medium | High |
| Museum | 2013 | -76.54139 | 3.78500 | Valle Del Cauca | Finca las Acacias, Vereda El Aguacate | Restrepo | Preserved Specimen | High | Medium | High |
| GBIF | 2016 | -73.81077 | 6.08862 | Santander | Vereda San Pablo | El Peñon | Machine Observation | High | Medium | High |
| GBIF | 2016 | -73.81077 | 6.08862 | Santander | Vereda San Pablo | El Peñon | Machine Observation | High | Medium | High |
| GBIF | 2016 | -73.79722 | 6.05639 | Santander | Vereda Ojo de Agua | El Peñon | Machine Observation | High | Medium | High |
| GBIF | 2016 | -73.79722 | 6.05639 | Santander | Vereda Ojo de Agua | El Peñon | Machine Observation | High | Medium | High |
| GBIF | 2016 | -73.79722 | 6.05639 | Santander | Vereda Ojo de Agua | El Peñon | Machine Observation | High | Medium | High |
| GBIF | 2016 | -73.79722 | 6.05639 | Santander | Vereda Ojo de Agua | El Peñon | Machine Observation | High | Medium | High |
| GBIF | 2016 | -73.79722 | 6.05639 | Santander | Vereda Ojo de Agua | El Peñon | Machine Observation | High | Medium | High |
| GBIF | 2016 | -73.79722 | 6.05639 | Santander | Vereda Ojo de Agua | El Peñon | Machine Observation | High | Medium | High |
| GBIF | 2016 | -73.79722 | 6.05639 | Santander | Vereda Ojo de Agua | El Peñon | Machine Observation | High | Medium | High |
| GBIF | 2016 | -73.79722 | 6.05639 | Santander | Vereda Ojo de Agua | El Peñon | Machine Observation | High | Medium | High |
| GBIF | 2016 | -73.79722 | 6.05639 | Santander | Vereda Ojo de Agua | El Peñon | Machine Observation | High | Medium | High |
| GBIF | 2016 | -73.79722 | 6.05639 | Santander | Vereda Ojo de Agua | El Peñon | Machine Observation | High | Medium | High |
| GBIF | 2016 | -73.79722 | 6.05639 | Santander | Vereda Ojo de Agua | El Peñon | Machine Observation | High | Medium | High |
| GBIF | 2016 | -73.79722 | 6.05639 | Santander | Vereda Ojo de Agua | El Peñon | Machine Observation | High | Medium | High |
| GBIF | 2016 | -73.79722 | 6.05639 | Santander | Vereda Ojo de Agua | El Peñon | Machine Observation | High | Medium | High |
| GBIF | 2016 | -73.79722 | 6.05639 | Santander | Vereda Ojo de Agua | El Peñon | Machine Observation | High | Medium | High |
| GBIF | 2016 | -73.79722 | 6.05639 | Santander | Vereda Ojo de Agua | El Peñon | Machine Observation | High | Medium | High |
| GBIF | 2016 | -73.79722 | 6.05639 | Santander | Vereda Ojo de Agua | El Peñon | Machine Observation | High | Medium | High |
| GBIF | 2016 | -73.79722 | 6.05639 | Santander | Vereda Ojo de Agua | El Peñon | Machine Observation | High | Medium | High |
| GBIF | 2016 | -73.79722 | 6.05639 | Santander | Vereda Ojo de Agua | El Peñon | Machine Observation | High | Medium | High |
| GBIF | 2016 | -73.79722 | 6.05639 | Santander | Vereda Ojo de Agua | El Peñon | Machine Observation | High | Medium | High |
| GBIF | 2016 | -73.79722 | 6.05639 | Santander | Vereda Ojo de Agua | El Peñon | Machine Observation | High | Medium | High |
| GBIF | 2016 | -73.79722 | 6.05639 | Santander | Vereda Ojo de Agua | El Peñon | Machine Observation | High | Medium | High |
| GBIF | 2016 | -73.79722 | 6.05639 | Santander | Vereda Ojo de Agua | El Peñon | Machine Observation | High | Medium | High |
| GBIF | 2016 | -73.79722 | 6.05639 | Santander | Vereda Ojo de Agua | El Peñon | Machine Observation | High | Medium | High |
| GBIF | 2016 | -73.79722 | 6.05639 | Santander | Vereda Ojo de Agua | El Peñon | Machine Observation | High | Medium | High |
| GBIF | 2016 | -73.79722 | 6.05639 | Santander | Vereda Ojo de Agua | El Peñon | Machine Observation | High | Medium | High |
| GBIF | 2016 | -73.79722 | 6.05639 | Santander | Vereda Ojo de Agua | El Peñon | Machine Observation | High | Medium | High |
| GBIF | 2016 | -73.79722 | 6.05639 | Santander | Vereda Ojo de Agua | El Peñon | Machine Observation | High | Medium | High |
| GBIF | 2016 | -73.80056 | 6.06972 | Santander | Vereda Ojo de Agua | El Peñon | Machine Observation | High | Medium | High |
| GBIF | 2016 | -73.80056 | 6.06972 | Santander | Vereda Ojo de Agua | El Peñon | Machine Observation | High | Medium | High |
| GBIF | 2016 | -73.80056 | 6.06972 | Santander | Vereda Ojo de Agua | El Peñon | Machine Observation | High | Medium | High |
| GBIF | 2016 | -73.80056 | 6.06972 | Santander | Vereda Ojo de Agua | El Peñon | Machine Observation | High | Medium | High |
| GBIF | 2016 | -73.80056 | 6.06972 | Santander | Vereda Ojo de Agua | El Peñon | Machine Observation | High | Medium | High |
| GBIF | 2016 | -73.79722 | 6.05639 | Santander | Vereda Ojo de Agua | El Peñon | Machine Observation | High | Medium | High |
| GBIF | 2016 | -73.79722 | 6.05639 | Santander | Vereda Ojo de Agua | El Peñon | Machine Observation | High | Medium | High |
| GBIF | 2016 | -73.79722 | 6.05639 | Santander | Vereda Ojo de Agua | El Peñon | Machine Observation | High | Medium | High |
| GBIF | 2016 | -73.79722 | 6.05639 | Santander | Vereda Ojo de Agua | El Peñon | Machine Observation | High | Medium | High |
| GBIF | 2016 | -73.79722 | 6.05639 | Santander | Vereda Ojo de Agua | El Peñon | Machine Observation | High | Medium | High |
| GBIF | 2016 | -73.79722 | 6.05639 | Santander | Vereda Ojo de Agua | El Peñon | Machine Observation | High | Medium | High |
| GBIF | 2016 | -73.79722 | 6.05639 | Santander | Vereda Ojo de Agua | El Peñon | Machine Observation | High | Medium | High |
| GBIF | 2016 | -73.79722 | 6.05639 | Santander | Vereda Ojo de Agua | El Peñon | Machine Observation | High | Medium | High |
| GBIF | 2016 | -73.79722 | 6.05639 | Santander | Vereda Ojo de Agua | El Peñon | Machine Observation | High | Medium | High |
| GBIF | 2016 | -73.79250 | 6.04972 | Santander | Vereda Alto Gaital | El Peñon | Machine Observation | High | Medium | High |
| GBIF | 2016 | -73.79250 | 6.04972 | Santander | Vereda Alto Gaital | El Peñon | Machine Observation | High | Medium | High |
| GBIF | 2016 | -73.79250 | 6.04972 | Santander | Vereda Alto Gaital | El Peñon | Machine Observation | High | Medium | High |
| GBIF | 2016 | -73.79250 | 6.04972 | Santander | Vereda Alto Gaital | El Peñon | Machine Observation | High | Medium | High |
| GBIF | 2016 | -73.79250 | 6.04972 | Santander | Vereda Alto Gaital | El Peñon | Machine Observation | High | Medium | High |
| GBIF | 2016 | -73.79250 | 6.04972 | Santander | Vereda Alto Gaital | El Peñon | Machine Observation | High | Medium | High |
| Peer-reviewed article | 1992 | -75.35019 | 4.93350 | Caldas | PNN Los Nevados | Villamaria | No Data | Low | High | High |
| Peer-reviewed article | 2009 | -75.41644 | 4.83136 | Caldas | PNN Los Nevados | Villamaria | Preserved Specimen | High | High | High |
| Peer-reviewed article | 2009 | -75.35138 | 4.94889 | Caldas | PNN Los Nevados; Sector Las Brisas | Villamaria | Preserved Specimen | High | High | Low |
| Peer-reviewed article | 1994 | -75.37739 | 5.02278 | Caldas | Reserva Forestral Protectora Torre IV | Manizales | Preserved Specimen | High | High | Medium |
| Peer-reviewed article | 2003 | -75.41729 | 5.02093 | Caldas | Reserva de la CHEC | Manizales | Preserved Specimen | High | High | Medium |
| Peer-reviewed article | 2012 | -75.46580 | 5.03402 | Caldas | Puente de La Libertad | Manizales | No Data | Low | High | High |
| Peer-reviewed article | 1992 | -75.48333 | 5.38333 | Caldas | Area urbana | Manizales | Preserved Specimen | High | High | Medium |
| Peer-reviewed article | 2013 | -75.41729 | 5.02093 | Caldas | Reserva Forestal Protectora Torre IV | Manizales | No Data | Low | High | Medium |
| Peer-reviewed article | 2003 | -75.38333 | 5.21667 | Caldas | Finca La Estrella, cuchilla El Guayabo, vereda La Cristalina | Neira | Preserved Specimen | High | High | High |
| Peer-reviewed article | 2010 | -75.34791 | 5.24294 | Caldas | - | Marulanda | Machine Observation | High | High | High |
| Peer-reviewed article | 2009 | -75.28028 | 5.29972 | Caldas | Finca el Vergel, vereda El Paramo | Marulanda | Preserved Specimen | High | High | High |
| Peer-reviewed article | 1992 | -75.38243 | 5.46462 | Caldas | - | Pacora | No Data | Low | High | High |
| Peer-reviewed article | 2001 | -75.04008 | 5.52837 | Caldas | vereda La Cabana, corregimiento de Florencia | Samana | Preserved Specimen | High | High | High |
| Peer-reviewed article | 1999 | -75.53333 | 5.07250 | Caldas | Reserva Rioblanco | Manizales | Preserved Specimen | High | High | High |
| Peer-reviewed article | 1999 | -75.53333 | 5.07250 | Caldas | Reserva Rioblanco | Manizales | Human Observation | Low | High | High |
| Peer-reviewed article | 1999 | -75.53333 | 5.07250 | Caldas | Reserva Rioblanco | Manizales | Material Sample | Medium | High | High |
| GBIF | 1992 | -75.82000 | 5.60000 | Antioquia | - | Jardin | Human Observation | Low | Medium | High |
| GBIF | 1992 | -75.68000 | 6.92000 | Antioquia | - | San Andres De Cuerquia | Human Observation | Low | Medium | High |
| GBIF | 1992 | -75.29000 | 6.73000 | Antioquia | - | Carolina Del Principe | Human Observation | Low | Medium | High |
| GBIF | 1992 | -75.79000 | 5.80000 | Antioquia | - | Jerico | Human Observation | Low | Medium | High |
| GBIF | 1992 | -75.61000 | 5.75000 | Antioquia | - | La Pintada | Human Observation | Low | Medium | High |
| GBIF | 1992 | -75.82000 | 6.86000 | Antioquia | - | Sabanalarga | Human Observation | Low | Medium | High |
| GBIF | 1992 | -75.42000 | 6.97000 | Antioquia | - | Yarumal | Human Observation | Low | Medium | High |
| GBIF | 1992 | -75.46000 | 6.65000 | Antioquia | - | Santa Rosa De Osos | Human Observation | Low | Medium | High |
| GBIF | 1992 | -75.40000 | 6.49000 | Antioquia | - | Don Matias | Human Observation | Low | Medium | High |
| GBIF | 1992 | -75.15000 | 7.07000 | Antioquia | - | Anori | Human Observation | Low | Medium | High |
| Peer-reviewed article | 2007 | -75.62203 | 4.67419 | Risaralda | Parque Natural Regional Bremen – Barbas | Filandia | Human Observation | Low | High | Medium |
| GBIF | 2019 | -76.10119 | 5.66089 | Antioquia | - | Bolivar | Human Observation | Low | Medium | Low |
| GBIF | 2017 | -73.50541 | 5.68722 | Boyaca | - | Arcabuco | Human Observation | Low | Medium | Medium |
| Museum | 1951 | -75.98333 | 1.61667 | Huila | San Adolfo | Acevedo | Preserved Specimen | High | Medium | Low |
| Museum | 1955 | -76.41667 | 2.40000 | Cauca | La Quintana | - | Preserved Specimen | High | Medium | Medium |
| Museum | 1958 | -76.40000 | 2.50000 | Cauca | - | Totoro | Preserved Specimen | High | Medium | High |
| Museum | 1955 | -76.88333 | 2.53333 | Cauca | - | Sabanetas | Preserved Specimen | High | Medium | Medium |
| Museum | 1958 | -76.95000 | 2.66667 | Cauca | Charguayaco | - | Preserved Specimen | High | Medium | Medium |
| Museum | 1952 | -74.08333 | 4.60000 | Cundinamarca | - | Bogota | Preserved Specimen | High | Medium | High |
| Museum | 1952 | -74.08333 | 4.60000 | Cundinamarca | - | Bogota | Preserved Specimen | High | Medium | High |
| Museum | 1952 | -74.08333 | 4.60000 | Cundinamarca | - | Bogota | Preserved Specimen | High | Medium | High |
| GBIF | 1992 | -75.72000 | 5.67000 | Antioquia | - | Támesis | Human Observation | Low | Medium | High |
| GBIF | 1992 | -75.40000 | 6.49000 | Antioquia | - | Don Matías | Human Observation | Low | Medium | High |
| GBIF | 1992 | -75.46000 | 6.65000 | Antioquia | - | Santa Rosa De Osos | Human Observation | Low | Medium | High |
| GBIF | 1992 | -75.68000 | 6.92000 | Antioquia | - | San Andres De Cuerquia | Human Observation | Low | Medium | High |
| Museum | 1997 | -73.09870 | 6.13823 | Santander | Santander: Encino. | Encino | Preserved Specimen | High | Medium | High |
| Museum | 1992 | -75.38389 | 5.22944 | Caldas | Vda La Cristalina, cuchilla El Guayabo, cuenca alta del Rio Tapias | Neira | Preserved Specimen | High | Medium | Medium |
| GBIF | 1992 | -75.67000 | 6.61000 | Antioquia | - | Belmira | Human Observation | Low | Medium | High |
| GBIF | 1992 | -75.91000 | 6.73000 | Antioquia | - | Buriticá | Human Observation | Low | Medium | High |
| GBIF | 1992 | -75.52000 | 6.57000 | Antioquia | - | Entrerrios | Human Observation | Low | Medium | High |
| GBIF | 2013 | -77.34628 | 2.77017 | Cauca | Comunidad indIgenas de ASIESCA | Timbiqui | Human Observation | Low | Medium | High |
| GBIF | 1992 | -75.07000 | 6.91000 | Antioquia | - | Amalfi | Human Observation | Low | Medium | High |
| GBIF | 1992 | -75.42000 | 6.97000 | Antioquia | - | Yarumal | Human Observation | Low | Medium | High |
| GBIF | 1992 | -75.15000 | 7.07000 | Antioquia | - | Anorí | Human Observation | Low | Medium | High |
| GBIF | 1992 | -75.29000 | 6.73000 | Antioquia | - | Carolina Del Principe | Human Observation | Low | Medium | High |
| GBIF | 1992 | -75.82000 | 5.60000 | Antioquia | - | Jardín | Human Observation | Low | Medium | High |
| Museum | 1992 | -75.62000 | 4.67167 | Quindio | Reserva Forestal Bremen, | Circasia | Preserved Specimen | High | Medium | Medium |
| GBIF | 1992 | -75.61000 | 5.75000 | Antioquia | - | La Pintada | Human Observation | Low | Medium | High |
| GBIF | 1992 | -75.79000 | 5.80000 | Antioquia | - | Jericó | Human Observation | Low | Medium | High |
| GBIF | 1992 | -75.82000 | 6.86000 | Antioquia | - | Sabanalarga | Human Observation | Low | Medium | High |
| GBIF | 1992 | -75.88000 | 5.66000 | Antioquia | - | Andes | Human Observation | Low | Medium | High |
| Museum | 1997 | -73.09871 | 6.13823 | Santander | Santander: Encino. | Encino | Preserved Specimen | High | Medium | High |
| Peer-reviewed article | 1992 | -74.10000 | 4.91667 | Cundinamarca | Tabio | Tabio | No Data | Low | High | High |
| Peer-reviewed article | 1992 | -74.95778 | 5.38792 | - | Samana | Samana | No Data | Low | High | Medium |
| Peer-reviewed article | 1992 | -75.37739 | 5.02278 | Caldas | Manizales | Manizales | No Data | Low | High | Medium |
| Peer-reviewed article | 1992 | -75.34788 | 5.24293 | - | Marulanda | Marulanda | No Data | Low | High | Medium |
| Peer-reviewed article | 1992 | -75.38333 | 5.21667 | Caldas | Neira | Neira | No Data | Low | High | High |
| Peer-reviewed article | 1992 | -75.38243 | 5.46462 | Caldas | PAcora | Pacora | No Data | Low | High | High |
| Peer-reviewed article | 1992 | -75.04016 | 5.52690 | - | Samana | Samana | No Data | Low | High | Medium |
| Peer-reviewed article | 1992 | -75.04008 | 5.52837 | Caldas | Villamaria | Villamaria | No Data | Low | High | Medium |
| Peer-reviewed article | 1992 | -75.41645 | 4.83136 | Caldas | Villamaria | Villamaria | No Data | Low | High | High |
| Peer-reviewed article | 1992 | -75.35139 | 4.94889 | Caldas | Villamaria | Villamaria | No Data | Low | High | Low |
| Peer-reviewed article | 1992 | -75.41729 | 5.02093 | Caldas | Villamaria | Villamaria | No Data | Low | High | High |
| Peer-reviewed article | 1992 | -75.46580 | 5.03402 | Caldas | Manizales | Manizales | No Data | Low | High | High |
| Peer-reviewed article | 1992 | -75.48333 | 5.38333 | Caldas | Salaminas | Salamina | No Data | Low | High | High |
| Peer-reviewed article | 1992 | -75.41729 | 5.02093 | Caldas | Salaminas | Salaminas | No Data | Low | High | Medium |
| Peer-reviewed article | 1992 | -75.28028 | 5.29972 | - | Marulanda | Marulanda | No Data | Low | High | Medium |
| Peer-reviewed article | 1992 | -75.97222 | 1.59722 | - | Acevedo | Acevedo | No Data | Low | High | Low |
| Peer-reviewed article | 1992 | -74.80747 | 0.13182 | Putumayo | Puerto leguizamo | Puerto Leguizamo | No Data | Low | High | High |
| Peer-reviewed article | 1992 | -75.48333 | 5.38333 | Caldas | Salaminas | Salamina | No Data | Low | High | High |
| Peer-reviewed article | 1992 | -75.60000 | 5.00000 | Caldas | Manizales | Manizales | No Data | Low | High | Medium |
| Peer-reviewed article | 1992 | -75.38328 | 5.21660 | Caldas | Neira | Neira | No Data | Low | High | High |
| Peer-reviewed article | 1992 | -75.35019 | 4.93350 | Caldas | Villamaria | Villamaria | No Data | Low | High | High |
| Peer-reviewed article | 1992 | -75.35139 | 4.94889 | Caldas | Villamaria | Villamaria | No Data | Low | High | Low |
| Peer-reviewed article | 1992 | -75.48333 | 5.38333 | Caldas | Salaminas | Salamina | No Data | Low | High | High |
| Peer-reviewed article | 1992 | -75.50028 | 5.05722 | Caldas | Manizales | Manizales | No Data | Low | High | High |
| Peer-reviewed article | 1992 | -75.58811 | 4.99839 | Caldas | Manizales | Manizales | No Data | Low | High | High |
| Peer-reviewed article | 1992 | -75.45534 | 5.07042 | Caldas | Manizales | Manizales | No Data | Low | High | High |
| Peer-reviewed article | 1992 | -75.46580 | 5.03402 | Caldas | Manizales | Manizales | No Data | Low | High | High |
| Peer-reviewed article | 1992 | -75.55000 | 6.10000 | - | - | - | No Data | Low | High | Low |
| Expert validated record | 2009 | -75.31140 | 6.78810 | - | - | - | Material Sample | Medium | High | Low |
| Expert validated record | 2009 | -75.53170 | 6.88360 | - | - | - | Human Observation | Low | High | Low |
| Expert validated record | 2011 | -75.27545 | 6.76900 | - | - | - | Machine Observation | High | High | Low |
| Expert validated record | 2006 | -75.66670 | 6.63330 | - | - | - | Human Observation | Low | High | Low |
| Expert validated record | 2012 | -75.54750 | 6.10750 | - | - | - | Machine Observation | High | High | Low |
| Expert validated record | 2012 | -75.16351 | 6.17949 | - | - | - | Machine Observation | High | High | Low |
| Expert validated record | 2009 | -75.31140 | 6.78810 | - | - | - | Machine Observation | High | High | Low |
| Expert validated record | 2009 | -75.53170 | 6.88360 | - | - | - | Material Sample | Medium | High | Low |
| Expert validated record | 2005 | -75.42140 | 7.07280 | - | - | - | Material Sample | Medium | High | Low |
| Expert validated record | 2011 | -75.27545 | 6.76900 | - | - | - | Machine Observation | High | High | Low |
| Expert validated record | 2011 | -75.28805 | 6.74084 | - | - | - | Preserved Specimen | High | High | Low |
| Expert validated record | 2006 | -75.66670 | 6.63330 | - | - | - | Material Sample | Medium | High | Low |
| Expert validated record | 2003 | -75.10420 | 6.85920 | - | - | - | Human Observation | Low | High | Low |
| Expert validated record | 2012 | -75.54750 | 6.10750 | - | - | - | Machine Observation | High | High | Low |
| Expert validated record | 2012 | -75.16351 | 6.17949 | - | - | - | Machine Observation | High | High | Low |
| Expert validated record | 2013 | -75.54845 | 6.12513 | - | - | - | Preserved Specimen | High | High | Low |
| Expert validated record | 2008 | -75.53223 | 6.15384 | - | - | - | Preserved Specimen | High | High | Low |
| Expert validated record | 2005 | -75.54361 | 6.12139 | - | - | - | Preserved Specimen | High | High | Low |
| Expert validated record | 2012 | -75.54806 | 6.12667 | - | - | - | Preserved Specimen | High | High | Low |
| Expert validated record | 2013 | -75.54667 | 6.15611 | - | - | - | Preserved Specimen | High | High | Low |
| Expert validated record | 2008 | -75.53328 | 6.15638 | - | - | - | Preserved Specimen | High | High | Low |
| Expert validated record | 2007 | -75.54722 | 6.12417 | - | - | - | Preserved Specimen | High | High | Low |
| Expert validated record | 2010 | -75.54556 | 6.12333 | - | - | - | Preserved Specimen | High | High | Low |
| Expert validated record | 2011 | -75.54806 | 6.12583 | - | - | - | Preserved Specimen | High | High | Low |
| Expert validated record | 2012 | -75.39640 | 6.71810 | - | - | - | Machine Observation | High | High | Low |
| Expert validated record | 2014 | -75.53936 | 6.08343 | - | - | - | Machine Observation | High | High | Low |
| Expert validated record | 2014 | -75.54460 | 6.03354 | - | - | - | Machine Observation | High | High | Low |
| Expert validated record | 2013 | -75.48416 | 6.07515 | - | - | - | Machine Observation | High | High | Low |
| Expert validated record | 2014 | -75.45913 | 5.99904 | - | - | - | Machine Observation | High | High | Low |
| Expert validated record | 2014 | -75.50557 | 6.17822 | - | - | - | Machine Observation | High | High | Low |
| Expert validated record | 2013 | -75.54845 | 6.12513 | - | - | - | Preserved Specimen | High | High | Low |
| Expert validated record | 2008 | -75.53223 | 6.15384 | - | - | - | Preserved Specimen | High | High | Low |
| Expert validated record | 2005 | -75.54361 | 6.12139 | - | - | - | Preserved Specimen | High | High | Low |
| Expert validated record | 2012 | -75.54806 | 6.12667 | - | - | - | Preserved Specimen | High | High | Low |
| Expert validated record | 2013 | -75.54667 | 6.15611 | - | - | - | Preserved Specimen | High | High | Low |
| Expert validated record | 2007 | -75.54722 | 6.12417 | - | - | - | Preserved Specimen | High | High | Low |
| Expert validated record | 2010 | -75.54556 | 6.12333 | - | - | - | Preserved Specimen | High | High | Low |
| Expert validated record | 2011 | -75.54806 | 6.12583 | - | - | - | Preserved Specimen | High | High | Low |
| Expert validated record | 2009 | -75.55000 | 6.10000 | Antioquia | Reserva San Sebastian- La Castellana | - | Human Observation | Low | High | Medium |
| Expert validated record | 2010 | -72.99100 | 11.19039 | La Guajira | Corralejas | San Juan Del Cesar | Material Sample | Medium | High | Medium |
| Expert validated record | 2003 | -74.86667 | 10.78333 | Atlantico | Sin especificar | Polonuevo | No Data | Low | High | High |
| Expert validated record | 2002 | -75.68222 | 6.17806 | Antioquia | El Chupadero | Don Matias | No Data | Low | High | Medium |
| Expert validated record | 2002 | -75.68222 | 6.25056 | Antioquia | Padre Amaya | Medellin | No Data | Low | High | High |
| Expert validated record | 2002 | -75.73278 | 6.29861 | Antioquia | Miraflores | Ebejico | No Data | Low | High | High |
| Expert validated record | 2011 | -75.28805 | 6.74084 | Antioquia | - | Amalfi | Material Sample | Medium | High | Medium |
| Expert validated record | 2013 | -75.54845 | 6.12513 | Antioquia | Reserva San Sebastian la Castellana | Medellin | Material Sample | Medium | High | Medium |
| Expert validated record | 2008 | -75.53223 | 6.15384 | Antioquia | Via Las Palmas | Envigado | Material Sample | Medium | High | Medium |
| Expert validated record | 2005 | -75.54361 | 6.12139 | Antioquia | carretera El Escobero | Envigado | Material Sample | Medium | High | High |
| Expert validated record | 2012 | -75.54806 | 6.12667 | Antioquia | carretera El Escobero | Envigado | Material Sample | Medium | High | High |
| Expert validated record | 2013 | -75.54667 | 6.15611 | Antioquia | Las Palmas | Envigado | Material Sample | Medium | High | High |
| Expert validated record | 2008 | -75.53328 | 6.15638 | Antioquia | v1a glorieta Las Palmas hacia Santa Elena | Envigado | Material Sample | Medium | High | Medium |
| Expert validated record | 2007 | -75.54722 | 6.12417 | Antioquia | carretera El Escobero | Envigado | Material Sample | Medium | High | High |
| Expert validated record | 2010 | -75.54556 | 6.12333 | Antioquia | carretera El Escobero | Envigado | Material Sample | Medium | High | High |
| Expert validated record | 2011 | -75.54806 | 6.12583 | Antioquia | carretera El Escobero | Santa Rosa De Osos | Material Sample | Medium | High | Medium |
| Expert validated record | 2003 | -75.10420 | 6.85920 | Antioquia | Vereda Guayabito | Retiro | Human Observation | Low | High | Medium |
| Expert validated record | 2009 | -75.31140 | 6.78810 | Antioquia | Vereda Miraflores | Carolina Del Principe | Material Sample | Medium | High | Medium |
| Expert validated record | 2011 | -75.27545 | 6.76900 | Antioquia | Vereda Agua Bonita | Carolina Del Principe | Machine Observation | High | High | Medium |
| Expert validated record | 2012 | -75.54750 | 6.10750 | Antioquia | Reserva San Sebastian la Castellana | Guatape | Machine Observation | High | High | Medium |
| Expert validated record | 2012 | -75.16351 | 6.17949 | Antioquia | Vereda La Sonadora | Retiro | Machine Observation | High | High | Medium |
| Expert validated record | 2012 | -75.39640 | 6.71810 | Antioquia | Vereda Guanacas | Retiro | Machine Observation | High | High | Medium |
| Expert validated record | 2014 | -75.53936 | 6.08343 | Antioquia | Vereda Normand1a Baja | Retiro | Machine Observation | High | High | High |
| Expert validated record | 2014 | -75.54460 | 6.03354 | Antioquia | Vereda El Carmen | Retiro | Machine Observation | High | High | High |
| Expert validated record | 2013 | -75.48416 | 6.07515 | Antioquia | Vereda Ranchería | Retiro | Machine Observation | High | High | High |
| Expert validated record | 2014 | -75.45913 | 5.99904 | Antioquia | Vereda Pantanillo, Cerro Plata | Envigado | Machine Observation | High | High | Medium |
| Expert validated record | 2014 | -75.50557 | 6.17822 | Antioquia | Vereda Pantanillo | Amalfi | Machine Observation | High | High | Medium |
| Expert validated record | 2015 | -75.04464 | 6.97228 | Antioquia | Vereda la Manguita | Maceo | Machine Observation | High | High | Medium |
| Peer-reviewed article | 2017 | -74.01494 | 7.58593 | Cundinamarca | Vereda Verjón Bajo | Bogota | Material Sample | Medium | High | Low |
| Peer-reviewed article | 2016 | -75.39623 | 4.61747 | Caldas | CHINCHINA | Chinchina | Machine Observation | High | High | Low |
| Museum | 2013 | -75.35019 | 5.01680 | Caldas | PNN Los Nevados; Sector Las Brisas | Villamaria | Preserved Specimen | High | Medium | Medium |
| Museum | 2009 | -75.35139 | 4.93350 | Caldas | PNN Los Nevados; Sector Las Brisas | Villamaria | Preserved Specimen | High | Medium | High |
| Museum | 2013 | -75.42667 | 4.94889 | Caldas | reserva Bosques de la CHEC | Villamaria | Preserved Specimen | High | Medium | High |
| Museum | 2013 | -75.48333 | 5.02093 | Caldas | Manizales | Manizales | Preserved Specimen | High | Medium | Medium |
| Peer-reviewed article | 2013 | -75.58811 | 5.38333 | Caldas | Reserva de Plan Alto | Manizales | Preserved Specimen | High | High | Medium |
| Peer-reviewed article | 2013 | -75.45534 | 4.99839 | Caldas | Reserva de Rio Blanco | Manizales | Preserved Specimen | High | High | Medium |
| Peer-reviewed article | 2012 | -75.46580 | 5.07042 | Caldas | Manizales | Manizales | Human Observation | Low | High | High |
| Museum | 2003 | -75.48333 | 5.03402 | Caldas | Manizales | Manizales | Preserved Specimen | High | Medium | Medium |
| Museum | 2003 | -75.60000 | 5.38333 | Caldas | Reserva Natural Planalto | Manizales | Preserved Specimen | High | Medium | Medium |
| Museum | 2003 | -75.38333 | 5.00000 | Caldas | Vereda la Cristalina | Neira | Preserved Specimen | High | Medium | Medium |
| GBIF | 2019 | -75.99483 | 5.21667 | Antioquia | - | - | Human Observation | Low | Medium | Low |
| GBIF | 2019 | -76.10119 | 5.66089 | Antioquia | - | - | Human Observation | Low | Medium | Low |
| GBIF | 2017 | -73.50541 | 5.68722 | Boyaca | - | - | Human Observation | Low | Medium | Medium |
| Museum | 2009 | -75.41644 | 4.83136 | Caldas | PNN Los Nevados; Sector Las Brisas | Villamaria | Preserved Specimen | High | Medium | High |
| Museum | 1994 | -75.37739 | 5.02278 | Caldas | - | Manizales | Human Observation | Low | Medium | Medium |
| Museum | 2003 | -75.41729 | 5.02093 | Caldas | reserva Bosques de la CHEC | Manizales | Preserved Specimen | High | Medium | Medium |
| Museum | 2013 | -75.41729 | 5.02093 | Caldas | Reserva Forestal Protectora Torre 4 | Manizales | Machine Observation | High | Medium | Medium |
| Museum | 2003 | -75.38333 | 5.21667 | Caldas | vereda La Cristalina | Neira | Preserved Specimen | High | Medium | High |
| Museum | 1992 | -75.48333 | 5.38333 | Caldas | Manizales | Manizales | Human Observation | Low | Medium | Medium |
| Peer-reviewed article | 2010 | -75.34791 | 5.24294 | Caldas | - | Marulanda | Preserved Specimen | High | High | High |
| Museum | 2009 | -75.28028 | 5.29972 | Caldas | vereda El Páramo | Marulanda | Preserved Specimen | High | Medium | High |
| Peer-reviewed article | 1992 | -75.38243 | 5.46462 | Caldas | - | Pacora | Preserved Specimen | High | High | High |
| Museum | 2001 | -75.04008 | 5.52837 | Caldas | vereda La Cabaña | Samana | Preserved Specimen | High | Medium | High |
| GBIF | 2019 | -77.12546 | 0.64538 | Putumayo | Venado | - | Machine Observation | High | Medium | Medium |
| GBIF | 2019 | -75.99483 | 5.67440 | Antioquia | - | - | Human Observation | Low | Medium | Medium |
| GBIF | 2017 | -73.73990 | 4.89950 | Cundinamarca | Monquetiva | - | Machine Observation | High | Medium | Medium |
| GBIF | 2018 | -75.59574 | 4.99916 | Caldas | Cenicafe, Reserva Planalto | - | Preserved Specimen | High | Medium | Medium |
| GBIF | 2009 | -75.35434 | 4.95027 | Caldas | Parque Nacional Natural Los Nevados | - | Preserved Specimen | High | Medium | Medium |
| GBIF | 1992 | -75.47489 | 5.04836 | Caldas | Zona urbana | - | Preserved Specimen | High | Medium | Medium |
| GBIF | 2009 | -75.41226 | 4.77959 | Risaralda | Parque Nacional Natural Los Nevados | - | Preserved Specimen | High | Medium | Medium |
| GBIF | 1992 | -75.37739 | 5.02278 | Caldas | Torre 4 | - | Preserved Specimen | High | Medium | Medium |
| GBIF | 2009 | -75.41644 | 4.83136 | Caldas | Parque Nacional Natural Los Nevados | - | Preserved Specimen | High | Medium | Medium |
| GBIF | 1985 | -75.51571 | 4.94410 | Caldas | Valles | - | Preserved Specimen | High | Medium | Medium |
| GBIF | 2002 | -75.48167 | 5.06583 | Caldas | Ecoparque Los Yarumos | - | Material Sample | Medium | Medium | Medium |
| GBIF | 2015 | -75.33766 | 4.98321 | - | Parque Nacional Natural Los Nevados | Los Nevados | Machine Observation | High | Medium | Low |
| GBIF | 2015 | -75.33766 | 4.98321 | - | Parque Nacional Natural Los Nevados | Los Nevados | Machine Observation | High | Medium | Low |
| GBIF | 2015 | -75.33766 | 4.98321 | - | Parque Nacional Natural Los Nevados | Los Nevados | Machine Observation | High | Medium | Low |
| GBIF | 2016 | -76.08600 | 4.94742 | - | Parque Nacional Natural Tatama | Tatama | Machine Observation | High | Medium | Low |
| GBIF | 2017 | -76.22141 | 6.52234 | - | Parque Nacional Natural Las Orquideas | Las Orquideas | Machine Observation | High | Medium | Low |
| GBIF | 2017 | -77.41068 | 1.20963 | - | Santuario de Flora y Fauna Galeras | Galeras | Machine Observation | High | Medium | Low |
| GBIF | 2017 | -76.32541 | 6.57661 | - | Parque Nacional Natural Las Orquideas | Las Orquideas | Machine Observation | High | Medium | Low |
| GBIF | 2017 | -77.41068 | 1.20963 | - | Santuario de Flora y Fauna Galeras | Galeras | Machine Observation | High | Medium | Low |
| GBIF | 2015 | -75.33766 | 4.98321 | - | Parque Nacional Natural Los Nevados | Los Nevados | Machine Observation | High | Medium | Low |
| GBIF | 2017 | -77.40492 | 1.21095 | - | Santuario de Flora y Fauna Galeras | Galeras | Machine Observation | High | Medium | Low |
| GBIF | 2016 | -76.07475 | 5.21550 | - | Parque Nacional Natural Tatama | Tatama | Machine Observation | High | Medium | Low |
| GBIF | 2016 | -76.16319 | 5.29981 | - | Parque Nacional Natural Tatama | Tatama | Machine Observation | High | Medium | Low |
| GBIF | 2015 | -75.33766 | 4.98321 | - | Parque Nacional Natural Los Nevados | Los Nevados | Machine Observation | High | Medium | Low |
| GBIF | 2015 | -75.33766 | 4.98321 | - | Parque Nacional Natural Los Nevados | Los Nevados | Machine Observation | High | Medium | Low |
| GBIF | 2016 | -76.10103 | 5.22925 | - | Parque Nacional Natural Tatama | Tatama | Machine Observation | High | Medium | Low |
| GBIF | 2017 | -76.22141 | 6.52234 | - | Parque Nacional Natural Las Orquideas | Las Orquideas | Machine Observation | High | Medium | Low |
| GBIF | 2017 | -77.40492 | 1.21095 | - | Santuario de Flora y Fauna Galeras | Galeras | Machine Observation | High | Medium | Low |
| GBIF | 2017 | -76.22247 | 6.54946 | - | Parque Nacional Natural Las Orquideas | Las Orquideas | Machine Observation | High | Medium | Low |
| GBIF | 2016 | -76.01369 | 5.11181 | - | Parque Nacional Natural Tatama | Tatama | Machine Observation | High | Medium | Low |
| GBIF | 2017 | -76.32541 | 6.57661 | - | Parque Nacional Natural Las Orquideas | Las Orquideas | Machine Observation | High | Medium | Low |
| GBIF | 2016 | -76.07869 | 4.97283 | - | Parque Nacional Natural Tatama | Tatama | Machine Observation | High | Medium | Low |
| GBIF | 2016 | -76.11319 | 5.26453 | - | Parque Nacional Natural Tatama | Tatama | Machine Observation | High | Medium | Low |
| GBIF | 2017 | -76.32541 | 6.57661 | - | Parque Nacional Natural Las Orquideas | Las Orquideas | Machine Observation | High | Medium | Low |
| GBIF | 2016 | -76.10103 | 5.22925 | - | Parque Nacional Natural Tatama | Tatama | Machine Observation | High | Medium | Low |
| GBIF | 2016 | -76.07475 | 5.21550 | - | Parque Nacional Natural Tatama | Tatama | Machine Observation | High | Medium | Low |
| GBIF | 2016 | -76.11319 | 5.26453 | - | Parque Nacional Natural Tatama | Tatama | Machine Observation | High | Medium | Low |
| GBIF | 2016 | -76.10103 | 5.22925 | - | Parque Nacional Natural Tatama | Tatama | Machine Observation | High | Medium | Low |
| GBIF | 2016 | -76.03281 | 5.09158 | - | Parque Nacional Natural Tatama | Tatama | Machine Observation | High | Medium | Low |
| GBIF | 2016 | -76.03281 | 5.09158 | - | Parque Nacional Natural Tatama | Tatama | Machine Observation | High | Medium | Low |
| GBIF | 2016 | -76.11319 | 5.26453 | - | Parque Nacional Natural Tatama | Tatama | Machine Observation | High | Medium | Low |
| GBIF | 2017 | -77.41261 | 1.20820 | - | Santuario de Flora y Fauna Galeras | Galeras | Machine Observation | High | Medium | Low |
| GBIF | 2016 | -76.10103 | 5.22925 | - | Parque Nacional Natural Tatama | Tatama | Machine Observation | High | Medium | Low |
| GBIF | 2016 | -76.10103 | 5.22925 | - | Parque Nacional Natural Tatama | Tatama | Machine Observation | High | Medium | Low |
| GBIF | 2017 | -75.07039 | 5.52900 | - | Parque Nacional Natural Selva de Florencia | Selva De Florencia | Machine Observation | High | Medium | Low |
| GBIF | 2016 | -76.10103 | 5.22925 | - | Parque Nacional Natural Tatama | Tatama | Machine Observation | High | Medium | Low |
| GBIF | 2017 | -76.26387 | 6.58888 | - | Parque Nacional Natural Las Orquideas | Las Orquideas | Machine Observation | High | Medium | Low |
| GBIF | 2016 | -76.04628 | 5.19344 | - | Parque Nacional Natural Tatama | Tatama | Machine Observation | High | Medium | Low |
| GBIF | 2016 | -76.09139 | 4.93750 | - | Parque Nacional Natural Tatama | Tatama | Machine Observation | High | Medium | Low |
| GBIF | 2016 | -76.04628 | 5.19344 | - | Parque Nacional Natural Tatama | Tatama | Machine Observation | High | Medium | Low |
| GBIF | 2017 | -75.02089 | 5.45531 | - | Parque Nacional Natural Selva de Florencia | Selva De Florencia | Machine Observation | High | Medium | Low |
| GBIF | 2017 | -77.41531 | 1.20831 | - | Santuario de Flora y Fauna Galeras | Galeras | Machine Observation | High | Medium | Low |
| GBIF | 2016 | -76.04536 | 5.06508 | - | Parque Nacional Natural Tatama | Tatama | Machine Observation | High | Medium | Low |
| GBIF | 2016 | -76.09139 | 4.93750 | - | Parque Nacional Natural Tatama | Tatama | Machine Observation | High | Medium | Low |
| GBIF | 2017 | -77.40575 | 1.20578 | - | Santuario de Flora y Fauna Galeras | Galeras | Machine Observation | High | Medium | Low |
| GBIF | 2017 | -76.10147 | 1.63889 | Huila | Parque Nacional Natural Cueva de los Guacharos | - | Machine Observation | High | Medium | Medium |
| GBIF | 2019 | -76.10119 | 5.66089 | Antioquia | - | - | Human Observation | Low | Medium | Low |
| GBIF | 2015 | -76.47900 | 4.02402 | Valle Del Cauca | Cristalina Alta, Reserva Natural de la Sociedad Civil Buenavista | Darien | Human Observation | Low | Medium | High |
| GBIF | 2015 | -76.41657 | 4.20148 | Valle Del Cauca | La Devora, Reserva Natural de la Sociedad Civil La Cascada | Trujillo | Human Observation | Low | Medium | High |
| GBIF | 2015 | -76.43546 | 4.12136 | Valle Del Cauca | Cristalina Alta, Reserva Natural de la Sociedad Civil La Esmeralda | Riofrio | Human Observation | Low | Medium | High |
| GBIF | 2015 | -76.39826 | 4.17328 | Valle Del Cauca | La Arauca, Reserva Natural de la Sociedad Civil El Silencio | Trujillo | Human Observation | Low | Medium | High |
| GBIF | 2015 | -76.49820 | 4.01280 | Valle Del Cauca | Cristalina Alta, Reserva Natural de la Sociedad Civil La Palmera | Darien | Human Observation | Low | Medium | High |
| GBIF | 2015 | -76.42250 | 4.13093 | Valle Del Cauca | Cristalina Alta, Reserva Natural de la Sociedad Civil Las Palmas | Riofrio | Human Observation | Low | Medium | High |
| GBIF | 2015 | -76.41473 | 4.19576 | Valle Del Cauca | La Devora, Reserva Natural de la Sociedad Civil La Rosa | Trujillo | Human Observation | Low | Medium | High |
| GBIF | 2015 | -76.39789 | 4.23017 | Valle Del Cauca | La Sonora, Reserva Natural de la Sociedad Civil Villa Stella | Trujillo | Human Observation | Low | Medium | High |
| GBIF | 2015 | -76.42360 | 4.20356 | Valle Del Cauca | La Devora, Reserva Natural de la Sociedad Civil Las Nieves | Trujillo | Human Observation | Low | Medium | High |
| GBIF | 2015 | -76.38278 | 4.08625 | Valle Del Cauca | San Pablo, Reserva Natural de la Sociedad Civil Los Naranjos | Riofrio | Human Observation | Low | Medium | High |
| GBIF | 2015 | -76.39735 | 4.08423 | Valle Del Cauca | La Italia, Reserva Natural de la Sociedad Civil La Esperancita | Riofrio | Human Observation | Low | Medium | High |
| GBIF | 2015 | -76.38226 | 4.08799 | Valle Del Cauca | San Pablo, Reserva Natural de la Sociedad Civil Los Guaduales | Riofrio | Human Observation | Low | Medium | High |
| GBIF | 2017 | -73.50541 | 5.68722 | Boyaca | - | - | Human Observation | Low | Medium | Medium |
| GBIF | 2013 | -77.34628 | 2.77017 | Cauca | Comunidad indigenas de ASIESCA | - | Human Observation | Low | Medium | Medium |
| GBIF | 2015 | -75.49536 | 4.46903 | Tolima | Vereda La Luisa, predios de Anglogold Ashanti, transecto El Filo | - | Human Observation | Low | Medium | Medium |
| GBIF | 2014 | -75.49536 | 4.46903 | Tolima | Vereda La Luisa, predios de Anglogold Ashanti, transecto El Filo | - | Human Observation | Low | Medium | Medium |
| GBIF | 2014 | -75.48053 | 4.45175 | Tolima | Vereda La Luisa, transecto quebrada La Colosa | - | Human Observation | Low | Medium | Medium |
| GBIF | 2014 | -75.48053 | 4.45175 | Tolima | Vereda La Luisa, transecto quebrada La Colosa | - | Human Observation | Low | Medium | Medium |
| GBIF | 2014 | -75.48053 | 4.45175 | Tolima | Vereda La Luisa, transecto quebrada La Colosa | - | Human Observation | Low | Medium | Medium |
| GBIF | 2014 | -75.49536 | 4.46903 | Tolima | Vereda La Luisa, predios de Anglogold Ashanti, transecto El Filo | - | Human Observation | Low | Medium | Medium |
| GBIF | 2014 | -75.48053 | 4.45175 | Tolima | Vereda La Luisa, transecto quebrada La Colosa | - | Human Observation | Low | Medium | Medium |
| GBIF | 2014 | -75.49536 | 4.46903 | Tolima | Vereda La Luisa, predios de Anglogold Ashanti, transecto El Filo | - | Human Observation | Low | Medium | Medium |
| GBIF | 2014 | -75.48053 | 4.45175 | Tolima | Vereda La Luisa, transecto quebrada La Colosa | - | Human Observation | Low | Medium | Medium |
| GBIF | 2014 | -75.48053 | 4.45175 | Tolima | Vereda La Luisa, transecto quebrada La Colosa | - | Human Observation | Low | Medium | Medium |
| GBIF | 2012 | -75.49536 | 4.46903 | Tolima | Vereda La Luisa, predios de Anglogold Ashanti, transecto El Filo | - | Human Observation | Low | Medium | Medium |
| GBIF | 2014 | -75.49536 | 4.46903 | Tolima | Vereda La Luisa, predios de Anglogold Ashanti, transecto El Filo | - | Human Observation | Low | Medium | Medium |
| GBIF | 2014 | -75.49536 | 4.46903 | Tolima | Vereda La Luisa, predios de Anglogold Ashanti, transecto El Filo | - | Human Observation | Low | Medium | Medium |
| GBIF | 2014 | -75.48053 | 4.45175 | Tolima | Vereda La Luisa, transecto quebrada La Colosa | - | Human Observation | Low | Medium | Medium |
| GBIF | 2013 | -75.48053 | 4.45175 | Tolima | Vereda La Luisa, transecto quebrada La Colosa | - | Human Observation | Low | Medium | Medium |
| GBIF | 2013 | -75.48053 | 4.45175 | Tolima | Vereda La Luisa, transecto quebrada La Colosa | - | Human Observation | Low | Medium | Medium |
| GBIF | 2013 | -75.50128 | 4.46100 | Tolima | Vereda La Paloma, transecto quebrada La Arenosa | - | Human Observation | Low | Medium | Medium |
| GBIF | 2013 | -75.49536 | 4.46903 | Tolima | Vereda La Luisa, predios de Anglogold Ashanti, transecto El Filo | - | Human Observation | Low | Medium | Medium |
| GBIF | 2017 | -75.71028 | 4.09019 | Tolima | "Vereda Yerbabuena, Reserva Loros Andinos" | - | Human Observation | Low | Medium | Medium |
| GBIF | 1992 | -75.38889 | 5.22944 | Caldas | Vereda La Cristalina, cuchilla El Guayabo, cuenca alta del rio Tapias | - | Preserved Specimen | High | Medium | Medium |
| GBIF | 1992 | -75.62000 | 4.67167 | Quindio | Reserva Forestal Bremen | - | Preserved Specimen | High | Medium | Medium |
| GBIF | 2014 | -73.38045 | 4.70423 | Cundinamarca | Vereda Algodones | - | Machine Observation | High | Medium | Medium |
| GBIF | 2016 | -73.36516 | 4.68115 | Cundinamarca | Cerros orientales | - | Machine Observation | High | Medium | Medium |
| GBIF | 2016 | -73.36516 | 4.68115 | Cundinamarca | Cerros orientales | - | Machine Observation | High | Medium | Medium |
| GBIF | 2016 | -73.36516 | 4.68115 | Cundinamarca | Cerros orientales | - | Machine Observation | High | Medium | Medium |
| GBIF | 2016 | -72.62890 | 4.39003 | Cundinamarca | Cerros orientales | - | Machine Observation | High | Medium | Low |
| GBIF | 2016 | -73.36516 | 4.68115 | Cundinamarca | Cerros orientales | - | Machine Observation | High | Medium | Medium |
| GBIF | 2016 | -72.17041 | 4.67934 | Cundinamarca | Cerros orientales | - | Machine Observation | High | Medium | Low |
| GBIF | 2014 | -76.06520 | 3.54653 | Valle Del Cauca | Corregimiento Toche, Paraje Los Chorros, predio Santa Ana de los Caballeros | - | Human Observation | Low | Medium | Medium |
| GBIF | 2014 | -76.17344 | 3.39675 | Valle Del Cauca | Corregimiento San Isidro, predio La Cuchilla | - | Human Observation | Low | Medium | Medium |
| GBIF | 2014 | -76.04387 | 3.58455 | Valle Del Cauca | Corregimiento Toche, Vereda Cabuyal adentro, paraje Los Chorros, predio Lusitania | - | Human Observation | Low | Medium | Medium |
| GBIF | 2014 | -76.12805 | 3.86951 | Valle Del Cauca | Corregimiento Crucero Nogales, Predio Rinconadas | - | Human Observation | Low | Medium | Medium |
| GBIF | 2013 | -77.24806 | 5.68050 | Choco | Corregimiento de Arusi | Nuqui | Human Observation | Low | Medium | Medium |
| GBIF | 1983 | -76.69547 | 5.09763 | Choco | Poblacion de Andogoya | - | Preserved Specimen | High | Medium | Medium |
| GBIF | 2003 | -75.39313 | 5.00881 | Caldas | Finca La Fe | - | Preserved Specimen | High | Medium | Medium |
| GBIF | 2001 | -75.02597 | 5.50247 | Caldas | Vereda La Cabana | - | Preserved Specimen | High | Medium | Medium |
| GBIF | 2016 | -73.79472 | 6.05639 | Santander | Vereda Ojo de Agua | - | Machine Observation | High | Medium | Medium |
| GBIF | 2016 | -73.79472 | 6.05639 | Santander | Vereda Ojo de Agua | - | Machine Observation | High | Medium | Medium |
| GBIF | 2016 | -73.79472 | 6.05639 | Santander | Vereda Ojo de Agua | - | Machine Observation | High | Medium | Medium |
| GBIF | 2016 | -73.79472 | 6.05639 | Santander | Vereda Ojo de Agua | - | Machine Observation | High | Medium | Medium |
| GBIF | 2016 | -73.79472 | 6.05639 | Santander | Vereda Ojo de Agua | - | Machine Observation | High | Medium | Medium |
| GBIF | 2016 | -73.79472 | 6.05639 | Santander | Vereda Ojo de Agua | - | Machine Observation | High | Medium | Medium |
| GBIF | 2016 | -73.79472 | 6.05639 | Santander | Vereda Ojo de Agua | - | Machine Observation | High | Medium | Medium |
| GBIF | 2016 | -73.79472 | 6.05639 | Santander | Vereda Ojo de Agua | - | Machine Observation | High | Medium | Medium |
| GBIF | 2016 | -73.79472 | 6.05639 | Santander | Vereda Ojo de Agua | - | Machine Observation | High | Medium | Medium |
| GBIF | 2016 | -73.79472 | 6.05639 | Santander | Vereda Ojo de Agua | - | Machine Observation | High | Medium | Medium |
| GBIF | 2016 | -73.79472 | 6.05639 | Santander | Vereda Ojo de Agua | - | Machine Observation | High | Medium | Medium |
| GBIF | 2016 | -73.79472 | 6.05639 | Santander | Vereda Ojo de Agua | - | Machine Observation | High | Medium | Medium |
| GBIF | 2016 | -73.79472 | 6.05639 | Santander | Vereda Ojo de Agua | - | Machine Observation | High | Medium | Medium |
| GBIF | 2016 | -73.79472 | 6.05639 | Santander | Vereda Ojo de Agua | - | Machine Observation | High | Medium | Medium |
| GBIF | 2016 | -73.79472 | 6.05639 | Santander | Vereda Ojo de Agua | - | Machine Observation | High | Medium | Medium |
| GBIF | 2016 | -73.79472 | 6.05639 | Santander | Vereda Ojo de Agua | - | Machine Observation | High | Medium | Medium |
| GBIF | 2016 | -73.79472 | 6.05639 | Santander | Vereda Ojo de Agua | - | Machine Observation | High | Medium | Medium |
| GBIF | 2016 | -73.79472 | 6.05639 | Santander | Vereda Ojo de Agua | - | Machine Observation | High | Medium | Medium |
| GBIF | 2016 | -73.79472 | 6.05639 | Santander | Vereda Ojo de Agua | - | Machine Observation | High | Medium | Medium |
| GBIF | 2016 | -73.79472 | 6.05639 | Santander | Vereda Ojo de Agua | - | Machine Observation | High | Medium | Medium |
| GBIF | 2016 | -73.79472 | 6.05639 | Santander | Vereda Ojo de Agua | - | Machine Observation | High | Medium | Medium |
| GBIF | 2016 | -73.79472 | 6.05639 | Santander | Vereda Ojo de Agua | - | Machine Observation | High | Medium | Medium |
| GBIF | 2016 | -73.79472 | 6.05639 | Santander | Vereda Ojo de Agua | - | Machine Observation | High | Medium | Medium |
| GBIF | 2016 | -73.79472 | 6.05639 | Santander | Vereda Ojo de Agua | - | Machine Observation | High | Medium | Medium |
| GBIF | 2016 | -73.81077 | 6.08862 | Santander | Vereda San Pablo | - | Machine Observation | High | Medium | Medium |
| GBIF | 2016 | -73.80056 | 6.06972 | Santander | Vereda Ojo de Agua | - | Machine Observation | High | Medium | Medium |
| GBIF | 2016 | -73.80056 | 6.06972 | Santander | Vereda Ojo de Agua | - | Machine Observation | High | Medium | Medium |
| GBIF | 2016 | -73.80056 | 6.06972 | Santander | Vereda Ojo de Agua | - | Machine Observation | High | Medium | Medium |
| GBIF | 2016 | -73.80056 | 6.06972 | Santander | Vereda Ojo de Agua | - | Machine Observation | High | Medium | Medium |
| GBIF | 2016 | -73.80056 | 6.06972 | Santander | Vereda Ojo de Agua | - | Machine Observation | High | Medium | Medium |
| GBIF | 2016 | -73.80056 | 6.06972 | Santander | Vereda Ojo de Agua | - | Machine Observation | High | Medium | Medium |
| GBIF | 2016 | -73.79472 | 6.05639 | Santander | Vereda Ojo de Agua | - | Machine Observation | High | Medium | Medium |
| GBIF | 2016 | -73.79472 | 6.05639 | Santander | Vereda Ojo de Agua | - | Machine Observation | High | Medium | Medium |
| GBIF | 2016 | -73.79250 | 6.04972 | Santander | Vereda Alto Gaital | - | Machine Observation | High | Medium | Medium |
| GBIF | 2016 | -73.81077 | 6.08862 | Santander | Vereda San Pablo | - | Machine Observation | High | Medium | Medium |
| GBIF | 2016 | -73.80056 | 6.06972 | Santander | Vereda Ojo de Agua | - | Machine Observation | High | Medium | Medium |
| GBIF | 2016 | -73.80056 | 6.06972 | Santander | Vereda Ojo de Agua | - | Machine Observation | High | Medium | Medium |
| GBIF | 2016 | -73.80056 | 6.06972 | Santander | Vereda Ojo de Agua | - | Machine Observation | High | Medium | Medium |
| GBIF | 2016 | -73.80056 | 6.06972 | Santander | Vereda Ojo de Agua | - | Machine Observation | High | Medium | Medium |
| GBIF | 2016 | -73.80056 | 6.06972 | Santander | Vereda Ojo de Agua | - | Machine Observation | High | Medium | Medium |
| GBIF | 2016 | -73.80056 | 6.06972 | Santander | Vereda Ojo de Agua | - | Machine Observation | High | Medium | Medium |
| GBIF | 2016 | -73.80056 | 6.06972 | Santander | Vereda Ojo de Agua | - | Machine Observation | High | Medium | Medium |
| GBIF | 2016 | -73.80056 | 6.06972 | Santander | Vereda Ojo de Agua | - | Machine Observation | High | Medium | Medium |
| GBIF | 2016 | -73.79472 | 6.05639 | Santander | Vereda Ojo de Agua | - | Machine Observation | High | Medium | Medium |
| GBIF | 2016 | -73.79250 | 6.04972 | Santander | Vereda Alto Gaital | - | Machine Observation | High | Medium | Medium |
| GBIF | 2016 | -73.79250 | 6.04972 | Santander | Vereda Alto Gaital | - | Machine Observation | High | Medium | Medium |
| GBIF | 2016 | -73.79250 | 6.04972 | Santander | Vereda Alto Gaital | - | Machine Observation | High | Medium | Medium |
| GBIF | 2016 | -73.79250 | 6.04972 | Santander | Vereda Alto Gaital | - | Machine Observation | High | Medium | Medium |
| GBIF | 2016 | -73.79250 | 6.04972 | Santander | Vereda Alto Gaital | - | Machine Observation | High | Medium | Medium |
| GBIF | 2013 | -73.12847 | 5.86881 | Boyaca | Reserva Municipal Rancheria | - | Human Observation | Low | Medium | Medium |
| GBIF | 1997 | -73.09870 | 6.13823 | Santander | Santander: Encino. | - | Preserved Specimen | High | Medium | Medium |
| GBIF | 2013 | -76.54139 | 3.78500 | Valle Del Cauca | Finca las Acacias, Vereda El Aguacate | - | Human Observation | Low | Medium | Medium |
| GBIF | 1959 | -77.01000 | 2.24000 | - | Departamento del Cauca | - | Preserved Specimen | High | Medium | Low |
| GBIF | 1992 | -76.11560 | 4.25500 | Valle Del Cauca | Uribe | - | Preserved Specimen | High | Medium | Medium |
| GBIF | 1959 | -76.11560 | 4.25500 | Valle Del Cauca | Uribe | - | Preserved Specimen | High | Medium | Medium |
| GBIF | 1992 | -76.91142 | 1.22919 | Putumayo | - | - | Preserved Specimen | High | Medium | Medium |
| GBIF | 1992 | -76.91142 | 1.22919 | Putumayo | - | - | Preserved Specimen | High | Medium | Medium |
| GBIF | 1992 | -75.82000 | 6.86000 | Antioquia | - | - | Human Observation | Low | Medium | Medium |
| GBIF | 1992 | -75.68000 | 6.92000 | Antioquia | - | - | Human Observation | Low | Medium | Medium |
| GBIF | 1992 | -75.82000 | 5.60000 | Antioquia | - | - | Human Observation | Low | Medium | Medium |
| GBIF | 1992 | -75.15000 | 7.07000 | Antioquia | - | - | Human Observation | Low | Medium | Medium |
| GBIF | 1992 | -75.88000 | 5.66000 | Antioquia | - | - | Human Observation | Low | Medium | Medium |
| GBIF | 1992 | -75.91000 | 6.73000 | Antioquia | - | - | Human Observation | Low | Medium | Medium |
| GBIF | 1992 | -75.52000 | 6.57000 | Antioquia | - | - | Human Observation | Low | Medium | Medium |
| GBIF | 1992 | -75.40000 | 6.49000 | Antioquia | - | - | Human Observation | Low | Medium | Medium |
| GBIF | 1992 | -75.46000 | 6.65000 | Antioquia | - | - | Human Observation | Low | Medium | Medium |
| GBIF | 1992 | -75.61000 | 5.75000 | Antioquia | - | - | Human Observation | Low | Medium | Medium |
| GBIF | 1992 | -75.79000 | 5.80000 | Antioquia | - | - | Human Observation | Low | Medium | Medium |
| GBIF | 1992 | -75.42000 | 6.97000 | Antioquia | - | - | Human Observation | Low | Medium | Medium |
| GBIF | 1992 | -75.72000 | 5.67000 | Antioquia | - | - | Human Observation | Low | Medium | Medium |
| GBIF | 1992 | -75.67000 | 6.61000 | Antioquia | - | - | Human Observation | Low | Medium | Medium |
| GBIF | 1992 | -75.29000 | 6.73000 | Antioquia | - | - | Human Observation | Low | Medium | Medium |
| GBIF | 1992 | -75.07000 | 6.91000 | Antioquia | - | - | Human Observation | Low | Medium | Medium |
| GBIF | 1958 | -76.95000 | 2.66667 | Cauca | Charguayaco | - | Preserved Specimen | High | Medium | Medium |
| GBIF | 1955 | -76.41667 | 2.40000 | Cauca | La Quintana | - | Preserved Specimen | High | Medium | Medium |
| GBIF | 1958 | -76.40000 | 2.50000 | Cauca | Totoro | - | Preserved Specimen | High | Medium | Medium |
| GBIF | 1952 | -74.08333 | 4.60000 | Cundinamarca | Bogota | - | Preserved Specimen | High | Medium | Medium |
| GBIF | 1952 | -74.08333 | 4.60000 | Cundinamarca | Bogota | - | Preserved Specimen | High | Medium | Medium |
| GBIF | 1952 | -74.08333 | 4.60000 | Cundinamarca | Bogota | - | Preserved Specimen | High | Medium | Medium |
| GBIF | 1951 | -75.98333 | 1.61667 | Huila | San Adolfo | - | Preserved Specimen | High | Medium | Low |
| GBIF | 1955 | -76.88333 | 2.53333 | Cauca | Sabanetas | - | Preserved Specimen | High | Medium | Medium |
| Peer-reviewed article | 1992 | -72.19200 | 6.50994 | Boyaca | - | Guican | Machine Observation | High | High | High |
| Peer-reviewed article | 1992 | -73.28973 | 5.18221 | Boyaca | - | Chinavita | Machine Observation | High | High | High |
| Peer-reviewed article | 1992 | -73.77065 | 4.58107 | Cundinamarca | - | Fomeque | Machine Observation | High | High | High |
| Peer-reviewed article | 1992 | -73.59292 | 2.63652 | Meta | - | Vistahermosa | Machine Observation | High | High | High |
| Peer-reviewed article | 1992 | -75.31270 | 4.77971 | Tolima | - | Murillo | Machine Observation | High | High | High |
| Peer-reviewed article | 1992 | -75.51656 | 4.69607 | Risaralda | - | Pereira | Machine Observation | High | High | High |
| Peer-reviewed article | 1992 | -77.37225 | 1.19901 | Narino | - | Consaca | Machine Observation | High | High | High |
| Peer-reviewed article | 1992 | -73.96443 | 5.39533 | Cundinamarca | - | Carmen De Carupa | Machine Observation | High | High | High |
| Peer-reviewed article | 1992 | -75.31623 | 4.91322 | Tolima | - | Casabianca | Machine Observation | High | High | High |
| Peer-reviewed article | 1992 | -76.54513 | 3.71268 | Valle Del Cauca | - | La Cumbre | Machine Observation | High | High | High |
| Peer-reviewed article | 1992 | -76.40333 | 2.51214 | Cauca | - | Totoro | Machine Observation | High | High | High |
| Peer-reviewed article | 1992 | -75.91177 | 2.28526 | Huila | - | La Plata | Machine Observation | High | High | High |
| Peer-reviewed article | 1992 | -75.75107 | 1.61409 | Caqueta | - | Florencia | Machine Observation | High | High | High |
| Peer-reviewed article | 1992 | -73.76592 | 2.21909 | Meta | - | La Macarena | Machine Observation | High | High | High |
| Peer-reviewed article | 1992 | -72.92459 | 6.11377 | Santander | - | Coromoro | Machine Observation | High | High | High |
