# Supplementary material for: Spotting what’s important: Priority areas, connectivity, and conservation of the Northern Tiger Cat (Leopardus tigrinus) in Colombia
Source: PLoS One. 2022 Sep 13;17(9):e0273750. doi: 10.1371/journal.pone.0273750 (PMC9469974; doi:10.1371/journal.pone.0273750)
Supplement: S2 Table — (DOCX) [file pone.0273750.s004.docx]

***Spot*ting what´s important: priority areas, connectivity, and conservation of the Northern Tiger Cat (*Leopardus tigrinus*) in Colombia**

José F. González-Maya, Diego A. Zárrate-Charry, Andrés Arias-Alzate, Leonardo Lemus-Mejía, Angela P. Hurtado-Moreno, Magda Gissella Vargas-Gómez, Teresa Andrea Cárdenas, Victor Mallarino, Jan Schipper

**SUPPORTING INFORMATION**

**S3 Table**

**Supporting Information 3 (****S3 Table).** Spearman correlation tests for bioclimatic variables used for the distribution model of *Leopardus tigrinus* in Colombia.

| **Variables** | **Annual Mean Temperature** | **Annual Precipitation** | **Precipitation of Wettest Month** | **Precipitation of Driest Month** | **Precipitation Seasonality** | **Mean Diurnal Range** | **Temperature Seasonality** | **Elevation** |
| --- | --- | --- | --- | --- | --- | --- | --- | --- |
| **Annual Mean Temperature** |  | 0 | 0 | 0 | 0 | 0.457396 | 0 | 0 |
| **Annual Precipitation** | 0.16822 |  | 0 | 0 | 0 | 0 | 1.08E-07 | 0 |
| **Precipitation of Wettest Month** | 0.407697 | 0.774524 |  | 0 | 0 | 0 | 0 | 0 |
| **Precipitation of Driest Month** | -0.44776 | 0.561542 | 0.092806 |  | 0 | 3.15E-07 | 0 | 0 |
| **Precipitation Seasonality** | 0.539447 | -0.386 | 0.125333 | -0.93712 |  | 3.82E-12 | 0 | 0 |
| **Mean Diurnal Range** | 0.007433 | -0.15548 | -0.09211 | -0.05112 | 0.069366 |  | 0 | 0 |
| **Temperature Seasonality** | 0.482455 | 0.053101 | 0.245837 | -0.52445 | 0.556042 | -0.18902 |  | 0 |
| **Elevation** | -0.9012 | -0.30184 | -0.44628 | 0.224334 | -0.3313 | 0.12413 | -0.32735 |  |
