# Supplementary material for: Spotting what’s important: Priority areas, connectivity, and conservation of the Northern Tiger Cat (Leopardus tigrinus) in Colombia
Source: PLoS One. 2022 Sep 13;17(9):e0273750. doi: 10.1371/journal.pone.0273750 (PMC9469974; doi:10.1371/journal.pone.0273750)
Supplement: S3 Table — (DOCX) [file pone.0273750.s005.docx]

***Spot*ting what´s important: priority areas, connectivity, and conservation of the Northern Tiger Cat (*Leopardus tigrinus*) in Colombia**

José F. González-Maya, Diego A. Zárrate-Charry, Andrés Arias-Alzate, Leonardo Lemus-Mejía, Angela P. Hurtado-Moreno, Magda Gissella Vargas-Gómez, Teresa Andrea Cárdenas, Victor Mallarino, Jan Schipper

**SUPPORTING INFORMATION**

**S5 Table**

**Supporting Information 5** **(S5 Table).** Models constructed for species distribution modeling and corresponding parameters for *Leopardus tigrinus* in Colombia.

| **Settings** | **Features** | **Rm** | **Train.**  **AUC** | **Avg.**  **Test.Auc** | **Var.**  **Test.Auc** | **Avg.**  **Diff.Auc** | **Var.**  **Diff.Auc** | **Avg.**  **Test.Ormtp** | **Var.**  **Test.Ormtp** | **Avg.**  **Test.Or10pct** | **Var.**  **Test.Or10pct** | **AICc** | **Delta**  **AICc** | **wAic** | **Parameters** |
| --- | --- | --- | --- | --- | --- | --- | --- | --- | --- | --- | --- | --- | --- | --- | --- |
| LQHP_2.5 | LQHP | 2.5 | 0.8902 | 0.874673 | 0.002724 | 0.026256 | 0.002146 | 0.01125 | 0.000173 | 0.106607 | 0.001389 | 4323.541 | 0 | 0.353637 | 16 |
| LQHP_2 | LQHP | 2 | 0.8923 | 0.874778 | 0.00295 | 0.028355 | 0.002342 | 0.0175 | 0.000142 | 0.106607 | 0.001389 | 4323.61 | 0.06944 | 0.341569 | 19 |
| LQHP_3 | LQHP | 3 | 0.8895 | 0.875305 | 0.002559 | 0.023798 | 0.001997 | 0.0175 | 0.000558 | 0.106607 | 0.001389 | 4323.97 | 0.429114 | 0.285349 | 14 |
| LQ_0.5 | LQ | 0.5 | 0.8847 | 0.874637 | 0.001981 | 0.024037 | 0.000606 | 0.0125 | 0.000208 | 0.121607 | 0.001225 | 4330.239 | 6.697889 | 0.01242 | 9 |
| LQ_1 | LQ | 1 | 0.8839 | 0.876942 | 0.00163 | 0.021518 | 0.000503 | 0.0125 | 0.000208 | 0.121607 | 0.001225 | 4331.958 | 8.416819 | 0.005259 | 9 |
| LQ_1.5 | LQ | 1.5 | 0.8841 | 0.877081 | 0.001454 | 0.019804 | 0.000477 | 0.0125 | 0.000208 | 0.116607 | 0.001079 | 4334.46 | 10.91907 | 0.001505 | 9 |
| LQ_2 | LQ | 2 | 0.884 | 0.876404 | 0.001343 | 0.018824 | 0.000484 | 0.0125 | 0.000208 | 0.116607 | 0.001079 | 4338.309 | 14.76807 | 0.00022 | 9 |
| LQH_3 | LQH | 3 | 0.8878 | 0.881167 | 0.001288 | 0.015907 | 0.000484 | 0.01125 | 0.000173 | 0.111607 | 0.001134 | 4342.631 | 19.09051 | 2.53E-05 | 18 |
| LQ_2.5 | LQ | 2.5 | 0.883 | 0.876049 | 0.001284 | 0.018109 | 0.000478 | 0.01125 | 0.000173 | 0.110357 | 0.001096 | 4345.381 | 21.84031 | 6.4E-06 | 10 |
| LQH_2 | LQH | 2 | 0.8889 | 0.882129 | 0.001393 | 0.018577 | 0.000463 | 0.00625 | 0.000156 | 0.111607 | 0.001134 | 4345.788 | 22.24686 | 5.22E-06 | 24 |
| LQH_2.5 | LQH | 2.5 | 0.8884 | 0.881681 | 0.001338 | 0.01685 | 0.000463 | 0.01125 | 0.000173 | 0.111607 | 0.001134 | 4347.117 | 23.57611 | 2.69E-06 | 22 |
| LQ_3 | LQ | 3 | 0.8821 | 0.876459 | 0.001205 | 0.016729 | 0.00049 | 0.01125 | 0.000173 | 0.110357 | 0.001096 | 4347.689 | 24.14844 | 2.02E-06 | 9 |
| LQHP_1.5 | LQHP | 1.5 | 0.8961 | 0.875684 | 0.002806 | 0.030117 | 0.002242 | 0.0175 | 0.000142 | 0.106607 | 0.001389 | 4358.197 | 34.65645 | 1.05E-08 | 35 |
| LQH_1.5 | LQH | 1.5 | 0.8922 | 0.881352 | 0.001514 | 0.021561 | 0.00054 | 0.00625 | 0.000156 | 0.111607 | 0.001134 | 4374.467 | 50.92613 | 3.09E-12 | 37 |
| L_2.5 | L | 2.5 | 0.8673 | 0.865645 | 0.001471 | 0.01181 | 0.001255 | 0.005 | 0.0001 | 0.114405 | 0.002671 | 4393.278 | 69.73732 | 2.54E-16 | 4 |
| L_3 | L | 3 | 0.8675 | 0.865759 | 0.001449 | 0.011753 | 0.001243 | 0.005 | 0.0001 | 0.114405 | 0.002671 | 4393.376 | 69.83516 | 2.42E-16 | 4 |
| L_0.5 | L | 0.5 | 0.868 | 0.865482 | 0.001541 | 0.01282 | 0.001264 | 0.005 | 0.0001 | 0.108155 | 0.003068 | 4393.991 | 70.45029 | 1.78E-16 | 5 |
| L_1 | L | 1 | 0.8679 | 0.86555 | 0.00153 | 0.012558 | 0.00126 | 0.005 | 0.0001 | 0.108155 | 0.003068 | 4394.188 | 70.64744 | 1.61E-16 | 5 |
| L_1.5 | L | 1.5 | 0.8678 | 0.865508 | 0.001518 | 0.012307 | 0.001263 | 0.005 | 0.0001 | 0.108155 | 0.003068 | 4394.5 | 70.95935 | 1.38E-16 | 5 |
| L_2 | L | 2 | 0.8676 | 0.86549 | 0.00149 | 0.012024 | 0.001258 | 0.005 | 0.0001 | 0.108155 | 0.003068 | 4394.937 | 71.39602 | 1.11E-16 | 5 |
| LQHP_1 | LQHP | 1 | 0.9019 | 0.875634 | 0.003037 | 0.034296 | 0.00247 | 0.0175 | 0.000142 | 0.111607 | 0.001134 | 4403.405 | 79.86453 | 1.61E-18 | 52 |
| H_3 | H | 3 | 0.8902 | 0.883245 | 0.001633 | 0.018041 | 0.000546 | 0.00625 | 0.000156 | 0.111607 | 0.001134 | 4443.245 | 119.7037 | 3.59E-27 | 52 |
| H_2 | H | 2 | 0.891 | 0.884773 | 0.001699 | 0.018266 | 0.000578 | 0.00625 | 0.000156 | 0.111607 | 0.001134 | 4449.225 | 125.6846 | 1.81E-28 | 56 |
| H_2.5 | H | 2.5 | 0.8907 | 0.883865 | 0.001701 | 0.018367 | 0.000585 | 0.00625 | 0.000156 | 0.111607 | 0.001134 | 4458.791 | 135.2505 | 1.51E-30 | 57 |
| LQH_1 | LQH | 1 | 0.8969 | 0.880289 | 0.001696 | 0.027384 | 0.00076 | 0.00625 | 0.000156 | 0.116607 | 0.001079 | 4489.975 | 166.4347 | 2.56E-37 | 68 |
| H_1.5 | H | 1.5 | 0.8916 | 0.884452 | 0.001727 | 0.019961 | 0.000557 | 0.00625 | 0.000156 | 0.111607 | 0.001134 | 4509.216 | 185.675 | 1.7E-41 | 69 |
| H_1 | H | 1 | 0.8959 | 0.882042 | 0.001857 | 0.026517 | 0.000721 | 0.00625 | 0.000156 | 0.111607 | 0.001134 | 4563.68 | 240.1391 | 2.53E-53 | 80 |
| LQHP_0.5 | LQHP | 0.5 | 0.9085 | 0.875109 | 0.003272 | 0.042186 | 0.002795 | 0.023452 | 5.61E-06 | 0.129107 | 0.001984 | 4584.596 | 261.0547 | 7.27E-58 | 87 |
| H_0.5 | H | 0.5 | 0.9042 | 0.881055 | 0.001977 | 0.032308 | 0.001084 | 0.00625 | 0.000156 | 0.111607 | 0.001134 | 4735.032 | 411.4916 | 1.56E-90 | 101 |
| LQH_0.5 | LQH | 0.5 | 0.9041 | 0.879988 | 0.001948 | 0.032906 | 0.001115 | 0.00625 | 0.000156 | 0.111607 | 0.001134 | 4814.167 | 490.6265 | 1E-107 | 107 |
