# Supplementary material for: Spotting what’s important: Priority areas, connectivity, and conservation of the Northern Tiger Cat (Leopardus tigrinus) in Colombia
Source: PLoS One. 2022 Sep 13;17(9):e0273750. doi: 10.1371/journal.pone.0273750 (PMC9469974; doi:10.1371/journal.pone.0273750)
Supplement: Attachment — Submitted filename: PONE-D-21-29326_R1_2.pdf [file pone.0273750.s006.pdf]

PLOS ONE

Spotting what’s important: priority areas, connectivity, and conservation of the Northern Tiger Cat (Leopardus tigrinus) in Colombia

--Manuscript Draft--

**Manuscript Number:**	PONE-D-21-29326R1
**Article Type:**	Research Article
**Full Title:**	Spotting what’s important: priority areas, connectivity, and conservation of the Northern Tiger Cat (Leopardus tigrinus) in Colombia
**Short Title:**	Leopardus tigrinus in Colombia
**Corresponding Author:**	José F. González-Maya, Ph.D.<br>Universidad Autonoma Metropolitana Unidad Lerma<br>Lerma de Villada, Mexico MEXICO
**Keywords:**	species distribution modeling; Oncilla; Tigrina; connectivity; Andes
**Abstract:**	Leopardus tigrinus is among the least known carnivore species in the Andes, including considerable taxonomic uncertainty. Mostly known from Brazil (currently suggested as two distinct species), the Andean and isolated Central American forms remain among the least understood carnivores in the Neotropics. Here we model the distribution, connectivity and overlap with existing conservation areas for the species in Colombia. Using a Species Distribution Modeling approach, we estimated current potential range of the species in Colombia and identified potential habitat blocks remaining in the country. In addition, we designed a connectivity network across the available cores, using a circuit theory approach, to evaluate habitat linkage. Finally, we defined a prioritization scheme for the remaining habitat cores and assessed the level of coverage of protected areas for the country. L. tigrinus is potentially present across the three Andean branches of Colombia, with still considerable continuous habitat cores, mostly located on the eastern and central Andean ranges. Most habitat cores are theoretically connected, but nearly 15% are isolated. Priority areas were located across the eastern and central ranges, but with very significant and promising cores in the northern eastern and western ranges. Current level of protection indicates nearly 30% of the range is “protected”, but only about 25% is under national strict protected areas. Evolution of this coverage showed some periods of significant increase but interestingly the number of cores grew at a faster rate than overall proportion protected, likely indicating numerous discontinuous fragments, and not contiguous functional landscapes. This represents the most updated assessment of the distribution and conservation status for the species in Colombia, and indicates the numerous conservation opportunities, especially in most populated areas of the country. We found unique business environmental passive’s opportunities, including compensation and development potential, which are becoming more available in the country.
**Order of Authors:**	José F. González-Maya, Ph.D.
	Diego A. Zárrate-Charry
	Andrés Arias-Alzate
	Leonardo Lemus-Mejía
	Angela P. Hurtado-Moreno
	Magda Gissella Vargas-Gómez
	Teresa Andrea Cárdenas
	Victor Mallarino
	Jan Schipper
**Response to Reviewers:**	Responses to reviewers<br><br>JOURNAL MANAGER NEW NOTE ADDED 07/12/2022:<br>Dear Freddie Domini,

Powered by Editorial Manager® and ProduXion Manager® from Aries Systems Corporation

We added the corresponding credits to all captions in the MS.

Best,

José

Reviewer #1
The manuscript entitled “Spotting what’s important: priority areas, connectivity, and conservation of the Oncilla (Leopardus tigrinus) in Colombia” focuses on assessing the distribution of the species Leopardus tigrinus in Colombia, with the development of distribution models and connectivity analyses to assess the conservation status of habitats identified as priorities for the species. The manuscript presents current and sophisticated analyses for achieving the objectives and is well written overall. However, I consider the main contribution of the paper to be the development of a useful methodology for assessing the habitats and protection status of the species that could be easily incorporated in other areas of its distribution, as well as for several other threatened species. Despite the importance and relevance of the theme of the manuscript, I feel that some changes and elucidations are necessary for the article to progress through the review process. Below are my comments.
R/Thanks for the comments.

Lines 69-78: You need to consider the current taxonomic classification recognized for the “L. tigrinus complex”. Today we have a general recognition of the species Leopardus guttulus in the southern portion of the range of this species’ complex (Trigo et al. 2013, Kitchener et al. 2017, Nascimento & Feijó 2017, de Oliveira, T., Trigo, T., Tortato, M., Paviolo, A., Bianchi, R. & Leite-Pitman, M.R.P. 2016. Leopardus guttulus. The IUCN Red List of Threatened Species 2016). Thus, the information about it being one of the most widely distributed species in the Americas (lines 69-70) and the distribution shown in lines 73-74 are not up to date, or even correct. The lack of consensus on the taxonomy and number of species in the northern part of this species’ complex distribution does exist (see Nascimento & Feijó 2017 and Kitchener et al. 2017), nevertheless both studies recognize the Colombian cat as Leopardus tigrinus. I suggest a revision of these articles and a rewording of this paragraph. I also suggest reviewing the popular name recognized for this species in these bibliographies, oncilla is commonly used for the central American subspecies/population, while northern tigrina or northern tiger cat is most often cited for L. tigrinus as a whole.
R/Thanks for the comment, we modified accordingly. We updated the distribution according to one of the most recent papers and added “disclaimers” that still the discussion keeps going about the taxonomy and thus distribution. Regarding the common name, we followed the suggestion and changed accordingly across the manuscript.

Line 82: Review: Brazil has two or three species, depending on the classification assumed, with which one are you comparing the population of Colombia?
R/Corrected and eliminated the reference (see comment by Rev2).

Line 122: please, include the time range of the collected records, the information is in the supplementary material but in my opinion it should be included here as well.
R/Added.

Lines 123-131: I think this part of the manuscript including information about filtering, and especially quality control of the data needs to be better explained. What is the three-step filtering? You said that you generated a categorization scheme for each record including 3 categories: credibility, quality and geographic accuracy. Would this be the 3 steps of filtering? I don't see this categorization as a 3 step process because it is not sequential. I would like to see a table or a complete listing of what you considered within each category, because with the incomplete description as it is in the manuscript now (only examples are presented), several doubts arise about this categorization process. As an example: for credibility, information on the type of evidence collected includes examples as footprint and photograph. For quality, a photographic record was exemplified. That is, we have photography in both categories. Still collected specimen and human observation wouldn't fit into evidence type? Moreover, for credibility, two types of information were used: source and evidence, in

this case, each received a categorization between low to high? If yes, how was the final category defined?
R/Thanks for the comment, and the reviewer was correct that it was confusing. We modified and provided details in the text and in the SI also a suggested by Rev2.

Lines 132-135: Please better explain this evaluation of the geographic precision. Did you have the geographic coordinates for all records? Here it seemed to me so that the only information evaluated was the location details as department or municipality reported in the source. If all records had coordinates, the evaluation of this less accurate information does not seem very useful to me. I see the importance of this evaluation if the records had variable information of origin, including from just a department of origin (in this case being of low precision) to specific coordinates taken on the collection site (in this case high precision).
R/We added a detailed explanation of the procedure. Although it is understandable the comment by the reviewer, regarding why double-checking locality information does not seem useful, we have vast experience with Colombia biodiversity data, where coordinates are often poorly taken or irresponsibly managed, and still, it makes it to GBIF through the Colombian System of Biodiversity Information (many from public institutions with low technical capacity). This way, we secure that these coordinates are precise and accurate; although we might lose some extra records, we make sure the data we used for the model is the best available.

Line 133: ...species ranging on the three ranges... would not be three branches? The three described before for the northern part of the mountain range?
R/Wrong line, but corrected.

Line137: …we then applied a moderate filter… Why did you consider it moderate if records with any category ranked as “low” were excluded?
R/We added an explanation for the decision and a citation.

Lines 143-146: Was this done to create a mask of the area that would be used in the modeling analyses? If yes, I would suggest using “mask/polygon/or sampling area” instead “calibration area”.
R/No, it was specifically for defining the Mobility Area of the species for the modelling; we added a citation where it is detailed.

Line149: I believe that reference 48 cited here does not include bioclimatic variables in its analyses.
R/Thanks, removed.

Line155: Please include some references for this statement “…given its potential influence on species distribution models, especially for the Andean region.”
R/Added.

Lines 160-163: You used the software Wallace which includes both Maxent and ENMeval, and you also said that Maxent is the most widely used algorithm, but you did not inform if you used this or both. Please, include this information.
R/We reworded for clarity. We refer to the algorithm and not the specific software (which is included in Wallace.

Line 178-184: This paragraph is confusing and with repeated information, here is a suggested text: “Once a binary map was obtained from the best model, we evaluated the different types of vegetation cover included in the suitable areas according to available information on species’ use. All coverage catalogued as natural by the national cartography dataset and with existing evidence of use by the species from literature and the distribution records, was selected within the binary map.”
R/Thank you so much for the suggestion. We modified the text using your suggestion.

Lines 184-187: “Once all coverages were extracted, we defined an area threshold for selecting those remaining patches that could be used by the species either as marginal or core habitats, based on a conservative value estimated from the mean home range reported for the species of 17km2.” I don´t understand that. What threshold was this? It was used to define what would be considered as marginal or core habitats? How? Please, explain better how and why this was done.

Powered by Editorial Manager® and ProduXion Manager® from Aries Systems Corporation

R/We reworded to make it clearer.

Line187-188: “We also selected 9 km as a maximum dispersal threshold for the species, also based on previous ecological studies [15, 17, 41, 59].” What exactly was this information used for? Was it used for the identification of core and marginal habitats? Explain.
R/We added details.

Lines 189-190: Delete: “The final output included all available natural habitats within the species potential distribution in the country.” Redundant information.
R/Eliminated

Line192: Once all available remaining habitats were selected, we designed an ecological connectivity network analysis across the species distribution based on a circuit theory…” replace “selected” by “identified” and “the species distribution” by “recognized patches”.
R/Changed.

Lines 196-198: “We defined as core habitats the available habitats previously identified, and generated a resistance layer based on a the most updated available “human footprint index” developed for Colombia [62]”
Change to “We consider as core habitats the recognized habitat patches remaining within the suitable areas identified in the species’ distribution model. The resistance layer was generated based on the available “human footprint index” developed for Colombia.”
R/Thanks for the suggestion. Corrected accordingly.

Still about the central areas, I would like to see more information about these, especially how many were exactly identified? Couldn't you identify them in a figure? I believe that information about the size and location of these areas is fundamental. In lines 300-301 you say that all but 27 core areas had at least one connection with other areas, so I just understand that you have more than 27, but I don't know how many. Are they the 183 fragments mentioned in line 1*80? Please make this information clearer.
R/We reworded for clarity. Anyway earlier in the text we mentioned: “After identifying the available natural coverages within these suitable areas, we found the potentially occupied areas to be only 91,209.85 km2 (~39.8% of the SDM), distributed in 183 patches, with a mean (±SD) area of 498.41±2783.58 km2 across the SDM (Fig 2A).” Furthermore, we find this comment odd given that we have those patches represented in Figures 1, 2, 3,4 and 6, characterizing their area, distribution, centrality, priority and overlap with protected areas.

Line 202: delete “cover” from “land use cover”.
R/Corrected.

Line 204: delete “specific” from “specific species-specific”.
R/Corrected.

Lines 210-211: The information present in lines 187-188 should be here.
R/Corrected.

Lines 214-215: Please include a brief description of what the core value and potential current flow would be. Even if you cite papers that refer to these analyses/indexes, your paper should provide enough information so that the reader can understand what these indexes would be and why they were made.
R/We added the corresponding description.

Lines 231-233: Could you present more specifically the value classes considered in each category? I think this would be important so that other people can use the same methodology.
R/We added details to the text.

Lines 239-240: Consider that these estimates are all for the currently recognized species Leopardus guttulus. You are working with another species, so a sentence

including acknowledgement that these estimates may be quite different for the species in Colombia is essential. I understand that this is the only information available for a closer species, but there should be a sentence highlighting this possible bias in your analyses.
R/Thanks for the comment, we added the corresponding acknowledgement of the bias.

Line 251: "…in terms of total covered area and number of cores or core fragments included." Delete "cores or".
R/Deleted.

Line255: please, consider to change "used" by "included".
R/Corrected.

Line 255: Our model used 15 ecorregions… Weren't these ecoregions included before generating the models? In the mask area? Wouldn't it be better: "Our sampling area included 15 ecoregions"?
R/Corrected and adjusted accordingly.

Lines 261-265: did you not present the results of the correlation between the variables. Was there a correlation? Which ones were excluded from the analyses?
R/We added the details of the correlation tests.

Lines 261-265: I would like to read or see some information about the behavior of the each variable, e.g.: did elevation have a positive or negative relationship with the probability of species' occurrence?
R/We added details to the text and added the response curves to the SI.

Line 270: "climatic suitable areas", It's not just climatic, is it? Elevation was also used as a variable to run the models, wasn't it?
R/Changed for environmentally.

Lines 271-273: "However, given the huge natural cover transformation processes in the main distribution of the species…". Consider rephrasing this sentence making it clear that you are referring to transformation due to human activities.
R/Corrected.

Line 278: replace "after selecting" by "after identifying". Also delete "climatically", your model was not based exclusively on climatic variables, right?
R/Changed

Line 282: delete "climatic"
R/Deleted.

Lines 284-286: "However, most remaining habitat patches are concentrated towards the smaller sizes while very few approach the largest sizes (Fig2B)." change to "Most remaining habitat patches were concentrated towards the smaller size, with very few close to the largest one."
R/Corrected.

Lines 304-307: Rephrase as: "Centrality values for all cores also varied considerably, with a mean value of 981.53±1128.85 for the entire network, but with an interesting geographical variation: overall centrality value was closely related with the size of the core (T=16.40, p<0.001, R2Adj=0.60; Fig.3B)."
R/Corrected.

Lines 304-313: Without knowing exactly what the central values mean it is difficult to understand how interesting these results are. I believe that including a description of this analysis/index is fundamental to understanding the results.
R/More details were added to the methods section.

Lines 323-324: Only in this part of the results do we have an indication of what the centrality values are, that is, the importance of the area/fragment for maintaining connectivity. This information needs to be included in the methodology when this index is presented.

R/ More details were added to the methods section.

Lines 326-327: Call figure 4B at the end of this sentence.'
R/Added.

Lines 330-331: "The main core areas that currently retain ideal conditions for the species (Category 1) are in the central…". Indicate here that you are talking only about the first category, the most preserved one.
R/Added

Lines 343-344: "Our estimation of mean number of individuals, based on previous calculations for a closely related species, Leopardus guttulus, …". Taking into account that the existing estimates are in areas where we currently recognize as belonging to L. guttulus.
R/Corrected.

In lines 239-240 you said that density estimates are only available for Brazil, citing references 15 (de Oliveira TG, Tortato MA, Bonjorne de Almeida L, de Campos CB, Beisiegel BM. 2013), 17 (Oliveira-Santos LGR, Graipel ME, Tortato MA, Zucco CA, Cáceres NC, Goulart FVB. 2012) and 59 (Tortato M, de Oliveira T. 2005) and that you used these reports to calculate an expected number of individuals to Colombia.
At Table 1 you presented 4 estimates, citing only two references that do not completely agree with those listed in the methodology (15 and 72). Which estimates and references were actually used? Please verify this.
Also in Table 1, the column for average estimated population refers to density in what area? The total area of Colombia, of the SDM, or the area calculated after the identification of the available natural coverages within your model?
R/Thanks for the comment and for pointing out our mistake and also we reworded the caption respond to the last remark. We changed the text and the caption of the table to make sure it is clear that we did all our estimations for the core habitats located within the potential distribution (not the entire territory of Colombia, although it is stated from the methods section). Also, we added the missing citation to the methods section; we also clarify that we used all the available data to select the highest, typicial and fragmented estimates and then the mean estimate using all these studies to make our calculations, thus the use of all these sources although not cited in the table.

Lines 347-348: "This means a large variation in the number of potential individuals present on each core habitat across the country (Table 1)." Table 1 does not show this, i.e., the variation in the number of potential individuals in each core area. The table shows the values of population estimates that exist in the literature for other areas, am I wrong?
R/Correct! Thanks for pointing that out. We have a Figure afterwards to show the variation but reworded for clarity before table1. Also we added a detailed explanation in the methods.

Lines 348-349: "Accordingly, the number of individual oncilla estimated for Colombia is 10,375±4674 individuals…" As I highlighted above, was the area considered for this estimation the total area of Colombia or the areas resulting from your analyses (areas of potential occurrence)? I think it makes much more sense to calculate for the area estimated in the paper from your models.
R/Absolutely right, modified the phrase to make it clearer and added details to the methods.

Lines 350-352: delete "~" from the three values presented here.
R/Corrected.

Lines 397-406: I suggest reviewing this introductory paragraph with the questions I raised about the recognition of L. guttulus as a distinct species.
R/Agreed and corrected.

Line412-413: In agreement with I cited before, the IUCN red list already recognizes L. guttulus as a distinct species from L. tigrinus.
R/We checked the paragraph but we believe there is no reason to call L. guttulus assessment. This paragraph only says that we don´t now much, about the species, that

Powered by Editorial Manager® and ProduXion Manager® from Aries Systems Corporation

is catalogued as Vulnerable by IUCN (the same year L. guttulus was assessed and by the same author) and that we present new information.

Lines 421-427: Rewrite considering L. guttulus as a distinct species.
R/Changed accordingly.

Lines 444-448: Suggested sentence change: " Furthermore, a significant number of cores are represented on very small areas, mostly private reserves (i.e., Reservas de la Sociedad 446 Civil), that represent a very important conservation strategy [93], which by themselves contribute very little to the overall population conservation goals, but act as important stepping stones between main habitat core."
R/Changed as suggested.

Lines 460-464: This sentence is long and confusing, please consider rewriting it.
R/Corrected.

Lines 464-467: Like corridor implementation? I missed a discussion of ecological corridors between the core areas. These are included in your objectives and you present the development of specific analyses for the identification of these corridors.
R/We added some specific lines across the discussion to support the approach.

Lines 483-489: Very long sentence, consider a division to make the text more fluid.
R/Corrected.

Supporting Information 1: Please, include at the end of the table the description of the abbreviations in the columns U_Time, U_Credibility, U_Geographic and U_obso. Aslo a brief description of what each one of them would be, especially for U_Time and U_Obso.
R/Added.

Supporting Information 2: This table could be ordered by the AICc value making it easier to follow.
R/Changed accordingly.

Reviewer #2
Dear editor and authors,
this paper presents important information for the conservation of Leopardus tigrinus in Colombia. I have carefully read the version of the manuscript and found it quite interesting. However, there are some points that need improvement.
R/Thanks for the comments.

Introduction
- Line 65: Add a comma after "in fact".
R/Added.

- Line 69-78: Although the point of the manuscript is not to make a big review of the tigrina complex, the review presented is missing important references and present some wrong information:

- Lines 75-76: For example, the Brazilian tigrinas were divided in two species by Trigo et al. (2013) - Current Biology, and not by Nascimento and Feijó.
Trigo, T., Schneider, A., Lehugeur, L., Silveira, L., Freitas, T. O., & Eizirik, E. (2013). Molecular data reveal complex hybridization and a cryptic species of Neotropical wild cat. Current Biology, 23(24), 2528-2533.
R/Added.

- Line 75: Still, references 17 and 18 cited in this paragraph refers to studies developed in the Atlantic Forest. This region may not be at such high altitudes as the Andes, but is definitively not known as lowlands, as a great portion of it is located in mountainous regions that can reach more than 2,000 meters. Therefore, I suggest changing these terms
R/Changed.

- Line 72: Another important reference missing is Trindade et al. (2021), that reviewed

Powered by Editorial Manager® and ProduXion Manager® from Aries Systems Corporation

the tigrina complex using genome data.
Trindade, F. J., Rodrigues, M. R., Figueiró, H. V., Li, G., Murphy, W. J., & Eizirik, E. (2021). Genome-wide SNPs clarify a complex radiation and support recognition of an additional cat species. Molecular Biology and Evolution, 38(11), 4987-4991.
R/Thanks for the suggestion, we added the reference.

- Line 73: may be interesting to cite the IUCN reference too
R/Added.

- Lines 82-83: Be careful with this comparison. About which Brazilian species you are talking (L. tigrinus or L. guttulus)? I suggest remove the comparison and just talk about the species in Colombia.
R/Agree and changed.

- Line 105: Add a comma after South America
R/Added.

- Line 123: Exclude “for”
R/Corrected.

Methods
- Lines 126-135: I think it is important to define, for each attribute, what was considered high, medium and low. For example, records from human observation were included in the final dataset? This information may be added as a table in the supplementary information, or at least refer to the Table S1 in this part of the methods.
R/Thanks, we added details and the table.

- Lines 143-146: What about when the points were only in the border of an ecoregion, in a transition zone, this ecoregion was also used for the calibration? If so, wouldn’t this bias the result, overfitting the data to the ecoregions that have well balanced points across their area? It could be useful to have a map with the ecoregions and the presence records in the supporting information.
R/Thanks for the comment, we added some details in methods and results, and also added the map in SI.

- Line 157: Change “base” to “based”
R/Corrected.

- Lines: 184-187: Please provide a citation for this information.
R/Added.

- Lines 187-189: Authors say they selected 9km as the maximum dispersal threshold based on previous studies, but reading the references cited to this information, I was not able to find any information about the dispersal ability of the species (however, I was not able to find Zarrate-Charry dissertation). These papers talk about population density, home range size and movement within home range, but not dispersal ability. I think that the lack of knowledge on the dispersal ability of the species should be stated more clearly.

- Also, authors used a 9km dispersal distance for the connectivity analysis. However, they considered the home range of the species to be 17km2 and the dispersal ability of a species is usually bigger than the home range (see Bowman et al. 2002). Therefore, although the real dispersal distance of the species may not be far from 9km, test a different value (a bigger one) and compare the results could be very useful, as it may change the connectivity between patches.
R/Thanks for the comment and we totally agree it was confusing. We provided extra details on why we used different figures trying to be as conservative as possible given the inherent uncertainty from using ecological attributes from a different species. Details were provided both on the selection of core habitats and the connectivity analyses. We expect the details are now clearer specifically on how we made the methodological decisions.

Indeed, the studies we found in our review did not present specific information regarding dispersal distance. The studies that we found were mainly directed to

understanding the home range of the species. We used such information to calculate a proxy for its dispersal distance based on the proposal of Bowman et al. 2002. To define the home range to calculate the median dispersal distance we used as threshold 9 km, which represents a dispersal distance of an individual with a home range of 4.5 km 2 , which represent the median value of all reported home ranges for the species. We used a median value based on the distribution of the existing values. We maintained a mean value for the dispersal distance to reduce potential number of corridors of length that exceed its use within conservation planning strategies. The identification of corridors using circuit theory provides landscape solutions that require efforts to become functional in the territories.

We did not used within our analysis a dispersal distance based on the same home range used to define minimum core habitat patch size. We used different values to create the spatial representation. To define core habitat patches we used the maximum home range reported for the species (17km 2). We use this threshold as a prioritization measure, to ensure that the remaining patches can have at least one individual. We did not used a conservative approach and maintain patches smaller to 17km 2 , due to the inherent uncertainty of such patches to maintain at least one individual. The higher value encloses for sure all the existing reported home ranges.

- Line 197: remove “a” on “or a the most”
R/Corrected.

- Lines 208-210: Has this method been used before?
- How was the scale (1-100) determined?
- The transformation of the human footprint into resistance layer was performed using a linear transformation? Please add this information.
R/We added details and a citation.

- Line 224: change “estimate” to “estimated”
R/Corrected.

Results
- Results and discussion are a little bit repetitive. I believe it would increase the quality of the paper if they were reviewed.
R/We reviewed ancd changed what was possible after following all the rest of the comments. Although we appreciate the comment, we believe the Results section is entirely focused on presenting the results while we try to focus on the conservation aspect on the discussion.

- Line 270: change “has” for “had”
R/Changed.

- Line 273: - Line 270: change “reduces” for “reduced”
R/Changed

- Figure 1 requires more explanation, as there are aspects in the figure that are no specified in the legends. Specifically, it’s important to clarify that the background is an elevation raster, that the yellow lines demonstrate Colombian boundaries and what gray lines are (rivers maybe?). Otherwise, these elements should be removed from the map.
R/Thanks for the comments we modified the figure according to PLOS policies and added details to the caption.

- Lines 282-286 and Figure 2: In this part, authors talk about the small amount of big areas, what can be seen in Figure 2B. However, looking at Figure 2A, we see a great amount of areas in blue colors, indicating their big size. In fact, looking to the map, the amount of area in blue seems to be almost as big as the amount of area in red/brown (the smallest areas). Of curse big patches will occur in smaller numbers than small patches, as they occupy a bigger area, so I think it’s fair to add another graph showing the total amount of area that the different patches sizes occupy, and also talk about this in the text.
R/Thanks for the comment but we believe the reviewer was confused. First, there is only 1 patch in blue which covers over 35000 km, thus is not a lot. Nevertheless, we

Powered by Editorial Manager® and ProduXion Manager® from Aries Systems Corporation

	changed the colors to allow easier reading and we believe the graph and text already clarifies that only 1 patch is really big, the rest are small. Furthermore, the text includes the total area available, the distribution of frequencies and how many patches cover how much of the available habitats. The figure suggested is precisely the figure included in Fig2B (the size of the different patches); in fact, it is not clear what graph could show the amount of area occupied by different patch size (same variable). Nevertheless, we change the map and added some small details in the text.<br><br>- Line 284: change "however" for "thus"<br>R/Followed suggestion by Rev1.<br><br>- Table 1: The estimation categories presented are a little confused. It is hard to understand how "high" and "fragments" can be under the same criteria. I think a more elaborated explanation is required for this column.<br>R/Thanks for pointing that out. We added an explanation in the methods section.<br><br>- Figure 5: The caption and the legends of both figures are not very clear. It could be better elaborated.<br>R/Thanks, we reworded both.<br><br>- Figure 6: change "total proportion protected areas across..." for "total proportion of protected areas across…"<br>R/Changed.<br><br>Discussion<br>- Line 400: citation - Add Trindade et al. (2021)<br>R/Added.<br><br>- Line 403: "lowlands of Brazil" - Again, I would remove this state or change the language to something like "lower elevation areas than the Andes"<br>R/Eliminated by suggestion of the other reviewer.<br><br>- Lines 424-425: Authors state that on the most optimistic scenario, they estimated that there are less than 10,000 individuals. However, on table 1, of the 5 scenarios, 3 estimate more than 10,000 individuals and 1 scenario estimate almost this number (9,000 individuals). It should be less than 10,000 on the LESS optimistic scenario, no?<br>- Still, according to the IUCN, most of these estimations are bigger than the estimated number of L. guttulus for its entire range, and both L. guttulus and L. tigrinus are listed as vulnerable – just a comparison.<br>R/Agree, we reworded accordingly and even added the proper comparison with L. guttulus.<br><br>- Line 479: remove "a" cities<br>R/Removed.
**Additional Information:**	
**Question**	**Response**
**Financial Disclosure**<br><br>Enter a financial disclosure statement that describes the sources of funding for the work included in this submission. Review the submission guidelines for detailed requirements. View published research articles from *PLOS ONE* for specific examples.<br><br>This statement is required for submission and **will appear in the published article** if the submission is accepted. Please make	This project was jointly funded by ProCAT Colombia and Grupo de Energía de Bogotá S.A E.S.P through mutual agreement. The funders had no role in study design, data collection and analysis, decision to publish, or preparation of the manuscript.

sure it is accurate.<br><br>**Unfunded studies**<br>Enter: *The author(s) received no specific funding for this work.*<br><br>**Funded studies**<br>Enter a statement with the following details:<br>• Initials of the authors who received each award<br>• Grant numbers awarded to each author<br>• The full name of each funder<br>• URL of each funder website<br>• Did the sponsors or funders play any role in the study design, data collection and analysis, decision to publish, or preparation of the manuscript?<br>• **NO** - Include this sentence at the end of your statement: *The funders had no role in study design, data collection and analysis, decision to publish, or preparation of the manuscript.*<br>• **YES** - Specify the role(s) played.<br><br>* typeset	
**Competing Interests**<br><br>Use the instructions below to enter a competing interest statement for this submission. On behalf of all authors, disclose any competing interests that could be perceived to bias this work—acknowledging all financial support and any other relevant financial or non-financial competing interests.<br><br>This statement is required for submission and **will appear in the published article** if the submission is accepted. Please make sure it is accurate and that any funding sources listed in your Funding Information later in the submission form are also declared in your Financial Disclosure statement.<br><br>View published research articles from *PLOS ONE* for specific examples.	The authors have declared that no competing interests exist.

Powered by Editorial Manager® and ProduXion Manager® from Aries Systems Corporation

**NO authors have competing interests**<br><br>Enter: *The authors have declared that no competing interests exist.*<br><br>**Authors with competing interests**<br><br>Enter competing interest details beginning with this statement:<br><br>*I have read the journal's policy and the authors of this manuscript have the following competing interests: [insert competing interests here]*<br><br>* typeset	
**Ethics Statement**<br><br>Enter an ethics statement for this submission. This statement is required if the study involved:<br><br>• Human participants<br>• Human specimens or tissue<br>• Vertebrate animals or cephalopods<br>• Vertebrate embryos or tissues<br>• Field research<br><br>Write "N/A" if the submission does not require an ethics statement.<br><br>General guidance is provided below. Consult the submission guidelines for detailed instructions. **Make sure that all information entered here is included in the Methods section of the manuscript.**	N/A

Powered by Editorial Manager® and ProduXion Manager® from Aries Systems Corporation

**Format for specific study types**<br><br>**Human Subject Research (involving human participants and/or tissue)**<br>• Give the name of the institutional review board or ethics committee that approved the study<br>• Include the approval number and/or a statement indicating approval of this research<br>• Indicate the form of consent obtained (written/oral) or the reason that consent was not obtained (e.g. the data were analyzed anonymously)<br><br>**Animal Research (involving vertebrate animals, embryos or tissues)**<br>• Provide the name of the Institutional Animal Care and Use Committee (IACUC) or other relevant ethics board that reviewed the study protocol, and indicate whether they approved this research or granted a formal waiver of ethical approval<br>• Include an approval number if one was obtained<br>• If the study involved *non-human primates*, add *additional details* about animal welfare and steps taken to ameliorate suffering<br>• If anesthesia, euthanasia, or any kind of animal sacrifice is part of the study, include briefly which substances and/or methods were applied<br><br>**Field Research**<br><br>Include the following details if this study involves the collection of plant, animal, or other materials from a natural setting:<br>• Field permit number<br>• Name of the institution or relevant body that granted permission	
**Data Availability**<br><br>Authors are required to make all data underlying the findings described fully available, without restriction, and from the time of publication. PLOS allows rare exceptions to address legal and ethical concerns. See the PLOS Data Policy and FAQ for detailed information.	Yes - all data are fully available without restriction

A Data Availability Statement describing where the data can be found is required at submission. Your answers to this question constitute the Data Availability Statement and **will be published in the article**, if accepted.<br><br>**Important:** Stating 'data available on request from the author' is not sufficient. If your data are only available upon request, select 'No' for the first question and explain your exceptional situation in the text box.<br><br>Do the authors confirm that all data underlying the findings described in their manuscript are fully available without restriction?	
**Describe where the data may be found in full sentences. If you are copying our sample text, replace any instances of XXX with the appropriate details.**<br><br>• If the data are **held or will be held in a public repository**, include URLs, accession numbers or DOIs. If this information will only be available after acceptance, indicate this by ticking the box below. For example: *All XXX files are available from the XXX database (accession number(s) XXX, XXX.).*<br>• If the data are all contained **within the manuscript and/or Supporting Information files**, enter the following: *All relevant data are within the manuscript and its Supporting Information files.*<br>• If neither of these applies but you are able to provide **details of access elsewhere**, with or without limitations, please do so. For example:<br><br>*Data cannot be shared publicly because of [XXX]. Data are available from the XXX Institutional Data Access / Ethics Committee (contact via XXX) for researchers who meet the criteria for access to confidential data.*<br><br>*The data underlying the results presented in the study are available from (include the name of the third party*	Data about the species are within the manuscript and its Supporting Information files. Climatic data is available from WorldClim (https://www.worldclim.org). Protected areas information is available from the Registro Único Nacional de Áreas Protegidas (https://runap.parquesnacionales.gov.co/).

and contact information or URL).<br>• This text is appropriate if the data are owned by a third party and authors do not have permission to share the data.<br><br>* typeset	
Additional data availability information:	

Powered by Editorial Manager® and ProduXion Manager® from Aries Systems Corporation

Manuscript Click here to access/download;Manuscript;Manuscript.docx

*Spot*ting what's important: priority areas, connectivity, and conservation of the Northern Tiger Cat (*Leopardus tigrinus*) in Colombia

Leopardus tigrinus in Colombia

José F. González-Maya[1, 2], Diego A. Zárrate-Charry[1, 3], Andrés Arias-Alzate[4], Leonardo Lemus-Mejía[1], Angela P. Hurtado-Moreno[1], Magda Gissella Vargas-Gómez[5], Teresa Andrea Cárdenas[5], Victor Mallarino[6], Jan Schipper[7]

[1] Proyecto de Conservación de Aguas y Tierras – ProCAT Colombia, Carrera 11 # 96-82, Of. 303, Bogotá, Colombia.

[2] Departamento de Ciencias Ambientales, División de Ciencias Biológicas y de la Salud, Universidad Autónoma Metropolitana Unidad Lerma, Lerma de Villada, C. P. 52005, Estado de México, México.

[3] WWF Colombia, Cra. 10a # 69 A – 44, Bogotá, Colombia.

[4] Facultad de Ciencias y Biotecnología, Universidad CES, Calle 10 A # 22-04, Medellín, Colombia.

[5] Grupo Energía Bogotá S.A. E.S.P., Carrera 9 # 73-44, Bogotá D.C., Colombia.

[6] Independent, Bogotá D.C., Colombia.

[7] Arizona Center for Nature Conservation/Phoenix Zoo, Phoenix, Arizona, United States.

* Corresponding author:

E-mail: jfgonzalezmaya@gmail.com.

Abstract

Leopardus tigrinus is among the least known carnivore species in the Andes, including considerable taxonomic uncertainty. Mostly known from Brazil (currently suggested as two distinct species), the Andean and isolated Central American forms remain among the least understood carnivores in the Neotropics. Here we model the distribution, connectivity and overlap with existing conservation areas for the species in Colombia. Using a Species Distribution Modeling approach, we estimated current potential range of the species in Colombia and identified potential habitat blocks remaining in the country. In addition, we designed a connectivity network across the available cores, using a circuit theory approach, to evaluate habitat linkage. Finally, we defined a prioritization scheme for the remaining habitat cores and assessed the level of coverage of protected areas for the country. *L. tigrinus* is potentially present across the three Andean branches of Colombia, with still considerable continuous habitat cores, mostly located on the eastern and central Andean ranges. Most habitat cores are theoretically connected, but nearly 15% are isolated. Priority areas were located across the eastern and central ranges, but with very significant and promising cores in the northern eastern and western ranges. Current level of protection indicates nearly 30% of the range is “protected”, but only about 25% is under national strict protected areas. Evolution of this coverage showed some periods of significant increase but interestingly the number of cores grew at a faster rate than overall proportion protected, likely indicating numerous discontinuous fragments, and not contiguous functional landscapes. This represents the most updated assessment of the distribution and conservation status for the species in Colombia, and indicates the numerous conservation opportunities, especially in most populated areas of the country. We found unique business

environmental passive's opportunities, including compensation and development potential, which are becoming more available in the country.

Key Words: Species distribution modeling, Oncilla, Tigrina, Connectivity, Andes**.**

Introduction

Effective species conservation requires solid scientific information to support decision- and policy-making [1]. Basic information such as distribution, abundance and threats are among the most important aspects needed for effective conservation planning [2, 3], especially when such a large number of species are under threat [4]. Despite their charismatic nature, important role in ecosystem balance and general ecological importance, most carnivores lack even the most basic information [5-7], undermining effective conservation actions, especially given their particular sensitivity to the most pressing threats [8].

Despite the fact that massive efforts have concentrated on a handful of “mega-charismatic” carnivore species worldwide [6], small carnivores have seemingly been overlooked [5, 9]. In fact, some small carnivore species are considered among the least known among mammalian fauna in many countries [5, 10]. Even for wild felids, one of the groups which have received more attention [11], the smaller species are the least understood while at the same time being among the most threatened [11-13].

This is certainly the case for the Northern Tigrina or Northern Tiger Cat, *Leopardus tigrinus*, one of the smallest felids with a wide distribution in the Americas, and perhaps the least understood spotted cat known in the continent [14-16]. Important gaps in information about the species include certainty of its taxonomic status [17-19], as well as even basic knowledge of its distribution, ecology and conservation status [14, 15, 19]; however, significant progress have provided new insights into the group´s taxonomy [16-18]. With a considerably large range, from Costa Rica down to Bolivia and Peru, and East across Venezuela and the Guianas [17], still taxonomic uncertainty does not allow precise distribution assessments, varying according to different authors and taxonomic approaches [16-18]. Mostly unknown, both ecologically and

taxonomically, across its distribution (even with scarce samples), the species has being studied mostly in Brazil [20, 21], where it was even recently split into two distinct species [16, 17]. Information about Northern Tiger Cat for the rest of its range countries is restricted to just occasional records and sporadic observations, but with no systematic data on any other aspects of its ecology and especially conservation status [14, 22-25].

Specifically, in Colombia the species is only known from locality records and observations, with most of the literature focused on distribution and confirmed localities [14, 24, 26], with very few National scale systematic approaches to its distribution and conservation status [14]. Also, in Colombia the species is mostly distributed at higher elevations in the Andes, above 1,500 m asl [14], which is also one of the most transformed and heavily populated regions in the country [27]. Given the considerable pressure over Andean ecosystems in general [28-30], and the specific pressure that such transformation exerts over the species [15], understanding the species distribution and conservation status represents an urgent need as its remaining habitat slowly disappears. Furthermore, given the considerable human development pressure on the remaining habitat for the species, the Northern Tiger Cat has become a predominant species challenging many development and infrastructure projects in the country, necessitating better information that can help informing and plan for its conservation. This could represent a unique conservation opportunity for the species, especially considering the substantial compensation requirements of companies interested in these types of projects. To provide baseline information that can be incorporated in future conservation planning, including compensation and licensing of future development projects, herein we assess the distribution and conservation status of the species in the country. Specifically, i) we assessed the distribution of the species at a national scale, ii) illustrate potential connectivity networks across remnant habitats, iii) selected and

categorized discrete priority remaining habitats, and iv) assessed the conservation status of the priority habitats.

Materials and methods

Study area

Our study focused on the known distribution range of Northern Tiger Cat in Colombia [14], which encompasses the entire terrestrial portion above 2000 m asl of the country. Colombia is located in northwestern South America, bordering with Brazil, Venezuela, Peru, Ecuador and Panama; it is located on the northernmost part of the continent and serves as the only terrestrial limit with Central America. Given the focus of our study, it is worth mentioning that Colombia is also the northernmost part of the Andes where the mountain range splits into three branches (i.e., western, central, and eastern ranges), where they reach elevations over 5000 m. The unique features of the northern Andes represent an interesting case study in terms of evolution and biogeography; the divided mountain range creates unique conditions for a large variety of life-forms with many species ranging on the three branches and some restricted to either of them [31]. Although our study focuses on the higher parts of the Andes, some isolated ranges are also included in the analyses, including the Macarena, Perijá, San Lucas and Sierra Nevada de Santa Marta. These are all independent mountain ranges for the Andes but also reaching elevations where the species is known or thought to occur.

Methods

Species' records

Our species distribution model (SDM) was based on the most updated compilation of confirmed available records for the species, including a thorough

literature review [14, 17, 24, 32-39], and consulting all available national and international databases (i.e., SIB Colombia, GBIF, VertNet [40, 41]) covering a temporal range between 1951 and 2019.

Once a final databased was compiled, we discarded all records without specific locality information or coordinates and then used three criteria filtering for data quality control – reducing the available data to only confirmed locations (Table S1). We generated a categorization scheme for each record based on the reliability of the source, type of evidence and geographic precision. We classified each record according to the source, the supporting evidence and geographic precision; precision was evaluated by comparing the coincidence between locality details reported in the source and the national cartography of Colombia (Table 1; [42]). Each attribute was classified in three categories (i.e., high, medium, low) that were latter cross referenced for each record (Table 1). We then applied a moderate filter, in which any record with one category ranked as low would be excluded from the final model [43, 44]; we considered this filter as moderate given that we did not use only those categorized as high in the three attributes as for previous efforts [43-45]. Finally, we eliminated duplicates and used a spatial filter which eliminated any redundant records on a 1 km^2 grid [46].

Table 1. Criteria used for classifying three reliability criteria of records for modeling the potential distribution of *L. tigrinus* in Colombia.

Category	Evidence	Reliability
Evidence	Preserved specimens	High
	Machine observation/Photographic record	High
	Material sample	Medium
	Human observation	Low
	No data	Low
Source	Peer reviewed article	High
	Expert validated record	High
	Museum	Medium
	GBIF	Medium
	Technical information	Low
Geographic	Department and Municipality	High
	Department	Medium
	Municipality	Medium
	None	Low

Distribution modelling and potential habitats

For the assessment of the species potential distribution, we generated a SDM based on an ecological niche modeling approach [47] using the maximum entropy algorithm [48]. To define the calibration area of the SDM we obtained an updated polygon layer of the world´s ecoregions [49] as the Mobility area (M) for the species [50] and selected those ecoregions that had at least one record of the species after filtering and we narrowed-down all the inferences to the extent of those ecoregions [51]; all ecoregions selected correspond to those ecoregions associated with the high Andean ecosystems (Figure SI2). We then obtained bioclimatic variables from Worldclim 1.4 [46], including those that represented best the variation for a species such as Northern Tiger Cat, and that have proved useful for modeling carnivore distributions [52, 53]. Specifically, we used seven bioclimatic variables: Mean Annual Temperature (Bio1), Diurnal Mean range (Bio2), Temperature seasonality (Bio4), Annual precipitation (Bio12) and Precipitation seasonality (Bio14), Precipitation of the wettest month (Bio13) and Precipitation of the driest month (Bio14). These last two variables have shown a better representation of the ENSO (El Niño-Southern Oscillation) effects [54]. Finally, we included elevation [42], given its potential influence on species distribution models, especially for the Andean region [51]. We then analyzed potential correlation between these variables across our calibration areas using a Spearman correlation ranks test and the jackknife test based on the maximum entropy model to select those with correlation scores under 70% and the highest permutation values [43].

We used a maximum entropy approach available through the software Wallace 1.0.6.2 [55], which include different R-based packages, including Maxent [56] and ENMeval [57]. Maximum entropy analysis is the most widely used algorithm for quantifying the relationship between the presence of a species and environmental

variables [58, 59]. The records database was divided into calibration and testing dataset following Cobos et al. [60]. For each set of variables, we created candidate models (based on 10 replicates by bootstrapping and selecting the logistic output) using the calibration occurrences, 17 different Regularization Multiplier (RM) values (0.1 – 0.9 at intervals of 0.1, 1 – 6 at intervals of one, and 8, and 10), 29 possible Feature Class (FC) combinations of five feature classes (linear = l, quadratic = q, product = p, threshold = t, and hinge = h), with 10,000 background points. These candidate models were evaluated and selected using the testing dataset, based on statistical significance (Partial ROC, with 500 iterations and 50% data for bootstrapping), omission rate (OR ≤5%), and model complexity with the Akaike Information Criterion corrected for small sample sizes (AICc) [60]. The resulting model was then transformed into binary (i.e., presence-absence), following a conservative threshold, using the 10 percentile training presence logistic threshold value [61], averaged across the 10 replicates.

Selection of available suitable habitats

Once a binary map was obtained from the best model, we evaluated the different types of vegetation cover included in the suitable areas according to available information on species' use [26, 44]. All coverage catalogued as natural by the national cartography dataset [62] and with existing evidence of use by the species from literature and the distribution records, was selected within the binary map [44]. Once all coverages were extracted, we defined an area threshold for selecting those remaining patches that could be used by the species as core habitats, based on a conservative value estimated from the maximum home range reported for the species of 17 km^2. The selection of such threshold aims at prioritizing those core habitat patches that ensure viability of at least one individual considering the inherent uncertainty of the home

range size for the species and the base- data obtained from a different but closely related species (reported values range from 0.9 to 17,1 km^2; [63]).

Connectivity networks definition

Once all available remaining habitats were identified, we designed an ecological connectivity network analysis across the recognized patches based on a circuit theory approach [64, 65]. The ecological connectivity network is based on the resistance that certain landscape features exert over the potential dispersibility of a given species between its core habitats [64, 65]. We consider as core habitats the recognized habitat patches remaining within the suitable areas identified in the species' distribution model. The resistance layer was generated based on the available "human footprint index" developed for Colombia [66]. This human footprint approach includes land uses, rural population density, distance to human settlements and roads, a general fragmentation index, a comparative biomass index compared to the original, and time of intervention [66]. These variables represent multiple levels of human influence over landscapes, including current land use, which can be also used as a proxy to potential barriers (i.e., resistance) to movement across fragmented landscapes [66]. Our assumption is that without knowing the species-specific resistance values that multiple interacting variables can exert over species dispersal, a standardized and weighted human-influence index can validly reflect the resistance of such influence over species movement (especially on highly intervened landscapes such as the Colombian Andean region) [67]. We then used the 2015 Human spatial footprint for Colombia, comparable to our coverage layer for 2016 [62] and used a linear transformation to turn the footprint into a resistance layer by rescaling it to a 1-100 standard scale [68]. The connectivity network was then confined to a 15 km dispersal distance in order to identify corridors that pertain to the species potential movements and thus avoiding overestimations [44]. This

value represents a dispersal distance of an individual with a home range of 4.5 km^2, which represent the median value of all reported home ranges for the species [20, 44, 63]. This method has been previously used for similar purposes in Colombia and the region [33, 43, 69-71]. We then identified the importance of each core habitat by estimating its centrality value (CF_Central value) and the potential current flow for each link within the connectivity network; centrality represents a measure of how important is a linkage for maintaining the overall connectivity network connected, while current flow helps predicting the expected net movement probability of an individual though the linkage [65, 72-74]. We tested the relationship of core habitat size with centrality value using a linear regression and decremental regression analyses (i.e., chained regressions) excluding cores over different size thresholds. We used the adjusted R^2 values to find the threshold where the relationship significantly decreases. Our connectivity network was developed using the Linkage Mapper 10.x toolbox [72, 73] available for ArcGIS [75].

Selection and categorization of priority areas

Once a network of core suitable habitats and a connectivity network was established, we defined discrete units for each core and estimated the following parameters to stablish a priority scheme for Northern Tiger Cat in Colombia. We defined three attributes of the classification criteria that allowed a weighted ranking of all core areas to define a prioritization scheme according to: i) Size, ii) Mean human footprint and iii) Centrality value. Size was estimated as the total area of each discrete polygon identified as core habitat (log-transformed), mean human footprint as the mean value of the 2015 human footprint overlapping the polygon [66] and centrality (log-transformed) as estimated by the circuit analyses [73]. We then summed up the three categories and estimated a final value for each core which was then qualified,

categorized, and standardized generating an ordinal priority from 1 to 5, from the definition of an equal interval of the total range of values, identifying those cores with the best existing conditions (1) to those severely fragmented or affected by human intervention (5).

Conservation measures for Northern Tiger Cat populations in Colombia

Finally, for each core area we estimated several species conservation measures including two parameters and our prioritization scheme. Parameters used are i) number of individuals potentially present on each core and ii) coverage of protected areas. The number of individuals was estimated by extrapolation from known density estimations found elsewhere [15] to the available core habitat areas identified through our models. Density estimations are only available for Brazil [15, 20, 76, 77], so we used all these reports to calculate the minimum number of individuals and the potential variation between estimations; we estimated upper and lower limits and, mean estimation from different sources and distribution of the total number of individuals expected. Specifically, we used complementary estimations, including those considered high, typical and those previously reported for fragmented landscapes according to previous authors [15, 76], and our mean estimate from the existing information. It is necessary to acknowledge the potential bias of our approach given that no specific information exists for the species and for Colombia, and that likely these estimates correspond to *L. guttulus*; nevertheless, given this is the best available information, we believe this approach can be informative until better and more specific information becomes available

For assessing the coverage of protected areas we estimated the overlapping of the remaining core habitats with the national official protected areas layer [78]. First, we

estimated the proportion of core areas covered by all protected areas from the national, regional, and local levels reporting the total level of protection over the entire SDM and for each core. We then estimated the proportion of cores completely or partially protected and according to the protection type. Finally, we assessed how the coverage of protected areas have changed over time to understand how such protection has evolved in terms of total covered area and number of cores fragments included.

All maps were created using ArcGIS [75] and we used a global Digital Elevation Model [79] as background for all map figures.

Results

Distribution of Northern Tiger Cat in Colombia

We obtained a total of 448 records of the species, of which 212 were considered of high quality and credibility (S1 Table). Our sampling area included 6 ecoregions with previously confirmed presence of the Northern Tiger Cat: Cauca Valley montane forests, Cordillera Oriental montane forests, Eastern Cordillera Real montane forests, Magdalena Valley montane forests, Northern Andean paramo and Northwest Andean montane forests (S2 Figure). Derived from the correlation tests, we eliminated from further analyses Annual Mean Temperature, Precipitation of the Wettest Month and Precipitation Seasonality, which were highly correlated to elevation (Spearman= -0.90), Annual Precipitation (Spearman= 0.77) and Precipitation of the Driest Month (Spearman= -0.94), respectively (S3 Table). Among the most important predicting variables, elevation (64.3%) and temperature seasonality (27.9%), accounting as the most important according to permutation importance, but with Annual Precipitation (6.3%), Precipitation of Driest Month (0.94%) and Mean Diurnal Range (0.45%), also influencing its pattern. As expected, elevation had a positive influence on the species´

model (higher probabilities at higher elevations), together with Annual Precipitation, while Temperature Seasonality, Precipitation of the Driest Month and Mean Diurnal Range showed predominantly negative influence (higher variable values, lower probabilities), although with some interesting changes (S4 Figure). The most informative and best selected models were the LQHP (Linear Quadratic Hinge Product) feature class and the 2.5 and 2.0 regularization multipliers models (AICc: 4323.54 and 4323.61, respectively), both models indicate good performance with AUC of 0.8902 and 0.8923, respectively (S5 Table). The resulting 10 percentile training presence logistic threshold binary map, now on SDM, had a total of 228817.43 km^2 of environmentally suitable areas distributed in the three branches of the Andes in Colombia (Fig 1A). However, given the large-scale human-caused natural cover transformation processes across the distribution of the species, our selection of suitable remaining habitats significantly reduced these available areas for the species (Fig 1B).

Figure 1. Distribution of (A) total records obtained for *Leopardus tigrinus* and potential distribution (SDM) in Colombia and (B) remaining habitat cores within the potential distribution (SDM). Background shows topography [79], light gray depicts Colombia and white lines define the political and administrative divisions of Colombia (departments). Map created using ArcGIS [75].

After identifying the available natural coverages within these suitable areas, we found the potentially occupied areas to be only 91,209.85 km^2 (~39.8% of the SDM), distributed in 183 patches, with a mean (±SD) area of 498.41±2783.58 km^2 across the SDM (Fig 2A). This area corresponds to less than 8% of the country area and nearly 40% of the suitable area. We found significant variation among fragments, with many patches covering barely the 17 km^2 threshold (155 (84.7%) under 20 km^2), with 93% under 1,000 km^2 and only one remaining patch covering over 35,000 km^2 (Fig 2B). Most remaining habitat patches were concentrated towards the smaller size, with very few close to the largest one (Fig 2B); although, only 12 patches (all over 100 km^2) cover

together over 76,000 km^2, representing over 83% of the species distribution. The largest remaining habitats are located on the southern central and eastern mountain ranges, including the "macizo colombiano", where both ranges divide, followed by the northern portion of the eastern range (Fig 2A).

Figure 2. (A) Size of available habitat cores for *Leopardus tigrinus* in Colombia and (B) distribution of fragments according to size classes. Background shows topography [79]; map created using ArcGIS [75].

Potential connectivity network for Leopardus tigrinus in Colombia

Our ecological network analyses selected those areas with the lowest cost for dispersal between habitat cores according to the lowest cost weighted distance between each core. The resulting network, pruned to the maximum 9 km threshold dispersal value, identified approximately 250 links between habitat cores, varying in terms of Euclidean and length distance variation and potential current flow (*sensu* McRae, Dickson (73)). Corridor links mean (±SD) Euclidean and length distances were 2.52±2.45 and 3.31±2.97 km, respectively, while potential current flow was 504.42±650.66 (Fig 3A). Interestingly, 156 of the total 183 had at least one linkage to other cores and the mean (±SD) number of links between cores was 1.61±0.80, with the largest number reaching 5 for a single core.

Centrality values for all cores also varied considerably, with a mean value of 981.53±1128.85 for the entire network, but with an interesting geographical variation (Fig 3A); overall centrality value was closely related with the size of the core (T=16.40, $p<0.001$, R^2Adj=0.60; Fig 3B), indicating both a geographic (location) and spatial influence on the entire network. However, the centrality value variations explained by area (Adjusted R^2) tended to decrease towards the smaller cores, indicating significant importance of the largest ones. Additionally, there is a peak for all cores below 2,000

km^2 (T=11.17, p<0.001, R^2Adj=0.42), which rapidly decrease until only cores below 100 km^2 are included, where the influence is no longer significant (T=1.09, p=0.2782, R^2Adj=0.013; Fig 3B).

Figure 3. Distribution of (A) potential connectivity corridors and centrality values across remaining habitat cores for *L. tigrinus* in Colombia and (B) decremental variation of connectivity importance explained by core area size. Background shows topography [79]; map created using ArcGIS [75].

Priority areas for the conservation of Northern Tiger Cat in Colombia

Our approach for priority areas selection identified those cores that represent the best opportunities for conservation given their available area, their contribution to connectivity and, ideally, where human influence is lower. Mean (±SD) area for all cores was 498.41±2791.22 km^2, but with a concentration towards the smallest patches, as mentioned above. Mean (±SD) value for centrality, importance for the maintenance of the connectivity network, was 981.53±1131.96, where the largest patches were also coincident with those that contribute more significantly to overall connectivity (Fig 3A). In terms of human influence, mean (±SD) index values for all cores was 35.20±19.53, also showing that the largest cores remain with lower human influence (Fig 4B).

After weighting the three criteria, we generated an ordinal priority scheme in five categories (Fig 4A), indicating those currently considered to be strongholds for the species conservation. The main core areas that currently retain ideal conditions for the species, those in Category 1 thus the most preserved, are in the central and eastern ranges of the Andes, concentrated towards the largest, less disturbed, continuous habitat cores in Colombia. However, several opportunities arise for the following two categories, in which important relicts are still present and under appropriate conservation, and those where potentially restoration schemes could significantly

contribute to the maintenance of the species´ populations (Fig 4A). Again, there is a strong relationship between the largest areas with the largest contribution to connectivity and lower levels of human influence (Fig 4B).

Figure 4. (A) Prioritization of remaining habitat cores for *L. tigrinus* in Colombia and (B) relationship between human influence (X-axis), connectivity importance (centrality; Y-axis) and core size (bubble size) of all habitat cores. Background shows topography [79]; map created using ArcGIS [75].

Conservation measures for *Leopardus tigrinus* populations in Colombia

Our estimation of mean number of individuals, based on previous calculations for likely a closely related species, *Leopardus guttulus,* highlight the significant composition of small core habitats, harboring potentially very few individuals. Mean density estimates for the four ranges obtained from previous studies indicated a mean (±SD) density of 0.114±0.051 individuals/km^2. The large variation between previous estimates indicates a potentially large variation in the number of individuals present on each core habitat across the country and thus for the country total (Table 2). Accordingly, the number of individual Northern Tiger Cat estimated for Colombia, according to the areas with potential occurrence identified by our model, is 10,375±4674 individuals, but with significant variation (Table 2). Only 12 core habitats (above 1000 km^2) contribute over 83% of the total national population (Fig 5A) and almost 95% of the identified potentially cores harbor under 100 individuals (Fig 5B).

Table 2. Population density estimation and potential population size, based on previous estimations, in core habitats within the potential distribution of *L. tigrinus* in Colombia.

Type of estimation	Range	Mean estimated population	±SD	Source
Typical density	0.01-0.05	2736	1824	[15]
High density	0.1-0.25	15962	6841	[15]
	0.15±0.08	13681	7297	[77]
Fragmented landscape*	0.07-0.13	9121	2736	[15]
Mean estimate	0.114±0.051	10375	4674	Our estimation

Figure 5. (A) Mean (blue line), maximum and minimum (dotted lines), number of potential individuals per core habitat organized by increasing size, and cumulative potential population size (orange line) of *L. tigrinus* present at available habitat cores in Colombia and (B) frequency histogram showing the distribution of the number of core habitats according to the potential population size of *L. tigrinus* in Colombia.

Interestingly, in terms of protection level, we found that the species is estimated to be present in at least 415 protected areas from the national to the local level, representing about 33,270.68 km^2 (about 36.47% of the total range) (Fig 6A). The majority, albeit with very low coverage (114.62 km^2), are represented by Civil Society Natural Reserves (Reservas Naturales de la Sociedad Civil), followed by regional protected areas (Regional Integrated Management Districts - Distritos Regionales de Manejo Integrado; Table 3). National Parks (Parques Nacionales Naturales) were the category with the highest proportion of range covered, however, Unique Natural Areas (Área Natural Única) were the ones that covered the least area (2.98 km^2) – which is represented in a single protected area (ANU Los Estoraques; Table 3). It is important to highlight that only 23.71% of the species distribution is covered by dedicated public lands, with mostly strict conservation, while 12.76 % is under private lands where the mixed uses is allowed (Table 3). Furthermore, 25.87% of the range is protected by national level protected areas, 10.47% under regional protected areas and only 0.1% under local protected areas.

Table 3. Total area and number of protected areas according to level and category currently covering the potential distribution range of *L. tigrinus* in Colombia.

Level	Category (Original in Spanish)	Protection level	Lands Regime	Area (km^2)	Number of areas
National	Fauna and Flora Sanctuary (Santuario de Fauna y Flora)	Dedicated	Public	231.17	4
	Flora Sanctuary (Santuario de Flora)	Dedicated	Public	100.86	2
	National Forest Protection Reserve (Reservas Forestales Protectoras Nacionales)	Mixed	Private	1974.69	39
	National Park (Parque Nacional Natural)	Dedicated	Public	21290.29	22
	Natural Unique Area (Area Natural Unica)	Dedicated	Public	2.98	1
Regional	Regional Forest Protection Reserve	Mixed	Private	1126.98	52

	(Reservas Forestales Protectoras Regionales)				
	Regional Integrated Management District (Distritos Regionales de Manejo Integrado)	Mixed	Private	3169.81	53
	Regional Natural Park (Parques Naturales Regionales)	Mixed	Private	5022.62	37
	Soil Conservation District (Distritos de Conservacion de Suelos)	Mixed	Private	236.63	6
Local	Civil Society Natural Reserves (Reserva Natural de la Sociedad Civil)	Mixed	Private	114.63	199
**Total**				**33270.68**	**415**

Of the 183 core habitats, only 92 (50.27%) currently have any level of protection (Fig 6B). Furthermore, mean (±SD) level of protection across cores currently reach 0.51±0.35, while the mean number of protected areas covering a core reached 9.18±28.94. Over time, coverage of remaining core areas has increased significantly since 1938, with notable increases, in terms of number of cores (or fragments of cores) and area, for 1976, 2008 and 2018 (Fig 6C).

Figure 6. (A) Geographic distribution of protected areas currently protecting the potential range of *L. tigrinus* in Colombia, (B) total proportion of protected areas across available habitat cores and (C) temporal evolution of range (line) and habitat cores (bars) protection in Colombia. Background shows topography [79]; map created using ArcGIS [75].

Finally, it is important to highlight that only seven protected areas, primarily from the national level (National Parks), but also one from the regional level (Regional Natural Park), account for almost half (16.76%) of the total range protected. These areas include: El Cocuy (2917.92 km^2), Paramillo (2806.59 km^2), Sumapaz (2144.15 km^2), Cordillera de los Picachos (1918.87 km^2), Farallones de Cali (1658.95 km^2), Nevado del Huila (1586.69 km^2), and Las Hermosas-Gloria Valencia de Castaño (1192.72 km^2) National Parks and Miraflores Picachos Regional Natural Park (1064.28 km^2).

Discussion

Leopardus tigrinus is among the least known carnivore species in Colombia [14, 26, 80] and even across its distribution its taxonomic status is tenuous at best [16-18].

Considering its presumed widespread distribution, and the long-standing uncertainty regarding its taxonomic position, and given that the form present in the Andes and Central America was until recently considered a different species from *L. guttulus* in the south [81, 82], it remains among the most poorly known felids in South America, with knowledge about the species heterogeneously distributed and overall scarce [17-19, 32]. Despite the recent increased attention to the species and considerable advance in its knowledge and study (e.g., [17]), information about its conservation status is still scarce and mostly unavailable for conservation practices [15, 17, 77].

As previously mentioned, information about the Andean and Central American forms is restricted to sporadic distribution records [14, 22-25, 83, 84], with very few research focused on systematic assessments of its distribution [14, 34] and some limited aspects of its ecology [33, 83, 85, 86]. Such information gaps not only limit the overall knowledge of the species, but also the design of appropriate conservation measures, especially considering that the species is categorized as Vulnerable globally on the IUCN Red List of Threatened Species [19]. Here we present an updated, and systematic assessment of the species distribution for Colombia, with significant considerations relevant for Northern Tiger Cat conservation at the national scale. In addition, our methodological approach can be adopted by other countries to evaluate its current conservation status.

Northern Tiger Cat still retains a widespread distribution at high altitude in Colombia, though largely fragmented and located within the most disturbed areas of the country [67]. Although some very considerable remnants remain, mostly on the Eastern and Central ranges, nearly 93% of its current suitable habitat cores are largely fragmented and isolated, with very small potential populations and under protection. Therefore, based on population estimations and conservation assessments for the

species and likely the recently separated *L. guttulus* in Brazil, for instance [15], could indicate, that the Colombian population of *L. tigrinus* is significantly threatened (i.e., from 2,736 individuals using a "typical" density to almost 16,000 individuals on the most optimistic scenario). Especially considering the distribution of the species on the most populated and transformed area of the country [27, 87]. Nevertheless, and considering uncertainty on the actual densities that the species could have in the country, the species could be potentially more threatened than we currently realize, even reaching the critical numbers proposed for the same *L. guttulus* in the south [88].

It is remarkable however that some large continuous habitats remain across the environmentally suitable areas for the species and represent unique opportunities for designing functional landscapes through complementary conservation measures [89], especially those that consider ecological connectivity on large spatial scales [89-91]. Our prioritization approach identified very considerable continuous habitats towards the eastern and central Andean ranges, but with very promising large, forested areas on the northern portion of the eastern and western branches, where numerous conservation opportunities arise. For instance, the Andean-Orinoco piedmont has been widely recognized as a conservation priority, given its unique biota, and thus, it is likely, and desirable, that it will receive substantial conservation efforts [92-94]. Furthermore, the connectivity network proposed in this work would allow to think on functional connected landscapes especially by articulating marginal fragments to the largest core remaining habitats; this is especially necessary to overcome some of the most significant movement barriers, such as Bogota in the Eastern branch, and reconnecting the northernmost portion of the range in Colombia, and South America.

Current coverage of protected areas across the species distribution probably represents the best opportunity for its conservation. However, several considerations

arise from the distribution across multiple levels and categories, and especially given the very low overall protected proportion (i.e., around 50% of all cores and all under 50% protected). For instance, only a very low proportion of the estimated range is under strict, public, protected areas, while a significant proportion is under private lands and with very low or absent active management and protection [95-97]. Furthermore, a significant number of cores are represented on very small areas, mostly private reserves (i.e., Reservas de la Sociedad Civil), that represent a very important conservation strategy [87, 98], which by themselves contribute very little to the overall population conservation goals, but act as important stepping stones between main habitat core; such role heavily depends on restoring connectivity across our proposed corridors´ network. Additionally, we highlight the relevance of seven national protected areas (i.e., El Cocuy, Paramillo, Sumapaz, Cordillera de los Picachos, Farallones de Cali, Nevado del Huila and Las Hermosas - Gloria Valencia de Castaño National Parks) and one recently created regional area (i.e., Miraflores Picachos Regional Natural Park), which represent the most important strongholds for the species. The effective maintenance and management of these areas represent a unique opportunity for the species conservation in the long term [67, 97, 99].

It is important to highlight the exploration of the temporal protected areas evolution covering the current species range. The creation of new protected areas during the 1970s and 2000s, and even with the recent expansion of regional and local level areas, significantly increased the coverage, adding new areas relevant for the species. However, the proportion of area covered is not the most effective metric through time, because the number of fragments seems to growth at a faster rate, revealing a trend that needs further refined analyses. This also to better understand how new conservation figures can better contribute to conserving the species either by adding continuous

habitats or instead, by increasing the number of smaller fragments protected and connected, likely where functionally and genetically isolated population could survive. Nevertheless, at the current pace of creation of new regional and local protected areas it is likely that total coverage for the species will steadily grow benefiting the overall level of protection, but probably requiring complementary conservation measures that warrant functional landscapes; our connectivity network proposal, although can rapidly change according to land cover changes, it probably represent a critical aspect for the functionality and real contribution of this increasing number of protected, yet probably isolated, fragments. Other effective area-based conservation measures (OECMs) articulated with the existing and increasing protected areas can ensure a functional landscapes, both protected and connected, for this species at the long term [100].

Our effort, although with the already known limitations and uncertainty related to SDMs [47, 101, 102], provides a meaningful basic information that could inform and support decision making. The species distribution located in one of the most populated and transformed areas of the country [27, 30, 67] poses an especially complex situation not only for the species conservation, but in general for the Andean biodiversity protection [28, 67], particularly in Colombia [91]. However, recently the species have received special attention given its prominence in the last remaining peri-urban areas on the capital city Bogota [32, 33, 38] and the Aburrá Valley in Antioquia [[103]]. This unique opportunity has drawn attention for the species, especially in the context of cities that demands more resources and territories for supporting an increasing growing population [104, 105]. For instance, the distribution of the species, although based on very limited assessments [32], has being recently used as a conservation spatial determinant for development and infrastructure projects in the region; thus, providing this information represents a unique opportunity to support and safeguard the last

remaining habitats available for the species. Most importantly, it can represent an incredible opportunity for the investment of compensation funds derived from environmental liabilities of development projects; projects that can support and strengthen different conservation strategies within diverse territories by supporting actions within other effective area-based conservation measures.

Here we present not only an updated distribution assessment for one charismatic and poorly known representative species of the Andes, but also several important arguments to support multiple conservation opportunities in Colombia. Although this assessment warrants its validation based on field efforts, it represents a necessary first step for a unique opportunity to appropriately invest the development resources on safeguarding the last remaining Andean habitats in Colombia.

Acknowledgements

This study is part of the ongoing conservation and compensation efforts of Grupo de Energía de Bogotá and ProCAT Colombia for the species in Colombia. Thanks to the staff at GEB and ProCAT for their support throughout the project.

References

1. Schonewald-Cox C, Azari R, Blume S. Scale, variable density, and conservation planning for mammalian carnivores. Conservation Biology. 1991;5(4):491-5. doi: 10.1111/j.1523-1739.1991.tb00356.x.

2. Sanderson EW, Redford KH, Chetkiewicz C-LB, Medellin RA, Rabinowitz AR, Robinson JG, et al. Planning to Save a Species: the Jaguar as a Model. Conservation Biology. 2002;16(1):58-72. doi: 10.1046/j.1523-1739.2002.00352.x.

3. Rondinini C, Di Marco M, Chiozza F, Santulli G, Baisero D, Visconti P, et al. Global habitat suitability models of terrestrial mammals. Philosophical transactions of

the Royal Society of London Series B, Biological sciences. 2011;366(1578):2633-41. Epub 2011/08/17. doi: 10.1098/rstb.2011.0113. PubMed PMID: 21844042; PubMed Central PMCID: PMCPMC3140734.

4. Schipper J, Chanson JS, Chiozza F, Cox NA, Hoffmann M, Katariya V, et al. The status of the world's land and marine mammals: diversity, threat, and knowledge. Science. 2008;322(5899):225-30. Epub 2008/10/11. doi: 10.1126/science.1165115. PubMed PMID: 18845749.

5. Schipper J, Helgen KM, Belant JL, González-Maya JF, Eizirik E, Tsuchiya-Jerep M. Small carnivores in the Americas: reflections, future research and conservation priorities. Small Carnivore Conservation. 2009;41:1-2.

6. Crooks KR, Burdett CL, Theobald DM, Rondinini C, Boitani L. Global patterns of fragmentation and connectivity of mammalian carnivore habitat. Philosophical transactions of the Royal Society of London Series B, Biological sciences. 2011;366(1578):2642-51. Epub 2011/08/17. doi: 10.1098/rstb.2011.0120. PubMed PMID: 21844043; PubMed Central PMCID: PMC3140740.

7. González-Maya JF, Cepeda AA, Belant JL, Zárrate-Charry DA, Balaguera-Reina SA, Rodríguez-Bolaños A. Research priorities for the small carnivores of Colombia. Small Carnivore Conservation. 2011;44:7-13.

8. Di Minin E, Slotow R, Hunter LT, Montesino Pouzols F, Toivonen T, Verburg PH, et al. Global priorities for national carnivore conservation under land use change. Sci Rep. 2016;6:23814. Epub 2016/04/02. doi: 10.1038/srep23814. PubMed PMID: 27034197; PubMed Central PMCID: PMCPMC4817124.

9. Belant JL, Schipper J, Conroy J. The conservation status of small carnivores in the Americas. Small Carnivore Conservation. 2009;41:3-8.

10. Ramírez-Chaves HE, González-Maya JF, Schipper J. Small Carnivore Conservation and its contribution to the knowledge of rare small carnivores. Small Carnivore Conservation. 2016;54:1-3.

11. MacDonald DW, Loveridge AJ. The biology and conservation of wild felids. New York, United States: Oxford University Press; 2010.

12. Dickman AJ, Hinks AE, Macdonald EA, Burnham D, Macdonald DW. Priorities for global felid conservation. Conservation Biology. 2015;29(3):854-64. Epub 2015/04/14. doi: 10.1111/cobi.12494. PubMed PMID: 25864434.

13. Ashrafzadeh MR, Khosravi R, Adibi MA, Taktehrani A, Wan HY, Cushman SA. A multi-scale, multi-species approach for assessing effectiveness of habitat and connectivity conservation for endangered felids. Biological Conservation. 2020;245. doi: 10.1016/j.biocon.2020.108523.

14. Payán E, González-Maya JF. Distribución geográfica de la Oncilla (*Leopardus tigrinus*) en Colombia e implicaciones para su conservación. Latin American Journal of Conservation. 2011;2(1):51-9.

15. de Oliveira TG, Tortato MA, Bonjorne de Almeida L, de Campos CB, Beisiegel BM. Avaliação do risco de extinção do Gato-do-mato *Leopardus tigrinus* (Schreber, 1775) no Brasil. Biodiversidade Brasileira. 2013;6(1):56-65.

16. Trigo TC, Schneider A, de Oliveira TG, Lehugeur LM, Silveira L, Freitas TR, et al. Molecular data reveal complex hybridization and a cryptic species of neotropical wild cat. Current biology : CB. 2013;23(24):2528-33. Epub 2013/12/03. doi: 10.1016/j.cub.2013.10.046. PubMed PMID: 24291091.

17. Oliveira do Nascimento F, Feijó A. Taxonomic revision of the Tigrina *Leopardus tigrinus* (Schreber, 1775) species group (Carnivora: Felidae). Papéis Avulsos de Zoologia. 2017;57(19):231-64. doi: 10.11606/0031-1049.2017.57.19.

18. Trindade FJ, Rodrigues MR, Figueiro HV, Li G, Murphy WJ, Eizirik E. Genome-Wide SNPs Clarify a Complex Radiation and Support Recognition of an Additional Cat Species. Mol Biol Evol. 2021;38(11):4987-91. doi: 10.1093/molbev/msab222. PubMed PMID: 34320647; PubMed Central PMCID: PMCPMC8557425.

19. Payan E, de Oliveira T. *Leopardus tigrinus* 2016 [cited 2021 27 March 2021]. The IUCN Red List of Threatened Species]. Available from: https://dx.doi.org/10.2305/IUCN.UK.2016-2.RLTS.T54012637A50653881.en.

20. Oliveira-Santos LGR, Graipel ME, Tortato MA, Zucco CA, Cáceres NC, Goulart FVB. Abundance changes and activity flexibility of the oncilla, *Leopardus tigrinus* (Carnivora: Felidae), appear to reflect avoidance of conflict. Zoologia (Curitiba). 2012;29(2):115-20. doi: 10.1590/S1984-46702012000200003.

21. Trigo TC, Tirelli FP, Machado LF, Peters FB, Indrusiak CB, Mazim FD, et al. Geographic distribution and food habits of *Leopardus tigrinus* and *L. geoffroyi* (Carnivora, Felidae) at their geographic contact zone in southern Brazil. Studies on Neotropical Fauna and Environment. 2013;48(1):56-67. doi: 10.1080/01650521.2013.774789.

22. Pacheco LF, Guerra JF, Deem SL, Frias P. Primer registro de *Leopardus tigrinus* (Shreber, 1775) en Bolivia. Ecología en Bolivia. 2001;36:75-8.

23. Azurduy H. Nota sobre el primer especimen de museo para *Leopardus tigrinus* en Bolivia. Kempffiana. 2005;1(1):47-50.

24. Arias-Alzate A, Sánchez-Londoño JD, Botero-Cañola S, González-Maya JF. Recent confirmed records of the Oncilla (*Leopardus tigrinus*) in the department of Antioquia, Colombia. Mammalogy Notes. 2014;1(2):4-5.

25. Rodgers TW, Kapheim KM. A High-Elevation Record of the Little Spotted Cat (*Leopardus tigrinus oncilla*) from Western Panama. The Southwestern Naturalist. 2017;62(3):225-7. doi: 10.1894/swnat-d-17-00024.1.

26. Suárez-Castro AF, Ramírez-Chaves HE. Los carnívoros terrestres y semiacuáticos continentales de Colombia. Guía de Campo. Bogotá, Colombia: Universidad Nacional de Colombia; 2015. 224 p.

27. Etter A, van Wyngaarden W. Patterns of landscape transformation in Colombia, with emphasis in the Andean region. Ambio. 2000;29(7):432-9.

28. Rosas CA. Temporal changes in the spatial distribution of native forest fragments in the Eastern ridges of Bogota - Implications for Conservation. Bogotá, Colombia: Universidad de los Andes; 2003.

29. Acevedo AA, Lampo M, Cipriani R. The cane or marine toad, *Rhinella marina* (Anura, Bufonidae): two genetically and morphologically distinct species. Zootaxa. 2016;4103(6):574-86. Epub 2016/07/11. doi: 10.11646/zootaxa.4103.6.7. PubMed PMID: 27394759.

30. Etter A, McAlpine C, Wilson K, Phinn S, Possingham H. Regional patterns of agricultural land use and deforestation in Colombia. Agriculture, Ecosystems & Environment. 2006;114(2-4):369–86. doi: 10.1016/j.agee.2005.11.013.

31. Halffter G. La diversidad biológica de Iberoamérica I. Xalapa, Ver., Mexico: Acta Zoológica Mexicana, Instituto de Ecología, A.C.; 1992. 204- p.

32. Corporación Autónoma Regional de Cundinamarca. Plan de Manejo y Conservación de la Oncilla (*Leopardus tigrinus*) para la Jurisdicción de la Corporación Autónoma Regional de Cundinamarca CAR. Bogotá, Colombia: Corporación Autónoma Regional de Cundinamarca CAR, 2019.

33. Muñoz Rojas AD. Evaluación de la conectividad potencial del paisaje para *Leopardus tigrinus* y *Nasuella olivacea* en el bosque Norte de la Reserva Forestal Protectora Bosque Oriental de Bogotá. Bogotá, Colombia: Universidad del Bosque; 2018.

34. Arias-Alzate A, González-Maya JF, Arroyo-Cabrales J, Martínez-Meyer E. Wild felid range shift due to climatic constraints in the Americas: a bottleneck explanation for extinct felids? Journal of Mammalian Evolution. 2016;24(4):427-38. doi: 10.1007/s10914-016-9350-0.

35. Arias-Alzate A, Sánchez-Giraldo C, Botero-Cañola S, Solari S. Aproximación al estado de conservación de las especies de felinos en algunos municipios pertenecientes a las cuencas aportantes del sector eléctrico en jurisdicción de CORANTIOQUIA. Medellín, Colombia: Corporación Autónoma Regional del Centro de Antioquia, 2010.

36. Arias-Alzate A, Sánchez-Giraldo C, Botero-Cañola S, Solari S. Caracterización del estado de los felinos (Carnivora: Felidae) y su interacción con el hombre en el Oriente de Antioquia. Medellín, Colombia: Corporación Autónoma de las Cuencas de los Ríos Negro y Nare, CORNARE, 2012.

37. González-Maya JF, Romero-Rendón JF, Zárrate-Charry DA, Castaño-Uribe C, González M, Víquez-R LR, et al. Evaluación geográfica y prioridades de conservación de hábitat para felinos en el Caribe colombiano. In: Castaño-Uribe C, González-Maya JF, Zárrate-Charry DA, Ange-Jaramillo C, Vela-Vargas IM, editors. Plan de Conservación de Felinos del Caribe Colombiano: Los felinos y su papel en la planificación regional integral basada en especies clave. Santa Marta, Colombia: Fundación Herencia Ambiental Caribe, ProCAT Colombia, The Sierra to Sea Institute; 2013. p. 77-87.

38. Jiménez-Alvarado JS, Moreno-Díaz C, Alfonso AF, Giordano AJ, Vela-Vargas IM, Gómez-Hoyos DA, et al. Ciudades biodiversas: mamíferos medianos de la Reserva Forestal Protectora Bosque Oriental de Bogotá D. C., Colombia. Mammalogy Notes. 2017;4(1):37-41.

39. Gómez-Junco GP, Hurtado-Moreno AP, Moreno-Díaz C, Jiménez-Alvarado JS, González-Maya JF. Caracterización, diagnóstico y desarrollo de un protocolo de prevención, mitigación e intervención frente a eventos de depredación de animales domésticos por fauna silvestre para el territorio CAR. Bogotá, Colombia: Corporación Autónoma Regional de Cundinamarca CAR y Proyecto de Conservación de Aguas y Tierras ProCAT Colombia., 2017.

40. Global Biodiversity Information Facility. GBIF Database of Occurrences. In: Facility GBI, editor. Copenhagen, Denmark2020.

41. Ramírez-Chaves HE, Suárez-Castro AF, Sociedad Colombiana de Mastozoología, Zurc D, Concha Osbahr DC, Trujillo A, et al. Mamíferos de Colombia. Version 1.6. In: Mastozoología SCd, editor. Checklist dataset. 1.6 ed. Bogotá, Colombia: GBIF.org; 2019.

42. Instituto Geográfico Agustín Codazzi (IGAC). Cartografía básica digital integrada. Republica de Colombia. Escala 1:100.000. In: (IGAC) IGAC, editor. Bogotá, Colombia: IGAC; 2016.

43. Zárrate-Charry DA, Massey AL, González-Maya JF, Betts MG. Multi-criteria spatial identification of carnivore conservation areas under data scarcity and conflict: a jaguar case study in Sierra Nevada de Santa Marta, Colombia. Biodiversity and Conservation. 2018;27(13):3373–92. doi: 10.1007/s10531-018-1605-z.

44. Zárrate-Charry D. Use of species distribution information to support landscape management in data-poor countries [PhD]. Corvallos, OR, USA: Oregon State University; 2018.

45. Zárrate Charry DA, González-Maya JF, Arias-Alzate A, Jiménez-Alvarado JS, Reyes Arias JD, Armenteras D, et al. Connectivity conservation at the crossroads: protected areas versus payments for ecosystem services in conserving connectivity for Colombian carnivores. Royal Society Open Science. 2022;9(1):201154. doi: 10.1098/rsos.201154.

46. Hijmans RJ, Cameron SE, Parra JL, Jones PG, Jarvis A. Very high resolution interpolated climate surfaces for global land areas. International Journal of Climatology. 2005;25(15):1965–78. doi: 10.1002/joc.1276.

47. Elith J, Leathwick JR. Species Distribution Models: ecological explanation and prediction across space and time. Annual Review of Ecology, Evolution, and Systematics. 2009;40(1):677-97. doi: 10.1146/annurev.ecolsys.110308.120159.

48. Phillips SJ, Anderson RP, Schapire RE. Maximum entropy modeling of species geographic distributions. Ecological Modelling. 2006;190(3-4):231–59. doi: 10.1016/j.ecolmodel.2005.03.026.

49. Dinerstein E, Olson D, Joshi A, Vynne C, Burgess ND, Wikramanayake E, et al. An ecoregion-based approach to protecting half the terrestrial realm. BioScience. 2017;67(6):534-45. Epub 2017/06/14. doi: 10.1093/biosci/bix014. PubMed PMID: 28608869; PubMed Central PMCID: PMCPMC5451287.

50. Soberon J, Peterson AT. Interpretation of models of fundamental ecological niches and species’ distributional areas. Biodiversity Informatics. 2005;2(0). doi: 10.17161/bi.v2i0.4.

51. Arias-González C, González-Maya JF, González Zamorano P, Ortega Rubio A. Climate refugia for two Colombian endemic tamarin primates are critically under-protected. Mammalian Biology. 2021;101(5):531-43. doi: 10.1007/s42991-021-00151-0.

52. Kalle R, Ramesh T, Qureshi Q, Sankar K. Predicting the distribution pattern of small carnivores in response to environmental factors in the Western Ghats. PLoS One. 2013;8(11):e79295. Epub 2013/11/19. doi: 10.1371/journal.pone.0079295. PubMed PMID: 24244470; PubMed Central PMCID: PMC3828364.

53. Medrano-Vizcaíno P, Gutiérrez-Salazar P. Current and future suitable habitat areas for *Nasuella olivacea* (Gray, 1865) in Colombia and Ecuador and analysis of its distribution across different land uses. Biodiversity Data Journal. 2020;8:e49164. Epub 2020/02/13. doi: 10.3897/BDJ.8.e49164. PubMed PMID: 32047359; PubMed Central PMCID: PMCPMC7000484.

54. Wang C, Fiedler PC. ENSO variability and the eastern tropical Pacific: A review. Progress in Oceanography. 2006;69(2-4):239-66. doi: 10.1016/j.pocean.2006.03.004.

55. Kass JM, Vilela B, Aiello- Lammens ME, Muscarella R, Merow C, Anderson RP, et al. Wallace : A flexible platform for reproducible modeling of species niches and distributions built for community expansion. Methods in Ecology and Evolution. 2018;9(4):1151-6. doi: 10.1111/2041-210x.12945.

56. Phillips SJ, Dudı M, Dudík M. Modeling of species distributions with Maxent : new extensions and a comprehensive evaluation. Ecography. 2008;31:161-75. doi: 10.1111/j.2007.0906-7590.05203.x.

57. Muscarella R, Galante PJ, Soley-Guardia M, Boria RA, Kass JM, Uriarte M, et al. ENMeval: An R package for conducting spatially independent evaluations and

estimating optimal model complexity forMaxentecological niche models. Methods in Ecology and Evolution. 2014;5(11):n/a-n/a. doi: 10.1111/2041-210x.12261.

58. Elith J, Phillips SJ, Hastie T, Dudík M, Chee YE, Yates CJ. A statistical explanation of MaxEnt for ecologists. Diversity and Distributions. 2011;17(1):43–57. doi: 10.1111/j.1472-4642.2010.00725.x.

59. Peterson AT, Soberón J, Pearson RG, Anderson RP, Martínez-Meyer E, Nakamura M, et al. Ecological niches and geographic distributions. Levin SA, Horn HS, editors. Princeton, New Jersey, USA: Princeton University Press; 2011. 328 p.

60. Cobos ME, Peterson AT, Barve N, Osorio-Olvera L. kuenm: an R package for detailed development of ecological niche models using Maxent. PeerJ. 2019;7:e6281. Epub 2019/02/14. doi: 10.7717/peerj.6281. PubMed PMID: 30755826; PubMed Central PMCID: PMCPMC6368831.

61. Radosavljevic A, Anderson RP, Araújo M. Making better Maxent models of species distributions: complexity, overfitting and evaluation. Journal of Biogeography. 2014;41(4):629-43. doi: 10.1111/jbi.12227.

62. IDEAM, IGAC, IAvH, INVEMAR, SINCHI, IIAP. Ecosistemas continentales, costeros y marinos de Colombia. Bogotá, Colombia: Instituto de Hidrología, Meteorología y Estudios Ambientales, Instituto Geográfico Agustín Codazzi, Instituto de Investigación de Recursos Biológicos Alexander von Humboldt, Instituto de Investigaciones Marinas y Costeras "José Benito Vives de Andréis, Instituto Amazónico de Investigaciones Científicas SINCHI, Instituto de Investigaciones Ambientales del Pacífico Jhon von Neumann; 2017.

63. de Oliveira TG, Tortato MA, Silveira L, Kasper CB, Mazim FD, Lucherini M, et al. Ocelot ecology and its effect on the small-felid guild in the lowland neotropics. In:

MacDonald DW, Loveridge A, editors. Biology and Conservation of Wild Felids. Oxford, UK: Oxford University Press; 2010. p. 559-80.

64. Dickson BG, Albano CM, Anantharaman R, Beier P, Fargione J, Graves TA, et al. Circuit-theory applications to connectivity science and conservation. Conservation Biology. 2019;33(2):239–49. Epub 2018/10/13. doi: 10.1111/cobi.13230. PubMed PMID: 30311266; PubMed Central PMCID: PMCPMC6727660.

65. Pelletier D, Clark M, Anderson MG, Rayfield B, Wulder MA, Cardille JA. Applying circuit theory for corridor expansion and management at regional scales: tiling, pinch points, and omnidirectional connectivity. PLoS One. 2014;9(1):e84135. Epub 2014/02/06. doi: 10.1371/journal.pone.0084135. PubMed PMID: 24497918; PubMed Central PMCID: PMC3907452.

66. Correa Ayram CA, Etter A, Díaz-Timoté J, Rodríguez Buriticá S, Ramírez W, Corzo G. Spatiotemporal evaluation of the human footprint in Colombia: four decades of anthropic impact in highly biodiverse ecosystems. Ecological Indicators. 2020;117:106630. doi: 10.1016/j.ecolind.2020.106630.

67. Armenteras D, Gaast F, Villareal H. Andean forest fragmentation and the representativeness of protected natural areas in the eastern Andes, Colombia. Biological Conservation. 2003;113(2):245–56. doi: 10.1016/S0006-3207(02)00359-2.

68. Hand BK, Cushman SA, Landguth EL, Lucotch J. Assessing multi-taxa sensitivity to the human footprint, habitat fragmentation and loss by exploring alternative scenarios of dispersal ability and population size: a simulation approach. Biodiversity and Conservation. 2014;23(11):2761-79. doi: 10.1007/s10531-014-0747-x.

69. Jiménez-Alvarado JS, Hurtado-Moreno AP, Gómez-Hoyos DA, Moreno-Díaz C, González-Maya JF. Monitoreo, evaluación y planificación de conservación de felinos y presas presentes en zonas de explotación de hidrocarburos en el departamento de

Casanare, Colombia: paisajes funcionales de conservación. Bogotá, Colombia: Proyecto de Conservación de Aguas y Tierras – ProCAT Colombia, GEOCOL Consultores & CEPSA Col., 2017.

70. ProCAT Colombia, CORPOGUAVIO. Análisis de conectividad ecológica usando mamíferos como herramientas de gestión entre los Farallones de Medina / Gachalá y el PNN Chingaza. Gachalá, Cundinamarca: Proyecto de Conservación de Aguas y Tierras – ProCAT Colombia, Corporación Autónoma Regional del Guavio – CORPOGUAVIO, 2015.

71. de la Torre JA, Rivero M, Camacho G, Álvarez-Márquez LA. Assessing occupancy and habitat connectivity for Baird's tapir to establish conservation priorities in the Sierra Madre de Chiapas, Mexico. Journal for Nature Conservation. 2018;41:16–25. doi: 10.1016/j.jnc.2017.10.004.

72. McRae BH, Beier P. Circuit theory predicts gene flow in plant and animal populations. Proceedings of the National Academy of Sciences. 2007;104(50):19885–90. Epub 2007/12/07. doi: 10.1073/pnas.0706568104. PubMed PMID: 18056641; PubMed Central PMCID: PMCPMC2148392.

73. McRae BH, Dickson BG, Keitt TH, Shah VB. Using circuit theory to model connectivity in ecology, evolution, and conservation. Ecology. 2008;89(10):2712–24.

74. McRae BH, Hall SA, Beier P, Theobald DM. Where to restore ecological connectivity? Detecting barriers and quantifying restoration benefits. PLoS One. 2012;7(12):e52604. Epub 2013/01/10. doi: 10.1371/journal.pone.0052604. PubMed PMID: 23300719; PubMed Central PMCID: PMCPMC3531461.

75. Environmental Systems Research Institute. ArcGIS 10.5. Redlands, California, USA.: Environmental Systems Research Institute; 2016.

76. Tortato M, de Oliveira T. Ecology of the Oncilla (*Leopardus tigrinus*) at Serra do Tabuleiro State Park, Southern Brazil. CatNews. 2005;42:28-30.

77. Oliveira TGd, Kasper CB, Tortato MA, Marques RV, Mazim FD, Soares JBG, et al. Aspectos da ecologia e conservação de *Leopardus tigrinus* e outros felinos de pequeno-médio porte no Brasil. In: Oliveira TGd, editor. Estudos para o manejo de *Leopardus tigrinus*/Plano de ação para conservação de Leopardus tigrinus no Brasil Relatório final. Atibaia, SP, Brazil: Instituto Pró-Carnívoros/Fundo Nacional do Meio Ambiente; 2008. p. 37-105.

78. Parques Nacionales Naturales de Colombia. Registro Único Nacional de Áreas Protegidas - RUNAP Bogotá, Colombia: Parques Nacionales Naturales de Colombia; 2021 [cited 2021 24 March]. Available from: runap.parquesnacionales.gov.co.

79. United States National Aeronautics and Space Administration (NASA). ASTER global digital elevation model. In: Administration USNAaS, editor. 2009.

80. González-Maya JF, Arias Alzate A. An "update" on the small spotted cats of Colombia (Felidae: *Leopardus* spp.): neglected charismatic species. Wild Felid Monitor. 2015;8(2):19-20.

81. Kitchener AC, Breitenmoser-Würsten C, Eizirik E, Gentry A, Werdelin L, Wilting A, et al. A revised taxonomy of the Felidae. The final report of the Cat Classification Task Force of the IUCN/SSC Cat Specialist Group. CatNews. 2017;11:80.

82. Trigo TC, Freitas TRO, Kunzler G, Cardoso L, Silva JCR, Johnson WE, et al. Inter-species hybridization among Neotropical cats of the genus *Leopardus*, and evidence for an introgressive hybrid zone between *L. geoffroyi* and *L. tigrinus* in southern Brazil. Molecular ecology. 2008;17(19):4317-33. Epub 2008/09/13. doi:

10.1111/j.1365-294X.2008.03919.x. PubMed PMID: 18785898; PubMed Central PMCID: PMCPMC6993176.

83. Gardner AL. Notes on the Little Spotted Cat, *Felis tigrina* oncilla Thomas , in Costa Rica. Journal of Mammalogy. 1971;52(2):464-5.

84. González-Maya JF, Schipper J. A high-elevation report of Oncilla in Mesoamerica. CatNews. 2008;49:33-.

85. Blanco K, Pena R, Hernandez C, Jimenez M, Araya LN, Romero JJ, et al. Serological detection of viral infections in captive wild cats from Costa Rica. Vet Med Int. 2011;2011:879029. Epub 2011/05/07. doi: 10.4061/2011/879029. PubMed PMID: 21547230; PubMed Central PMCID: PMC3087557.

86. Deem SL, Davis R, Pacheco LF. Serologic evidence of nonfatal rabies exposure in a free-ranging Oncilla (*Leopardus tigrinus*) in Cotapata National Park, Bolivia. Journal of Wildlife Diseases. 2004;40(4):811-5. Epub 2005/01/15. doi: 10.7589/0090-3558-40.4.811. PubMed PMID: 15650107.

87. González-Maya JF, Rodríguez-Álvarez C, Arias-Bernal L. Registros de mamíferos medianos y grandes en la Reserva Natural de la Sociedad Civil Jaime Duque, Cundinamarca, Colombia. Revista Mexicana de Mastozoología (Nueva Epoca). 2021;11(1):64-9. doi: 10.22201/ie.20074484e.2021.11.1.328.

88. de Oliveira T, Trigo T, Tortato M, Paviolo A, Bianchi R, Leite-Pitman MRP. *Leopardus guttulus* Gland, Switzerland: International Union for Conservation of Nature; 2016 [cited 2022 06/21/2022].

89. Beger M, McGowan J, Treml EA, Green AL, White AT, Wolff NH, et al. Integrating regional conservation priorities for multiple objectives into national policy. Nat Commun. 2015;6:8208. Epub 2015/09/15. doi: 10.1038/ncomms9208. PubMed PMID: 26364769; PubMed Central PMCID: PMCPMC4579602.

90. Arponen A, Lehtomäki J, Leppänen J, Tomppo E, Moilanen A. Effects of connectivity and spatial resolution of analyses on conservation prioritization across large extents. Conservation Biology. 2012;26(2):294-304. Epub 2012/01/25. doi: 10.1111/j.1523-1739.2011.01814.x. PubMed PMID: 22268786.

91. Clerici N, Salazar C, Pardo-Díaz C, Jiggins CD, Richardson JE, Linares M. Peace in Colombia is a critical moment for Neotropical connectivity and conservation: Save the northern Andes-Amazon biodiversity bridge. Conservation Letters. 2018;12(1). doi: 10.1111/conl.12594.

92. Barriga JC, Diaz-Pulido A, Santamaría M, García H. Catálogo de biodiversidad de las regiones andina, pacífica y piedemonte amazónico. Nivel Local. Volumen 2 Tomo 2. Bogotá, D.C., Colombia: Instituto de Investigación de Recursos Biológicos Alexander von Humboldt – Ecopetrol S.A.; 2016. 423 p.

93. Portocarrero-Aya M, Corzo G, Chaves ME. Catálogo de biodiversidad para las regiones andina, pacífica y piedemonte amazónico. Nivel Regional. Volumen 2 Tomo 1. Bogotá, D.C., Colombia: Instituto de Investigación de Recursos Biológicos Alexander von Humboldt – Ecopetrol S.A.; 2015. 175 p.

94. Romero M, Maldonado- Ocampo JA, Bogotá JD, Usama S, Umaña AM, Murillo J, et al. Informe sobre el estado de la biodiversidad en Colombia 2007-2008 : Piedemonte orinoquense, sabanas y bosques asociados al norte del río Guaviare. Bogotá, Colombia: Instituto de Investigación de Recursos Biológicos Alexander von Humboldt; 2009. 154 p.

95. Vásquez VH, Serrano MA. Las Áreas Naturales Protegidas de Colombia. Bogotá, Colombia: Conservación Internacional-Colombia y Fundacion BioColombia; 2009. 696 p.

96. Gómez-Hoyos DA, Ríos-Franco CA, Marín-Gómez OH, Suarez-Joaqui T, González-Maya JF. Representatividad de mamíferos amenazados en el Sistema Departamental de Áreas Protegidas (SIDAP) del Quindío, Colombia. Mammalogy Notes. 2014;1(2):35-41. doi: 10.47603/manovol1n2.35-41.

97. González-Maya JF, Víquez-R LR, Belant JL, Ceballos G. Effectiveness of Protected Areas for representing species and populations of terrestrial mammals in Costa Rica. Plos One. 2015;10(5):e0124480. Epub 2015/05/15. doi: 10.1371/journal.pone.0124480. PubMed PMID: 25970293; PubMed Central PMCID: PMCPMC4430271.

98. Mayorquín A, Valenzuela S, Rangel - Ch JO. Assessing management effectiveness in Natural Reserves of Civil Society: a methodological proposal. Caldasia. 2010;32(2):381-97.

99. Wolf C, Levi T, Ripple WJ, Zarrate-Charry DA, Betts MG. A forest loss report card for the world's protected areas. Nature Ecology and Evolution. 2021. Epub 2021/02/13. doi: 10.1038/s41559-021-01389-0. PubMed PMID: 33574606.

100. Dudley N, Jonas H, Nelson F, Parrish J, Pyhälä A, Stolton S, et al. The essential role of other effective area-based conservation measures in achieving big bold conservation targets. Global Ecology and Conservation. 2018;15. doi: 10.1016/j.gecco.2018.e00424.

101. Gomes VHF, SD IJ, Raes N, Amaral IL, Salomao RP, de Souza Coelho L, et al. Species Distribution Modelling: Contrasting presence-only models with plot abundance data. Sci Rep. 2018;8(1):1003. Epub 2018/01/19. doi: 10.1038/s41598-017-18927-1. PubMed PMID: 29343741; PubMed Central PMCID: PMCPMC5772443.

102. Guillera-Arroita G, Lahoz-Monfort JJ, Elith J, Gordon A, Kujala H, Lentini PE, et al. Is my species distribution model fit for purpose? Matching data and models to

applications. Global Ecology and Biogeography. 2015;24(3):276-92. doi: 10.1111/geb.12268.

103. Arias-Alzate A, editor. Sistema Local de Áreas Protegidas de Envigado (SILAPE). Envigado, Colombia: Universidad CES, Secretaria de Medio Ambiente y Desarrollo Agropecuario, Corantioquia.; 2016.

104. Andrade GI, Mesa C, Ramírez A, Remolina F. Estructura Ecológica Principal y Áreas Protegidas de Bogotá: oportunidad de integración de políticas para la construcción y el ordenamiento del territorio de la ciudad-región. Foro Nacional Ambiental: Documento de Políticas Públicas. 2008;25:1-12.

105. González-Maya JF, Vela-Vargas IM, Moreno-D. C, Hurtado-Moreno A, Gómez-Junco GP, Aconcha-Abril I, et al. First confirmed record of the Striped Hog-nosed Skunk *Conepatus semistriatus* from peri-urban Bogotá, Colombia. Small Carnivore Conservation. 2017;55:91–6.

Supporting information

S1 Table. Records used for species distribution modeling of *Leopardus tigrinus* in Colombia classified according to filters for type of evidence, source, and geographic precision.

S2 Figure. Map of ecoregions selected for the species distribution modeling of *Leopardus tigrinus* in Colombia. Background shows topography [79]; map created using ArcGIS [75].

S3 Table. Spearman correlation tests for bioclimatic variables used for the distribution model of *Leopardus tigrinus* in Colombia.

S4 Figure. Effect of the predictor variables over *Leopardus tigrinus* distribution model for Colombia; Bio20: Elevation, Bio4: Temperature seasonality, Bio12: Annual precipitation, Bio14: precipitation of the driest month and Bio02: Mean diurnal range.

S5 Table. Models constructed for species distribution modeling and corresponding parameters for *Leopardus tigrinus* in Colombia.

Figure 1

Click here to access/download;Figure;Fig1.tif

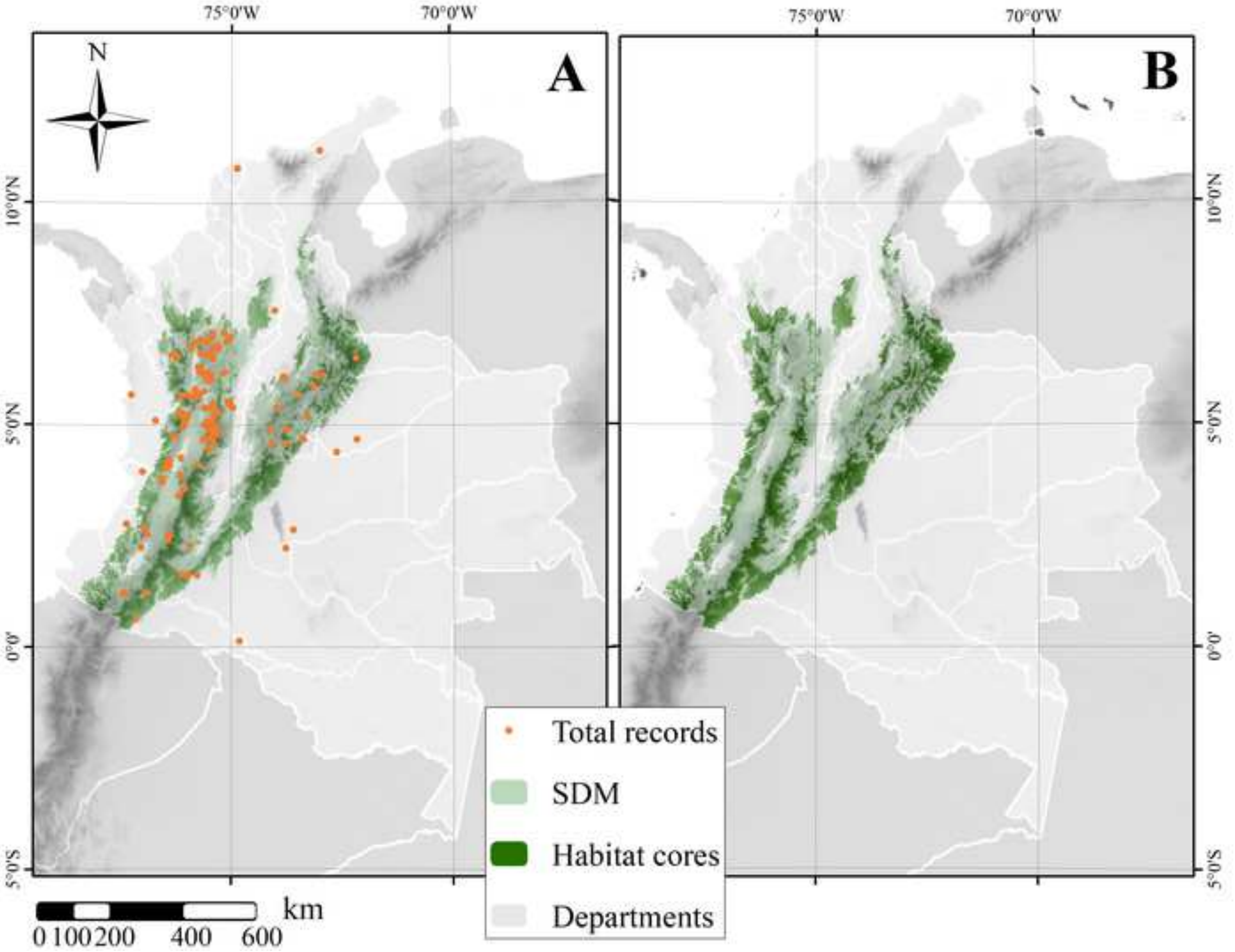

Figure 2

Click here to access/download;Figure;Fig2.tif

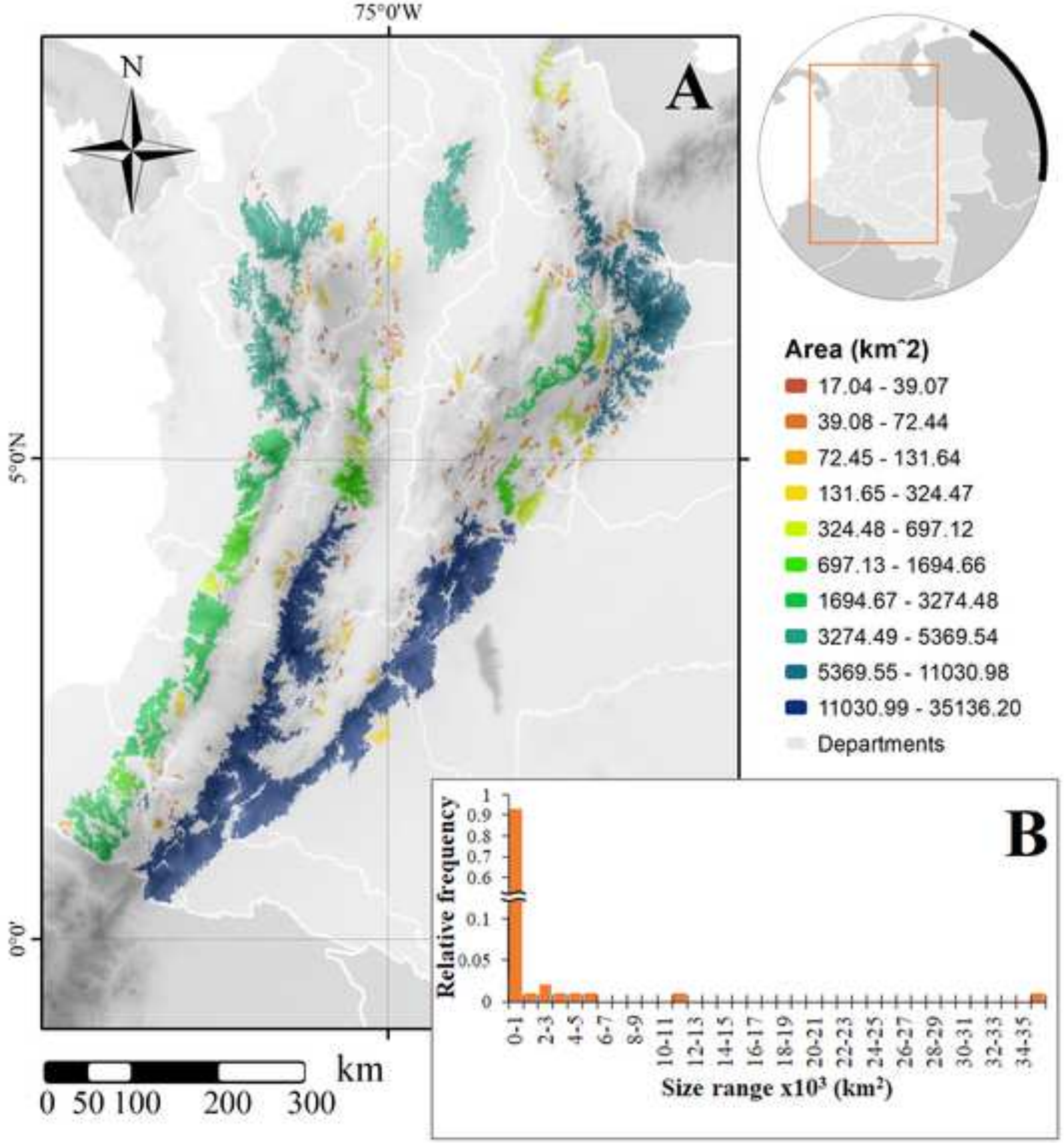

Figure 3

Click here to access/download;Figure;Fig3.tif

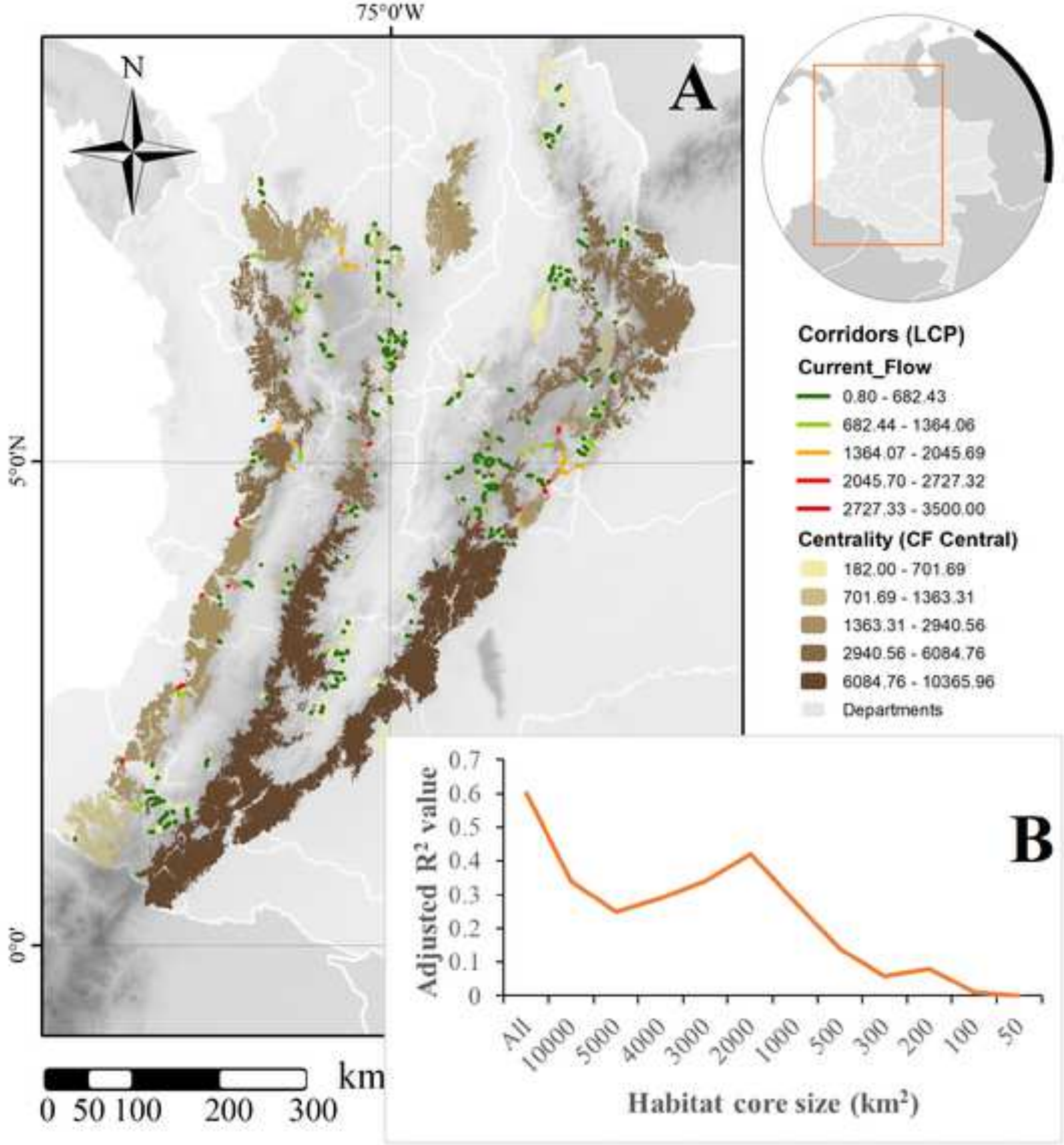

Figure 4

Click here to access/download;Figure;Fig4.tif

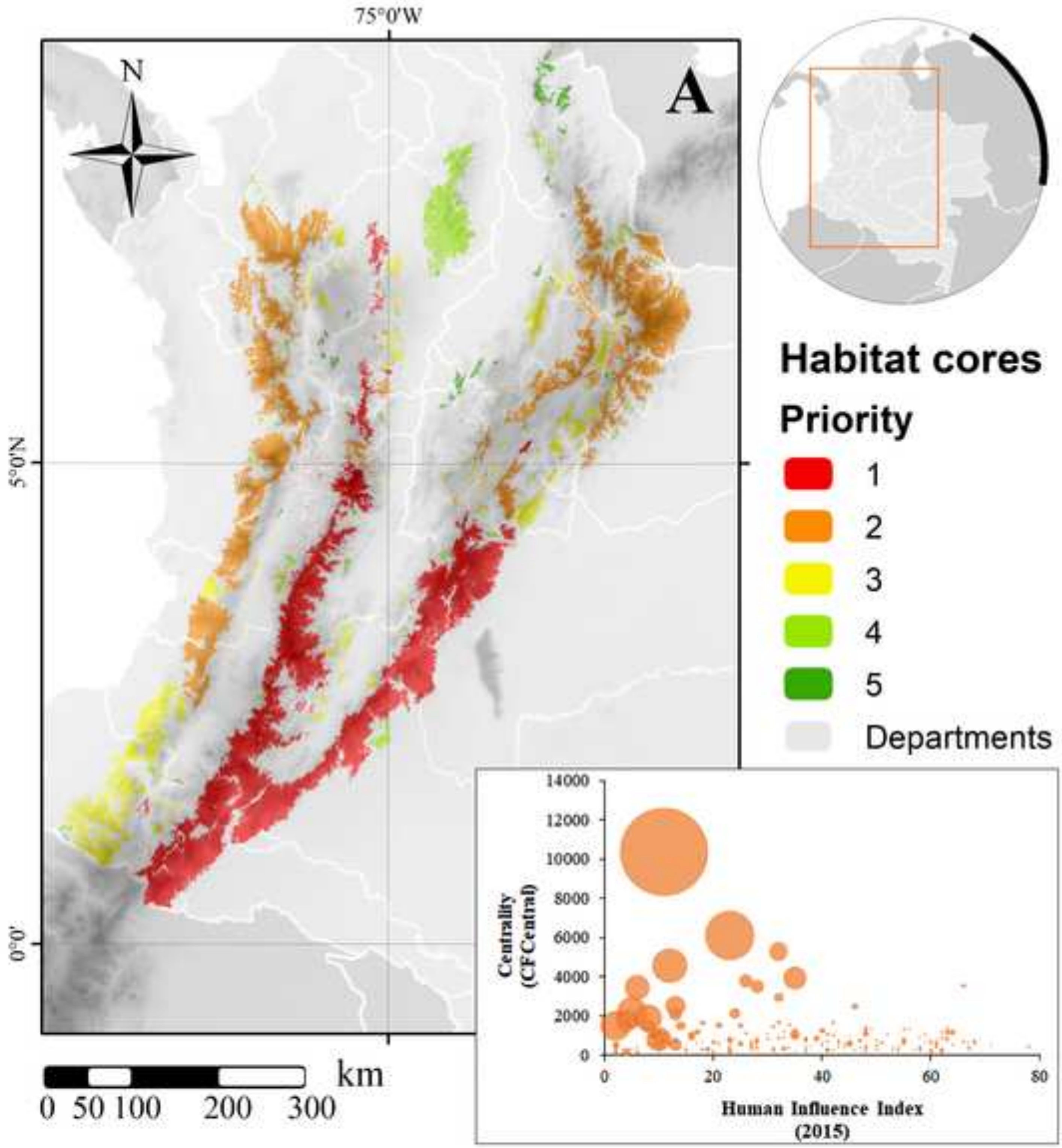

Figure 5 Click here to access/download;Figure;Fig5_pops.tif

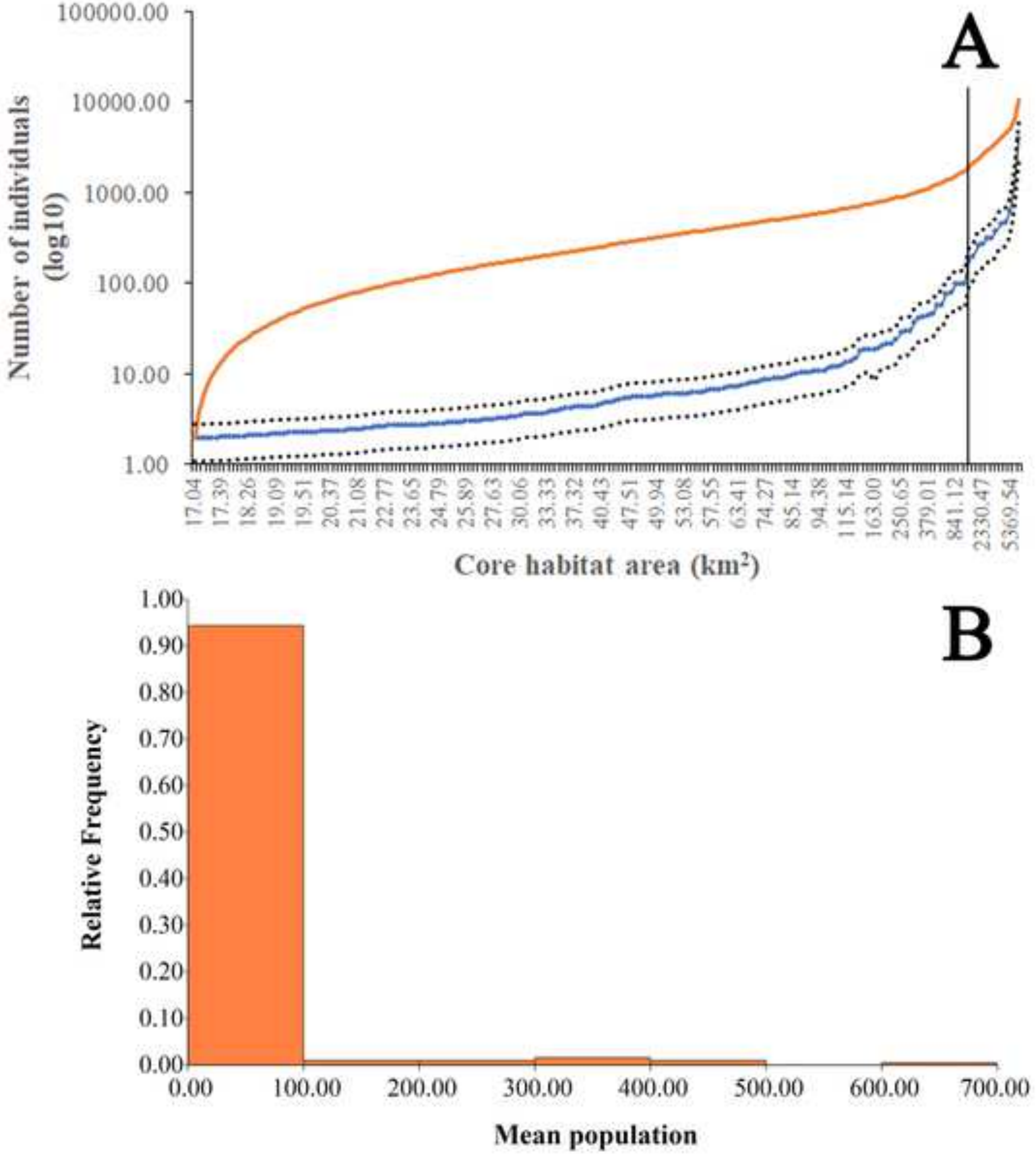

Figure 6

Click here to access/download;Figure;Fig6.tif

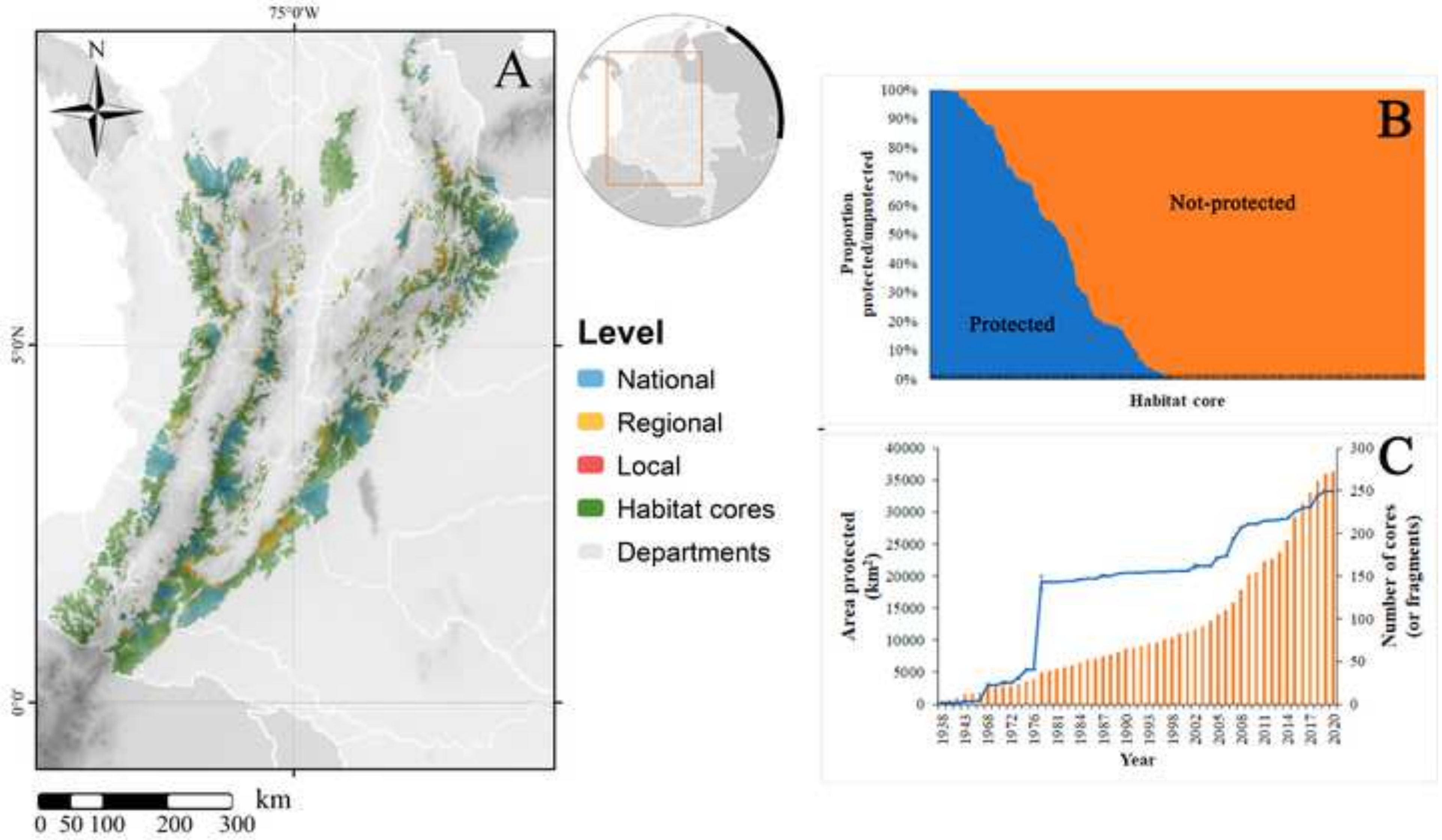

Click here to access/download
Supporting Information
Supp_information.pdf

*Spot*ting what's important: priority areas, connectivity, and conservation of the Northern Tiger Cat (*Leopardus tigrinus*) in Colombia

***Leopardus tigrinus* in Colombia**

José F. González-Maya[1, 2], Diego A. Zárrate-Charry[1, 3], Andrés Arias-Alzate[4], Leonardo Lemus-Mejía[1], Angela P. Hurtado-Moreno[1], Magda Gissella Vargas-Gómez[5], Teresa Andrea Cárdenas[5], Victor Mallarino[6], Jan Schipper[7]

[1] Proyecto de Conservación de Aguas y Tierras – ProCAT Colombia, Carrera 11 # 96-82, Of. 303, Bogotá, Colombia.

[2] Departamento de Ciencias Ambientales, División de Ciencias Biológicas y de la Salud, Universidad Autónoma Metropolitana Unidad Lerma, Lerma de Villada, C. P. 52005, Estado de México, México.

[3] WWF Colombia, Cra. 10a # 69 A – 44, Bogotá, Colombia.

[4] Facultad de Ciencias y Biotecnología, Universidad CES, Calle 10 A # 22-04, Medellín, Colombia.

[5] Grupo Energía Bogotá S.A. E.S.P., Carrera 9 # 73-44, Bogotá D.C., Colombia.

[6] Independent, Bogotá D.C., Colombia.

[7] Arizona Center for Nature Conservation/Phoenix Zoo, Phoenix, Arizona, United States.

* Corresponding author:

E-mail: jfgonzalezmaya@gmail.com.

Abstract

Leopardus tigrinus is among the least known carnivore species in the Andes, including considerable taxonomic uncertainty. ~~Mostly known from Brazil (currently suggested as two distinct species),~~ the Andean and isolated Central American forms remain among the least understood carnivores in the Neotropics. Here we model the distribution, connectivity and overlap with existing conservation areas for the species in Colombia. Using a Species Distribution Modeling approach, we estimated current potential range of the species in Colombia and identified potential habitat blocks remaining in the country. In addition, we designed a connectivity network across the available cores, using a circuit theory approach, to evaluate habitat linkage. Finally, we defined a prioritization scheme for the remaining habitat cores and assessed the level of coverage of protected areas for the country. *L. tigrinus* is potentially present across the three Andean branches of Colombia, with still considerable continuous habitat cores, mostly located on the eastern and central Andean ranges. Most habitat cores are theoretically connected, but nearly 15% are isolated. Priority areas were located across the eastern and central ranges, but with very significant and promising cores in the northern eastern and western ranges. Current level of protection indicates nearly 30% of the range is "protected", but only about 25% is under national strict protected areas. Evolution of this coverage showed some periods of significant increase but interestingly the number of cores grew at a faster rate than overall proportion protected, likely indicating numerous discontinuous fragments, and not contiguous functional landscapes. This represents the most updated assessment of the distribution and conservation status for the species in Colombia, and indicates the numerous conservation opportunities, especially in most populated areas of the country. We found unique business

environmental passive’s opportunities, including compensation and development potential, which are becoming more available in the country.

Key Words: Species distribution modeling, ~~Oncilla,~~ Tigrina, Connectivity, Andes**.**

Introduction

Effective species conservation requires solid scientific information to support decision- and policy-making [1]. Basic information such as distribution, abundance and threats are among the most important aspects needed for effective conservation planning [2, 3], especially when such a large number of species are under threat [4]. Despite their charismatic nature, important role in ecosystem balance and general ecological importance, most carnivores lack even the most basic information [5-7], undermining effective conservation actions, especially given their particular sensitivity to the most pressing threats [8].

Despite the fact that massive efforts have concentrated on a handful of “mega-charismatic” carnivore species worldwide [6], small carnivores have seemingly been overlooked [5, 9]. In fact, some small carnivore species are considered among the least known among mammalian fauna in many countries [5, 10]. Even for wild felids, one of the groups which have received more attention [11], the smaller species are the least understood while at the same time being among the most threatened [11-13]. This is certainly the case for the Northern Tigrina or Northern Tiger Cat, *Leopardus tigrinus*, one of the smallest felids with a wide distribution in the Americas, and perhaps the least understood spotted cat known in the continent [14-16]. Important gaps in information about the species include certainty of its taxonomic status [17-19], as well as even basic knowledge of its distribution, ecology and conservation status [14, 15, 19]; however, significant progress have provided new insights into the group´s taxonomy [16-18]. With a considerably large range, from Costa Rica down to Bolivia and Peru, and East across Venezuela and the Guianas [17], still taxonomic uncertainty does not allow precise distribution assessments, varying according to different authors and taxonomic approaches [16-18]. Mostly unknown, both ecologically and

taxonomically, across its distribution (even with scarce samples), the species has being studied mostly in Brazil [20, 21], where it was even recently split into two distinct species [16, 17]. Information about Northern Tiger Cat for the rest of its range countries is restricted to just occasional records and sporadic observations, but with no systematic data on any other aspects of its ecology and especially conservation status [14, 22-25].

Specifically, in Colombia the species is only known from locality records and observations, with most of the literature focused on distribution and confirmed localities [14, 24, 26], with very few National scale systematic approaches to its distribution and conservation status [14]. Also, in Colombia the species is mostly distributed at higher elevations in the Andes, above 1,500 m asl [14], which is also one of the most transformed and heavily populated regions in the country [27]. Given the considerable pressure over Andean ecosystems in general [28-30], and the specific pressure that such transformation exerts over the species [15], understanding the species distribution and conservation status represents an urgent need as its remaining habitat slowly disappears. Furthermore, given the considerable human development pressure on the remaining habitat for the species, the Northern Tiger Cat has become a predominant species challenging many development and infrastructure projects in the country, necessitating better information that can help informing and plan for its conservation. This could represent a unique conservation opportunity for the species, especially considering the substantial compensation requirements of companies interested in these types of projects. To provide baseline information that can be incorporated in future conservation planning, including compensation and licensing of future development projects, herein we assess the distribution and conservation status of the species in the country. Specifically, i) we assessed the distribution of the species at a national scale, ii) illustrate potential connectivity networks across remnant habitats, iii) selected and

categorized discrete priority remaining habitats, and iv) assessed the conservation status of the priority habitats.

Materials and methods

Study area

Our study focused on the known distribution range of Northern Tiger Cat in Colombia [14], which encompasses the entire terrestrial portion above 2000 m asl of the country. Colombia is located in northwestern South America, bordering with Brazil, Venezuela, Peru, Ecuador and Panama; it is located on the northernmost part of the continent and serves as the only terrestrial limit with Central America. Given the focus of our study, it is worth mentioning that Colombia is also the northernmost part of the Andes where the mountain range splits into three branches (i.e., western, central, and eastern ranges), where they reach elevations over 5000 m. The unique features of the northern Andes represent an interesting case study in terms of evolution and biogeography; the divided mountain range creates unique conditions for a large variety of life-forms with many species ranging on the three branches and some restricted to either of them [31]. Although our study focuses on the higher parts of the Andes, some isolated ranges are also included in the analyses, including the Macarena, Perijá, San Lucas and Sierra Nevada de Santa Marta. These are all independent mountain ranges for the Andes but also reaching elevations where the species is known or thought to occur.

Methods

Species' records

Our species distribution model (SDM) was based on the most updated compilation of confirmed available records for the species, including a thorough

literature review [14, 17, 24, 32-39], and consulting all available national and international databases (i.e., SIB Colombia, GBIF, VertNet [40, 41]) covering a temporal range between 1951 and 2019.

Once a final database was compiled, we discarded all records without specific locality information or coordinates and then used three criteria filtering for data quality control – reducing the available data to only confirmed locations (Table S1). We generated a categorization scheme for each record based on the reliability of ~~the source, type of evidence and geographic precision~~. ~~We classified each record according to the source, the supporting evidence and geographic precision;~~ precision was evaluated by comparing the coincidence between locality details reported in the source and the national cartography of Colombia (Table 1; [42]). ~~Each attribute was classified in three categories (i.e., high, medium, low)~~ that were latter cross referenced for each record (Table 1). We then applied a moderate filter, in which any record with one category ranked as low would be excluded from the final model [43, 44]; we considered this filter as moderate given that we did not use only those categorized as high in the three ~~attributes~~ as for previous efforts [43-45]. Finally, we eliminated duplicates and used a spatial filter which eliminated any redundant records on a 1 km^2 grid [46].

Table 1. Criteria used for classifying ~~three~~ reliability ~~criteria~~ of records for modeling the potential distribution of *L. tigrinus* in Colombia.

**Category**	**Evidence**	**Reliability**
Evidence	Preserved specimens	High
	Machine observation/Photographic record	High
	Material sample	Medium
	Human observation	Low
	No data	Low
Source	Peer reviewed article	High
	Expert validated record	High
	Museum	Medium
	GBIF	Medium
	Technical information	Low
Geographic	Department and Municipality	High
	Department	Medium
	Municipality	Medium
	None	Low

Distribution modelling and potential habitats

For the assessment of the species potential distribution, we generated a SDM based on an ecological niche modeling approach [47] using the maximum entropy algorithm [48]. To define the calibration area of the SDM we obtained an updated polygon layer of the world´s ecoregions [49] as the Mobility area (M) for the species [50] and selected those ecoregions that had at least one record of the species after filtering and we narrowed-down all the inferences to the extent of those ecoregions [51]; all ecoregions selected correspond to those ecoregions associated with the high Andean ecosystems (Figure SI2). We then obtained bioclimatic variables from Worldclim 1.4 [46], including those that represented best the variation for a species such as Northern Tiger Cat, and that have proved useful for modeling carnivore distributions [52, 53]. Specifically, we used seven bioclimatic variables: Mean Annual Temperature (Bio1), Diurnal Mean range (Bio2), Temperature seasonality (Bio4), Annual precipitation (Bio12) and Precipitation seasonality (Bio14), Precipitation of the wettest month (Bio13) and Precipitation of the driest month (Bio14). These last two variables have shown a better representation of the ENSO (El Niño-Southern Oscillation) effects [54]. Finally, we included elevation [42], given its potential influence on species distribution models, especially for the Andean region [51]. We then analyzed potential correlation between these variables across our calibration areas using a Spearman correlation ranks test and the jackknife test based on the maximum entropy model to select those with correlation scores under 70% and the highest permutation values [43].

We used a maximum entropy approach available through the software Wallace 1.0.6.2 [55], which include different R-based packages, including Maxent [56] and ENMeval [57]. Maximum entropy analysis is the most widely used algorithm for quantifying the relationship between the presence of a species and environmental

variables [58, 59]. The records database was divided into calibration and testing dataset following Cobos et al. [60]. For each set of variables, we created candidate models (based on 10 replicates by bootstrapping and selecting the logistic output) using the calibration occurrences, 17 different Regularization Multiplier (RM) values (0.1 – 0.9 at intervals of 0.1, 1 – 6 at intervals of one, and 8, and 10), 29 possible Feature Class (FC) combinations of five feature classes (linear = l, quadratic = q, product = p, threshold = t, and hinge = h), with 10,000 background points. These candidate models were evaluated and selected using the testing dataset, based on statistical significance (Partial ROC, with 500 iterations and 50% data for bootstrapping), omission rate (OR ≤5%), and model complexity with the Akaike Information Criterion corrected for small sample sizes (AICc) [60]. The resulting model was then transformed into binary (i.e., presence-absence), following a conservative threshold, using the 10 percentile training presence logistic threshold value [61], averaged across the 10 replicates.

Selection of available suitable habitats

Once a binary map was obtained from the best model, we evaluated the different types of vegetation cover included in the suitable areas according to available information on species' use [26, 44]. All coverage catalogued as natural by the national cartography dataset [62] and with existing evidence of use by the species from literature and the distribution records, was selected within the binary map [44]. Once all coverages were extracted, we defined an area threshold for selecting those remaining patches that could be used by the species as core habitats, based on a conservative value estimated from the maximum home range reported for the species of 17 km^2. The selection of such threshold aims at prioritizing those core habitat patches that ensure viability of at least one individual considering the inherent uncertainty of the home

range size for the species and the base- data obtained from a different but closely related species (reported values range from 0.9 to 17,1 km^2; [63]).

Connectivity networks definition

Once all available remaining habitats were identified, we designed an ecological connectivity network analysis across the recognized patches based on a circuit theory approach [64, 65]. The ecological connectivity network is based on the resistance that certain landscape features exert over the potential dispersibility of a given species between its core habitats [64, 65]. We consider as core habitats the recognized habitat patches remaining within the suitable areas identified in the species' distribution model. The resistance layer was generated based on the available "human footprint index" developed for Colombia [66]. This human footprint approach includes land uses, rural population density, distance to human settlements and roads, a general fragmentation index, a comparative biomass index compared to the original, and time of intervention [66]. These variables represent multiple levels of human influence over landscapes, including current land use, which can be also used as a proxy to potential barriers (i.e., resistance) to movement across fragmented landscapes [66]. Our assumption is that without knowing the species-specific resistance values that multiple interacting variables can exert over species dispersal, a standardized and weighted human-influence index can validly reflect the resistance of such influence over species movement (especially on highly intervened landscapes such as the Colombian Andean region) [67]. We then used the 2015 Human spatial footprint for Colombia, comparable to our coverage layer for 2016 [62] and used a linear transformation to turn the footprint into a resistance layer by rescaling it to a 1-100 standard scale [68]. The connectivity network was then confined to a 15 km dispersal distance in order to identify corridors that pertain to the species potential movements and thus avoiding overestimations [44]. This

value represents a dispersal distance of an individual with a home range of 4.5 km^2, which represent the median value of all reported home ranges for ~~the species~~ [20, 44, 63]. This method has been previously used for similar purposes in Colombia and the region [33, 43, 69-71]. We then identified the importance of each core habitat by estimating its centrality value (CF_Central value) and the potential current flow for each link within the connectivity network; centrality represents a measure of how important is a linkage for maintaining the overall connectivity network connected, while current flow helps predicting the expected net movement probability of an individual though the linkage [65, 72-74]. We tested the relationship of core habitat size with centrality value using a linear regression and decremental regression analyses (i.e., chained regressions) excluding cores over different size thresholds. We used the adjusted R^2 values to find the threshold where the relationship significantly decreases. Our connectivity network was developed using the Linkage Mapper 10.x toolbox [72, 73] available for ArcGIS [75].

Selection and categorization of priority areas

Once a network of core suitable habitats and a connectivity network was established, we defined discrete units for each core and estimated the following parameters to stablish a priority scheme for Northern Tiger Cat in Colombia. We defined three attributes of the classification criteria that allowed a weighted ranking of all core areas to define a prioritization scheme according to: i) Size, ii) Mean human footprint and iii) Centrality value. Size was estimated as the total area of each discrete polygon identified as core habitat (log-transformed), mean human footprint as the mean value of the 2015 human footprint overlapping the polygon [66] and centrality (log-transformed) as estimated by the circuit analyses [73]. We then summed up the three categories and estimated a final value for each core which was then qualified,

categorized, and standardized generating an ordinal priority from 1 to 5, from the definition of an equal interval of the total range of values, identifying those cores with the best existing conditions (1) to those severely fragmented or affected by human intervention (5).

Conservation measures for Northern Tiger Cat populations in Colombia

Finally, for each core area we estimated several species conservation measures including two parameters and our prioritization scheme. Parameters used are i) number of individuals potentially present on each core and ii) coverage of protected areas. The number of individuals was estimated by extrapolation from known density estimations found elsewhere [15] to the available core habitat areas identified through our models. Density estimations are only available for ~~Brazil~~ [15, 20, 76, 77], so we used all these reports to calculate the minimum number of individuals and the potential variation between estimations; we estimated upper and lower limits and, mean estimation from different sources and distribution of the total number of individuals expected. Specifically, we used complementary estimations, including those considered high, typical and those previously reported for fragmented landscapes according to previous authors [15, 76], and our mean estimate from the existing information. It is necessary to acknowledge the potential bias of our approach given that no specific information exists for the species and for Colombia, and that likely these estimates correspond to *L. guttulus*; nevertheless, given this is the best available information, we believe this approach can be informative until better and more specific information becomes available

For assessing the coverage of protected areas we estimated the overlapping of the remaining core habitats with the national official protected areas layer [78]. First, we

estimated the proportion of core areas covered by all protected areas from the national, regional, and local levels reporting the total level of protection over the entire SDM and for each core. We then estimated the proportion of cores completely or partially protected and according to the protection type. Finally, we assessed how the coverage of protected areas have changed over time to understand how such protection has evolved in terms of total covered area and number of cores fragments included.

All maps were created using ArcGIS [75] and we used a global Digital Elevation Model [79] as background for all map figures.

Results

Distribution of Northern Tiger Cat in Colombia

We obtained a total of 448 records of the species, of which 212 were considered of high quality and credibility (S1 Table). Our sampling area included 6 ecoregions with previously confirmed presence of the Northern Tiger Cat: Cauca Valley montane forests, Cordillera Oriental montane forests, Eastern Cordillera Real montane forests, Magdalena Valley montane forests, Northern Andean paramo and Northwest Andean montane forests (S2 Figure). Derived from the correlation tests, we eliminated from further analyses Annual Mean Temperature, Precipitation of the Wettest Month and Precipitation Seasonality, which were highly correlated to elevation (Spearman= -0.90), Annual Precipitation (Spearman= 0.77) and Precipitation of the Driest Month (Spearman= -0.94), respectively (S3 Table). Among the most important predicting variables, elevation (64.3%) and temperature seasonality (27.9%), accounting as the most important according to permutation importance, but with Annual Precipitation (6.3%), Precipitation of Driest Month (0.94%) and Mean Diurnal Range (0.45%), also influencing its pattern. As expected, elevation had a positive influence on the species´

model (higher probabilities at higher elevations), together with Annual Precipitation, while Temperature Seasonality, Precipitation of the Driest Month and Mean Diurnal Range showed predominantly negative influence (higher ~~variable~~ values, lower probabilities), although with some interesting changes (S4 Figure). The most informative and best selected models were the LQHP (Linear Quadratic Hinge Product) feature class and the 2.5 and 2.0 regularization multipliers models (AICc: 4323.54 and 4323.61, respectively), both models indicate good performance with AUC of 0.8902 and 0.8923, respectively (S5 Table). The resulting 10 percentile training presence logistic threshold binary map, now on SDM, had a total of 228817.43 km^2 of environmentally suitable areas distributed in the three branches of the Andes in Colombia (Fig 1A). However, given the large-scale human-caused natural cover transformation processes across the distribution of the species, our selection of suitable remaining habitats significantly reduced these available areas for the species (Fig 1B).

Figure 1. Distribution of (A) total records obtained for *Leopardus tigrinus* and potential distribution (SDM) in Colombia and (B) remaining habitat cores within the potential distribution (SDM). Background shows topography [79], light gray depicts Colombia and white lines define the political and administrative divisions of Colombia (departments). Map created using ArcGIS [75].

After identifying the available natural coverages within these suitable areas, we found the potentially occupied areas to be only 91,209.85 km^2 (~39.8% of the SDM), distributed in 183 patches, with a mean (±SD) area of 498.41±2783.58 km^2 across the SDM (Fig 2A). This area corresponds to less than 8% of the country area and nearly 40% of the suitable area. We found significant variation among fragments, with many patches covering barely the 17 km^2 threshold (155 (84.7%) under 20 km^2), with 93% under 1,000 km^2 and only one remaining patch covering over 35,000 km^2 (Fig 2B). Most remaining habitat patches were concentrated towards the smaller size, with very few close to the largest one (Fig 2B); although, only 12 patches (all over 100 km^2) cover

together over 76,000 km^2, representing over 83% of the species distribution. The largest remaining habitats are located on the southern central and eastern mountain ranges, including the “macizo colombiano”, where both ranges divide, followed by the northern portion of the eastern range (Fig 2A).

Figure 2. (A) Size of available habitat cores for *Leopardus tigrinus* in Colombia and (B) distribution of fragments according to size classes. Background shows topography [79]; map created using ArcGIS [75].

Potential connectivity network for Leopardus tigrinus in Colombia

Our ecological network analyses selected those areas with the lowest cost for dispersal between habitat cores according to the lowest cost weighted distance between each core. The resulting network, pruned to the maximum 9 km threshold dispersal value, identified approximately 250 links between habitat cores, varying in terms of Euclidean and length distance variation and potential current flow (*sensu* McRae, Dickson (73)). Corridor links mean (±SD) Euclidean and length distances were 2.52±2.45 and 3.31±2.97 km, respectively, while potential current flow was 504.42±650.66 (Fig 3A). Interestingly, 156 of the total 183 had at least one linkage to other cores and the mean (±SD) number of links between cores was 1.61±0.80, with the largest number reaching 5 for a single core.

Centrality values for all cores also varied considerably, with a mean value of 981.53±1128.85 for the entire network, but with an interesting geographical variation (Fig 3A); overall centrality value was closely related with the size of the core (T=16.40, $p<0.001$, R^2Adj=0.60; Fig 3B), indicating both a geographic (location) and spatial influence on the entire network. However, the centrality value variations explained by area (Adjusted R^2) tended to decrease towards the smaller cores, indicating significant importance of the largest ones. Additionally, there is a peak for all cores below 2,000

km^2 (T=11.17, p<0.001, R^2Adj=0.42), which rapidly decrease until only cores below 100 km^2 are included, where the influence is no longer significant (T=1.09, p=0.2782, R^2Adj=0.013; Fig 3B).

Figure 3. Distribution of (A) potential connectivity corridors and centrality values across remaining habitat cores for *L. tigrinus* in Colombia and (B) decremental variation of connectivity importance explained by core area size. Background shows topography [79]; map created using ArcGIS [75].

Priority areas for the conservation of Northern Tiger Cat in Colombia

Our approach for priority areas selection identified those cores that represent the best opportunities for conservation given their available area, their contribution to connectivity and, ideally, where human influence is lower. Mean (±SD) area for all cores was 498.41±2791.22 km^2, but with a concentration towards the smallest patches, as mentioned above. Mean (±SD) value for centrality, importance for the maintenance of the connectivity network, was 981.53±1131.96, where the largest patches were also coincident with those that contribute more significantly to overall connectivity (Fig 3A). In terms of human influence, mean (±SD) index values for all cores was 35.20±19.53, also showing that the largest cores remain with lower human influence (Fig 4B).

After weighting the three criteria, we generated an ordinal priority scheme in five categories (Fig 4A), indicating those currently considered to be strongholds for the species conservation. The main core areas that currently retain ideal conditions for the species, those in Category 1 thus the most preserved, are in the central and eastern ranges of the Andes, concentrated towards the largest, less disturbed, continuous habitat cores in Colombia. However, several opportunities arise for the following two categories, in which important relicts are still present and under appropriate conservation, and those where potentially restoration schemes could significantly

contribute to the maintenance of the species´ populations (Fig 4A). Again, there is a strong relationship between the largest areas with the largest contribution to connectivity and lower levels of human influence (Fig 4B).

Figure 4. (A) Prioritization of remaining habitat cores for *L. tigrinus* in Colombia and (B) relationship between human influence (X-axis), connectivity importance (centrality; Y-axis) and core size (bubble size) of all habitat cores. Background shows topography [79]; map created using ArcGIS [75].

Conservation measures for *Leopardus tigrinus* populations in Colombia

Our estimation of mean number of individuals, based on previous calculations for likely a closely related species, *Leopardus guttulus*, highlight the significant composition of small core habitats, harboring potentially very few individuals. Mean density estimates for the four ranges obtained from previous studies indicated a mean (±SD) density of 0.114±0.051 individuals/km^2. The large variation between previous estimates indicates a potentially large variation in the number of individuals present on each core habitat across the country and thus for the ~~country total,~~ (Table 2). ~~Accordingly, the number of individual Northern Tiger Cat estimated for Colombia, according to the areas with potential occurrence identified by our model, is 10,375±4674 individuals,~~ but with significant variation (Table 2). Only 12 core habitats (above 1000 km^2) contribute over 83% of the total national population (Fig 5A) and almost 95% of the identified potentially cores harbor under 100 individuals (Fig 5B).

Table 2. Population density estimation and potential population size, based on previous estimations, in core habitats within the potential distribution of *L. tigrinus* in Colombia.

Type of estimation	Range	Mean estimated population	±SD	Source
Typical density	0.01-0.05	2736	1824	[15]
High density	0.1-0.25	15962	6841	[15]
	0.15±0.08	13681	7297	[77]
Fragmented landscape*	0.07-0.13	9121	2736	[15]
Mean estimate	0.114±0.051	10375	4674	Our estimation

Figure 5. (A) Mean (blue line), maximum and minimum (dotted lines), number of potential individuals per core habitat organized by increasing size, and cumulative potential population size (orange line) of *L. tigrinus* present at available habitat cores in Colombia and (B) frequency histogram showing the distribution of the number of core habitats according to the potential population size of *L. tigrinus* in Colombia.

Interestingly, in terms of protection level, we found that the species is estimated to be present in at least 415 protected areas from the national to the local level, representing about 33,270.68 km^2 (about 36.47% of the total range) (Fig 6A). The majority, albeit with very low coverage (114.62 km^2), are represented by Civil Society Natural Reserves (Reservas Naturales de la Sociedad Civil), followed by regional protected areas (Regional Integrated Management Districts - Distritos Regionales de Manejo Integrado; Table 3). National Parks (Parques Nacionales Naturales) were the category with the highest proportion of range covered, however, Unique Natural Areas (Área Natural Única) were the ones that covered the least area (2.98 km^2) – which is represented in a single protected area (ANU Los Estoraques; Table 3). It is important to highlight that only 23.71% of the species distribution is covered by dedicated public lands, with mostly strict conservation, while 12.76 % is under private lands where the mixed uses is allowed (Table 3). Furthermore, 25.87% of the range is protected by national level protected areas, 10.47% under regional protected areas and only 0.1% under local protected areas.

Table 3. Total area and number of protected areas according to level and category currently covering the potential distribution range of *L. tigrinus* in Colombia.

Level	Category (Original in Spanish)	Protection level	Lands Regime	Area (km^2)	Number of areas
National	Fauna and Flora Sanctuary (Santuario de Fauna y Flora)	Dedicated	Public	231.17	4
	Flora Sanctuary (Santuario de Flora)	Dedicated	Public	100.86	2
	National Forest Protection Reserve (Reservas Forestales Protectoras Nacionales)	Mixed	Private	1974.69	39
	National Park (Parque Nacional Natural)	Dedicated	Public	21290.29	22
	Natural Unique Area (Area Natural Unica)	Dedicated	Public	2.98	1
Regional	Regional Forest Protection Reserve	Mixed	Private	1126.98	52

	(Reservas Forestales Protectoras Regionales)				
	Regional Integrated Management District (Distritos Regionales de Manejo Integrado)	Mixed	Private	3169.81	53
	Regional Natural Park (Parques Naturales Regionales)	Mixed	Private	5022.62	37
	Soil Conservation District (Distritos de Conservacion de Suelos)	Mixed	Private	236.63	6
Local	Civil Society Natural Reserves (Reserva Natural de la Sociedad Civil)	Mixed	Private	114.63	199
**Total**				**33270.68**	**415**

Of the 183 core habitats, only 92 (50.27%) currently have any level of protection (Fig 6B). Furthermore, mean (±SD) level of protection across cores currently reach 0.51±0.35, while the mean number of protected areas covering a core reached 9.18±28.94. Over time, coverage of remaining core areas has increased significantly since 1938, with notable increases, in terms of number of cores (or fragments of cores) and area, for 1976, 2008 and 2018 (Fig 6C).

Figure 6. (A) Geographic distribution of protected areas currently protecting the potential range of *L. tigrinus* in Colombia, (B) total proportion of protected areas across available habitat cores and (C) temporal evolution of range (line) and habitat cores (bars) protection in Colombia. Background shows topography [79]; map created using ArcGIS [75].

Finally, it is important to highlight that only seven protected areas, primarily from the national level (National Parks), but also one from the regional level (Regional Natural Park), account for almost half (16.76%) of the total range protected. These areas include: El Cocuy (2917.92 km^2), Paramillo (2806.59 km^2), Sumapaz (2144.15 km^2), Cordillera de los Picachos (1918.87 km^2), Farallones de Cali (1658.95 km^2), Nevado del Huila (1586.69 km^2), and Las Hermosas-Gloria Valencia de Castaño (1192.72 km^2) National Parks and Miraflores Picachos Regional Natural Park (1064.28 km^2).

Discussion

Leopardus tigrinus is among the least known carnivore species in Colombia [14, 26, 80] and even across its distribution its taxonomic status is tenuous at best [16-18].

Considering its presumed widespread distribution, and the long-standing uncertainty regarding its taxonomic position, and given that the form present in the Andes and Central America was until recently considered a different species from *L. guttulus* in the south [81, 82], it remains among the most poorly known felids in South America, with knowledge about the species heterogeneously distributed and overall scarce [17-19, 32]. Despite the recent increased attention to the species and considerable advance in its knowledge and study (e.g., [17]), information about its conservation status is still scarce and mostly unavailable for conservation practices [15, 17, 77].

As previously mentioned, information about the Andean and Central American forms is restricted to sporadic distribution records [14, 22-25, 83, 84], with very few research focused on systematic assessments of its distribution [14, 34] and some limited aspects of its ecology [33, 83, 85, 86]. Such information gaps not only limit the overall knowledge of the species, but also the design of appropriate conservation measures, especially considering that the species is categorized as Vulnerable globally on the IUCN Red List of Threatened Species [19]. Here we present an updated, and systematic assessment of the species distribution for Colombia, with significant considerations relevant for Northern Tiger Cat conservation at the national scale. In addition, our methodological approach can be adopted by other countries to evaluate its current conservation status.

Northern Tiger Cat still retains a widespread distribution at high altitude in Colombia, though largely fragmented and located within the most disturbed areas of the country [67]. Although some very considerable remnants remain, mostly on the Eastern and Central ranges, nearly 93% of its current suitable habitat cores are largely fragmented and isolated, with very small potential populations and under protection. Therefore, based on population estimations and conservation assessments for the

species and likely the recently separated *L. guttulus* in Brazil, for instance [15], could indicate, that the Colombian population of *L. tigrinus* is significantly threatened (i.e., from 2,736 individuals using a "typical" density to almost 16,000 individuals on the most optimistic scenario). Especially considering the distribution of the species on the most populated and transformed area of the country [27, 87]. Nevertheless, and considering uncertainty on the actual densities that the species could have in the country, the species could be potentially more threatened than we currently realize, even reaching the critical numbers proposed for the ~~same *L. guttulus* in the south~~ [88].

It is remarkable however that some large continuous habitats remain across the environmentally suitable areas for the species and represent unique opportunities for designing functional landscapes through complementary conservation measures [89], especially those that consider ecological connectivity on large spatial scales [89-91]. Our prioritization approach identified very considerable continuous habitats towards the eastern and central Andean ranges, but with very promising large, forested areas on the northern portion of the eastern and western branches, where numerous conservation opportunities arise. For instance, the Andean-Orinoco piedmont has been widely recognized as a conservation priority, given its unique biota, and thus, it is likely, and desirable, that it will receive substantial conservation efforts [92-94]. Furthermore, the connectivity network proposed in this work would allow to think on functional connected landscapes especially by articulating marginal fragments to the largest core remaining habitats; this is especially necessary to overcome some of the most significant movement barriers, such as Bogota in the Eastern branch, and reconnecting the northernmost portion of the range in Colombia, and South America.

Current coverage of protected areas across the species distribution probably represents the best opportunity for its conservation. However, several considerations

arise from the distribution across multiple levels and categories, and especially given the very low overall protected proportion (i.e., around 50% of all cores and all under 50% protected). For instance, only a very low proportion of the estimated range is under strict, public, protected areas, while a significant proportion is under private lands and with very low or absent active management and protection [95-97]. Furthermore, a significant number of cores are represented on very small areas, mostly private reserves (i.e., Reservas de la Sociedad Civil), that represent a very important conservation strategy [87, 98], which by themselves contribute very little to the overall population conservation goals, but act as important stepping stones between main habitat core; such role heavily depends on restoring connectivity across our proposed corridors´ network. Additionally, we highlight the relevance of seven national protected areas (i.e., El Cocuy, Paramillo, Sumapaz, Cordillera de los Picachos, Farallones de Cali, Nevado del Huila and Las Hermosas - Gloria Valencia de Castaño National Parks) and one recently created regional area (i.e., Miraflores Picachos Regional Natural Park), which represent the most important strongholds for the species. The effective maintenance and management of these areas represent a unique opportunity for the species conservation in the long term [67, 97, 99].

It is important to highlight the exploration of the temporal protected areas evolution covering the current species range. The creation of new protected areas during the 1970s and 2000s, and even with the recent expansion of regional and local level areas, significantly increased the coverage, adding new areas relevant for the species. However, the proportion of area covered is not the most effective metric through time, because the number of fragments seems to growth at a faster rate, revealing a trend that needs further refined analyses. This also to better understand how new conservation figures can better contribute to conserving the species either by adding continuous

habitats or instead, by increasing the number of smaller fragments protected and connected, likely where functionally and genetically isolated population could survive. Nevertheless, at the current pace of creation of new regional and local protected areas it is likely that total coverage for the species will steadily grow benefiting the overall level of protection, but probably requiring complementary conservation measures that warrant functional landscapes; our connectivity network proposal, although can rapidly change according to land cover changes, it probably represent a critical aspect for the functionality and real contribution of this increasing number of protected, yet probably isolated, fragments. Other effective area-based conservation measures (OECMs) articulated with the existing and increasing protected areas can ensure a functional landscapes, both protected and connected, for this species at the long term [100].

Our effort, although with the already known limitations and uncertainty related to SDMs [47, 101, 102], provides a meaningful basic information that could inform and support decision making. The species distribution located in one of the most populated and transformed areas of the country [27, 30, 67] poses an especially complex situation not only for the species conservation, but in general for the Andean biodiversity protection [28, 67], particularly in Colombia [91]. However, recently the species have received special attention given its prominence in the last remaining peri-urban areas on the capital city Bogota [32, 33, 38] and the Aburrá Valley in Antioquia [[103]]. This unique opportunity has drawn attention for the species, especially in the context of cities that demands more resources and territories for supporting an increasing growing population [104, 105]. For instance, the distribution of the species, although based on very limited assessments [32], has being recently used as a conservation spatial determinant for development and infrastructure projects in the region; thus, providing this information represents a unique opportunity to support and safeguard the last

remaining habitats available for the species. Most importantly, it can represent an incredible opportunity for the investment of compensation funds derived from environmental liabilities of development projects; projects that can support and strengthen different conservation strategies within diverse territories by supporting actions within other effective area-based conservation measures.

Here we present not only an updated distribution assessment for one charismatic and poorly known representative species of the Andes, but also several important arguments to support multiple conservation opportunities in Colombia. Although this assessment warrants its validation based on field efforts, it represents a necessary first step for a unique opportunity to appropriately invest the development resources on safeguarding the last remaining Andean habitats in Colombia.

Acknowledgements

This study is part of the ongoing conservation and compensation efforts of Grupo de Energía de Bogotá and ProCAT Colombia for the species in Colombia. Thanks to the staff at GEB and ProCAT for their support throughout the project.

References

1. Schonewald-Cox C, Azari R, Blume S. Scale, variable density, and conservation planning for mammalian carnivores. Conservation Biology. 1991;5(4):491-5. doi: 10.1111/j.1523-1739.1991.tb00356.x.

2. Sanderson EW, Redford KH, Chetkiewicz C-LB, Medellin RA, Rabinowitz AR, Robinson JG, et al. Planning to Save a Species: the Jaguar as a Model. Conservation Biology. 2002;16(1):58-72. doi: 10.1046/j.1523-1739.2002.00352.x.

3. Rondinini C, Di Marco M, Chiozza F, Santulli G, Baisero D, Visconti P, et al. Global habitat suitability models of terrestrial mammals. Philosophical transactions of

the Royal Society of London Series B, Biological sciences. 2011;366(1578):2633-41. Epub 2011/08/17. doi: 10.1098/rstb.2011.0113. PubMed PMID: 21844042; PubMed Central PMCID: PMCPMC3140734.

4. Schipper J, Chanson JS, Chiozza F, Cox NA, Hoffmann M, Katariya V, et al. The status of the world's land and marine mammals: diversity, threat, and knowledge. Science. 2008;322(5899):225-30. Epub 2008/10/11. doi: 10.1126/science.1165115. PubMed PMID: 18845749.

5. Schipper J, Helgen KM, Belant JL, González-Maya JF, Eizirik E, Tsuchiya-Jerep M. Small carnivores in the Americas: reflections, future research and conservation priorities. Small Carnivore Conservation. 2009;41:1-2.

6. Crooks KR, Burdett CL, Theobald DM, Rondinini C, Boitani L. Global patterns of fragmentation and connectivity of mammalian carnivore habitat. Philosophical transactions of the Royal Society of London Series B, Biological sciences. 2011;366(1578):2642-51. Epub 2011/08/17. doi: 10.1098/rstb.2011.0120. PubMed PMID: 21844043; PubMed Central PMCID: PMC3140740.

7. González-Maya JF, Cepeda AA, Belant JL, Zárrate-Charry DA, Balaguera-Reina SA, Rodríguez-Bolaños A. Research priorities for the small carnivores of Colombia. Small Carnivore Conservation. 2011;44:7-13.

8. Di Minin E, Slotow R, Hunter LT, Montesino Pouzols F, Toivonen T, Verburg PH, et al. Global priorities for national carnivore conservation under land use change. Sci Rep. 2016;6:23814. Epub 2016/04/02. doi: 10.1038/srep23814. PubMed PMID: 27034197; PubMed Central PMCID: PMCPMC4817124.

9. Belant JL, Schipper J, Conroy J. The conservation status of small carnivores in the Americas. Small Carnivore Conservation. 2009;41:3-8.

10. Ramírez-Chaves HE, González-Maya JF, Schipper J. Small Carnivore Conservation and its contribution to the knowledge of rare small carnivores. Small Carnivore Conservation. 2016;54:1-3.

11. MacDonald DW, Loveridge AJ. The biology and conservation of wild felids. New York, United States: Oxford University Press; 2010.

12. Dickman AJ, Hinks AE, Macdonald EA, Burnham D, Macdonald DW. Priorities for global felid conservation. Conservation Biology. 2015;29(3):854-64. Epub 2015/04/14. doi: 10.1111/cobi.12494. PubMed PMID: 25864434.

13. Ashrafzadeh MR, Khosravi R, Adibi MA, Taktehrani A, Wan HY, Cushman SA. A multi-scale, multi-species approach for assessing effectiveness of habitat and connectivity conservation for endangered felids. Biological Conservation. 2020;245. doi: 10.1016/j.biocon.2020.108523.

14. Payán E, González-Maya JF. Distribución geográfica de la Oncilla (*Leopardus tigrinus*) en Colombia e implicaciones para su conservación. Latin American Journal of Conservation. 2011;2(1):51-9.

15. de Oliveira TG, Tortato MA, Bonjorne de Almeida L, de Campos CB, Beisiegel BM. Avaliação do risco de extinção do Gato-do-mato *Leopardus tigrinus* (Schreber, 1775) no Brasil. Biodiversidade Brasileira. 2013;6(1):56-65.

16. Trigo TC, Schneider A, de Oliveira TG, Lehugeur LM, Silveira L, Freitas TR, et al. Molecular data reveal complex hybridization and a cryptic species of neotropical wild cat. Current biology : CB. 2013;23(24):2528-33. Epub 2013/12/03. doi: 10.1016/j.cub.2013.10.046. PubMed PMID: 24291091.

17. Oliveira do Nascimento F, Feijó A. Taxonomic revision of the Tigrina *Leopardus tigrinus* (Schreber, 1775) species group (Carnivora: Felidae). Papéis Avulsos de Zoologia. 2017;57(19):231-64. doi: 10.11606/0031-1049.2017.57.19.

18. Trindade FJ, Rodrigues MR, Figueiro HV, Li G, Murphy WJ, Eizirik E. Genome-Wide SNPs Clarify a Complex Radiation and Support Recognition of an Additional Cat Species. Mol Biol Evol. 2021;38(11):4987-91. doi: 10.1093/molbev/msab222. PubMed PMID: 34320647; PubMed Central PMCID: PMCPMC8557425.

19. Payan E, de Oliveira T. *Leopardus tigrinus* 2016 [cited 2021 27 March 2021]. The IUCN Red List of Threatened Species]. Available from: https://dx.doi.org/10.2305/IUCN.UK.2016-2.RLTS.T54012637A50653881.en.

20. Oliveira-Santos LGR, Graipel ME, Tortato MA, Zucco CA, Cáceres NC, Goulart FVB. Abundance changes and activity flexibility of the oncilla, *Leopardus tigrinus* (Carnivora: Felidae), appear to reflect avoidance of conflict. Zoologia (Curitiba). 2012;29(2):115-20. doi: 10.1590/S1984-46702012000200003.

21. Trigo TC, Tirelli FP, Machado LF, Peters FB, Indrusiak CB, Mazim FD, et al. Geographic distribution and food habits of *Leopardus tigrinus* and *L. geoffroyi* (Carnivora, Felidae) at their geographic contact zone in southern Brazil. Studies on Neotropical Fauna and Environment. 2013;48(1):56-67. doi: 10.1080/01650521.2013.774789.

22. Pacheco LF, Guerra JF, Deem SL, Frias P. Primer registro de *Leopardus tigrinus* (Shreber, 1775) en Bolivia. Ecología en Bolivia. 2001;36:75-8.

23. Azurduy H. Nota sobre el primer especimen de museo para *Leopardus tigrinus* en Bolivia. Kempffiana. 2005;1(1):47-50.

24. Arias-Alzate A, Sánchez-Londoño JD, Botero-Cañola S, González-Maya JF. Recent confirmed records of the Oncilla (*Leopardus tigrinus*) in the department of Antioquia, Colombia. Mammalogy Notes. 2014;1(2):4-5.

25. Rodgers TW, Kapheim KM. A High-Elevation Record of the Little Spotted Cat (*Leopardus tigrinus oncilla*) from Western Panama. The Southwestern Naturalist. 2017;62(3):225-7. doi: 10.1894/swnat-d-17-00024.1.

26. Suárez-Castro AF, Ramírez-Chaves HE. Los carnívoros terrestres y semiacuáticos continentales de Colombia. Guía de Campo. Bogotá, Colombia: Universidad Nacional de Colombia; 2015. 224 p.

27. Etter A, van Wyngaarden W. Patterns of landscape transformation in Colombia, with emphasis in the Andean region. Ambio. 2000;29(7):432-9.

28. Rosas CA. Temporal changes in the spatial distribution of native forest fragments in the Eastern ridges of Bogota - Implications for Conservation. Bogotá, Colombia: Universidad de los Andes; 2003.

29. Acevedo AA, Lampo M, Cipriani R. The cane or marine toad, *Rhinella marina* (Anura, Bufonidae): two genetically and morphologically distinct species. Zootaxa. 2016;4103(6):574-86. Epub 2016/07/11. doi: 10.11646/zootaxa.4103.6.7. PubMed PMID: 27394759.

30. Etter A, McAlpine C, Wilson K, Phinn S, Possingham H. Regional patterns of agricultural land use and deforestation in Colombia. Agriculture, Ecosystems & Environment. 2006;114(2-4):369–86. doi: 10.1016/j.agee.2005.11.013.

31. Halffter G. La diversidad biológica de Iberoamérica I. Xalapa, Ver., Mexico: Acta Zoológica Mexicana, Instituto de Ecología, A.C.; 1992. 204- p.

32. Corporación Autónoma Regional de Cundinamarca. Plan de Manejo y Conservación de la Oncilla (*Leopardus tigrinus*) para la Jurisdicción de la Corporación Autónoma Regional de Cundinamarca CAR. Bogotá, Colombia: Corporación Autónoma Regional de Cundinamarca CAR, 2019.

33. Muñoz Rojas AD. Evaluación de la conectividad potencial del paisaje para *Leopardus tigrinus* y *Nasuella olivacea* en el bosque Norte de la Reserva Forestal Protectora Bosque Oriental de Bogotá. Bogotá, Colombia: Universidad del Bosque; 2018.

34. Arias-Alzate A, González-Maya JF, Arroyo-Cabrales J, Martínez-Meyer E. Wild felid range shift due to climatic constraints in the Americas: a bottleneck explanation for extinct felids? Journal of Mammalian Evolution. 2016;24(4):427-38. doi: 10.1007/s10914-016-9350-0.

35. Arias-Alzate A, Sánchez-Giraldo C, Botero-Cañola S, Solari S. Aproximación al estado de conservación de las especies de felinos en algunos municipios pertenecientes a las cuencas aportantes del sector eléctrico en jurisdicción de CORANTIOQUIA. Medellín, Colombia: Corporación Autónoma Regional del Centro de Antioquia, 2010.

36. Arias-Alzate A, Sánchez-Giraldo C, Botero-Cañola S, Solari S. Caracterización del estado de los felinos (Carnivora: Felidae) y su interacción con el hombre en el Oriente de Antioquia. Medellín, Colombia: Corporación Autónoma de las Cuencas de los Ríos Negro y Nare, CORNARE, 2012.

37. González-Maya JF, Romero-Rendón JF, Zárrate-Charry DA, Castaño-Uribe C, González M, Víquez-R LR, et al. Evaluación geográfica y prioridades de conservación de hábitat para felinos en el Caribe colombiano. In: Castaño-Uribe C, González-Maya JF, Zárrate-Charry DA, Ange-Jaramillo C, Vela-Vargas IM, editors. Plan de Conservación de Felinos del Caribe Colombiano: Los felinos y su papel en la planificación regional integral basada en especies clave. Santa Marta, Colombia: Fundación Herencia Ambiental Caribe, ProCAT Colombia, The Sierra to Sea Institute; 2013. p. 77-87.

38. Jiménez-Alvarado JS, Moreno-Díaz C, Alfonso AF, Giordano AJ, Vela-Vargas IM, Gómez-Hoyos DA, et al. Ciudades biodiversas: mamíferos medianos de la Reserva Forestal Protectora Bosque Oriental de Bogotá D. C., Colombia. Mammalogy Notes. 2017;4(1):37-41.

39. Gómez-Junco GP, Hurtado-Moreno AP, Moreno-Díaz C, Jiménez-Alvarado JS, González-Maya JF. Caracterización, diagnóstico y desarrollo de un protocolo de prevención, mitigación e intervención frente a eventos de depredación de animales domésticos por fauna silvestre para el territorio CAR. Bogotá, Colombia: Corporación Autónoma Regional de Cundinamarca CAR y Proyecto de Conservación de Aguas y Tierras ProCAT Colombia., 2017.

40. Global Biodiversity Information Facility. GBIF Database of Occurrences. In: Facility GBI, editor. Copenhagen, Denmark2020.

41. Ramírez-Chaves HE, Suárez-Castro AF, Sociedad Colombiana de Mastozoología, Zurc D, Concha Osbahr DC, Trujillo A, et al. Mamíferos de Colombia. Version 1.6. In: Mastozoología SCd, editor. Checklist dataset. 1.6 ed. Bogotá, Colombia: GBIF.org; 2019.

42. Instituto Geográfico Agustín Codazzi (IGAC). Cartografía básica digital integrada. Republica de Colombia. Escala 1:100.000. In: (IGAC) IGAC, editor. Bogotá, Colombia: IGAC; 2016.

43. Zárrate-Charry DA, Massey AL, González-Maya JF, Betts MG. Multi-criteria spatial identification of carnivore conservation areas under data scarcity and conflict: a jaguar case study in Sierra Nevada de Santa Marta, Colombia. Biodiversity and Conservation. 2018;27(13):3373–92. doi: 10.1007/s10531-018-1605-z.

44. Zárrate-Charry D. Use of species distribution information to support landscape management in data-poor countries [PhD]. Corvallos, OR, USA: Oregon State University; 2018.

45. Zárrate Charry DA, González-Maya JF, Arias-Alzate A, Jiménez-Alvarado JS, Reyes Arias JD, Armenteras D, et al. Connectivity conservation at the crossroads: protected areas versus payments for ecosystem services in conserving connectivity for Colombian carnivores. Royal Society Open Science. 2022;9(1):201154. doi: 10.1098/rsos.201154.

46. Hijmans RJ, Cameron SE, Parra JL, Jones PG, Jarvis A. Very high resolution interpolated climate surfaces for global land areas. International Journal of Climatology. 2005;25(15):1965–78. doi: 10.1002/joc.1276.

47. Elith J, Leathwick JR. Species Distribution Models: ecological explanation and prediction across space and time. Annual Review of Ecology, Evolution, and Systematics. 2009;40(1):677-97. doi: 10.1146/annurev.ecolsys.110308.120159.

48. Phillips SJ, Anderson RP, Schapire RE. Maximum entropy modeling of species geographic distributions. Ecological Modelling. 2006;190(3-4):231–59. doi: 10.1016/j.ecolmodel.2005.03.026.

49. Dinerstein E, Olson D, Joshi A, Vynne C, Burgess ND, Wikramanayake E, et al. An ecoregion-based approach to protecting half the terrestrial realm. BioScience. 2017;67(6):534-45. Epub 2017/06/14. doi: 10.1093/biosci/bix014. PubMed PMID: 28608869; PubMed Central PMCID: PMCPMC5451287.

50. Soberon J, Peterson AT. Interpretation of models of fundamental ecological niches and species’ distributional areas. Biodiversity Informatics. 2005;2(0). doi: 10.17161/bi.v2i0.4.

51. Arias-González C, González-Maya JF, González Zamorano P, Ortega Rubio A. Climate refugia for two Colombian endemic tamarin primates are critically under-protected. Mammalian Biology. 2021;101(5):531-43. doi: 10.1007/s42991-021-00151-0.

52. Kalle R, Ramesh T, Qureshi Q, Sankar K. Predicting the distribution pattern of small carnivores in response to environmental factors in the Western Ghats. PLoS One. 2013;8(11):e79295. Epub 2013/11/19. doi: 10.1371/journal.pone.0079295. PubMed PMID: 24244470; PubMed Central PMCID: PMC3828364.

53. Medrano-Vizcaíno P, Gutiérrez-Salazar P. Current and future suitable habitat areas for *Nasuella olivacea* (Gray, 1865) in Colombia and Ecuador and analysis of its distribution across different land uses. Biodiversity Data Journal. 2020;8:e49164. Epub 2020/02/13. doi: 10.3897/BDJ.8.e49164. PubMed PMID: 32047359; PubMed Central PMCID: PMCPMC7000484.

54. Wang C, Fiedler PC. ENSO variability and the eastern tropical Pacific: A review. Progress in Oceanography. 2006;69(2-4):239-66. doi: 10.1016/j.pocean.2006.03.004.

55. Kass JM, Vilela B, Aiello- Lammens ME, Muscarella R, Merow C, Anderson RP, et al. Wallace : A flexible platform for reproducible modeling of species niches and distributions built for community expansion. Methods in Ecology and Evolution. 2018;9(4):1151-6. doi: 10.1111/2041-210x.12945.

56. Phillips SJ, Dudı M, Dudík M. Modeling of species distributions with Maxent : new extensions and a comprehensive evaluation. Ecography. 2008;31:161-75. doi: 10.1111/j.2007.0906-7590.05203.x.

57. Muscarella R, Galante PJ, Soley-Guardia M, Boria RA, Kass JM, Uriarte M, et al. ENMeval: An R package for conducting spatially independent evaluations and

estimating optimal model complexity forMaxentecological niche models. Methods in Ecology and Evolution. 2014;5(11):n/a-n/a. doi: 10.1111/2041-210x.12261.

58. Elith J, Phillips SJ, Hastie T, Dudík M, Chee YE, Yates CJ. A statistical explanation of MaxEnt for ecologists. Diversity and Distributions. 2011;17(1):43–57. doi: 10.1111/j.1472-4642.2010.00725.x.

59. Peterson AT, Soberón J, Pearson RG, Anderson RP, Martínez-Meyer E, Nakamura M, et al. Ecological niches and geographic distributions. Levin SA, Horn HS, editors. Princeton, New Jersey, USA: Princeton University Press; 2011. 328 p.

60. Cobos ME, Peterson AT, Barve N, Osorio-Olvera L. kuenm: an R package for detailed development of ecological niche models using Maxent. PeerJ. 2019;7:e6281. Epub 2019/02/14. doi: 10.7717/peerj.6281. PubMed PMID: 30755826; PubMed Central PMCID: PMCPMC6368831.

61. Radosavljevic A, Anderson RP, Araújo M. Making better Maxent models of species distributions: complexity, overfitting and evaluation. Journal of Biogeography. 2014;41(4):629-43. doi: 10.1111/jbi.12227.

62. IDEAM, IGAC, IAvH, INVEMAR, SINCHI, IIAP. Ecosistemas continentales, costeros y marinos de Colombia. Bogotá, Colombia: Instituto de Hidrología, Meteorología y Estudios Ambientales, Instituto Geográfico Agustín Codazzi, Instituto de Investigación de Recursos Biológicos Alexander von Humboldt, Instituto de Investigaciones Marinas y Costeras "José Benito Vives de Andréis, Instituto Amazónico de Investigaciones Científicas SINCHI, Instituto de Investigaciones Ambientales del Pacífico Jhon von Neumann; 2017.

63. de Oliveira TG, Tortato MA, Silveira L, Kasper CB, Mazim FD, Lucherini M, et al. Ocelot ecology and its effect on the small-felid guild in the lowland neotropics. In:

MacDonald DW, Loveridge A, editors. Biology and Conservation of Wild Felids. Oxford, UK: Oxford University Press; 2010. p. 559-80.

64. Dickson BG, Albano CM, Anantharaman R, Beier P, Fargione J, Graves TA, et al. Circuit-theory applications to connectivity science and conservation. Conservation Biology. 2019;33(2):239–49. Epub 2018/10/13. doi: 10.1111/cobi.13230. PubMed PMID: 30311266; PubMed Central PMCID: PMCPMC6727660.

65. Pelletier D, Clark M, Anderson MG, Rayfield B, Wulder MA, Cardille JA. Applying circuit theory for corridor expansion and management at regional scales: tiling, pinch points, and omnidirectional connectivity. PLoS One. 2014;9(1):e84135. Epub 2014/02/06. doi: 10.1371/journal.pone.0084135. PubMed PMID: 24497918; PubMed Central PMCID: PMC3907452.

66. Correa Ayram CA, Etter A, Díaz-Timoté J, Rodríguez Buriticá S, Ramírez W, Corzo G. Spatiotemporal evaluation of the human footprint in Colombia: four decades of anthropic impact in highly biodiverse ecosystems. Ecological Indicators. 2020;117:106630. doi: 10.1016/j.ecolind.2020.106630.

67. Armenteras D, Gaast F, Villareal H. Andean forest fragmentation and the representativeness of protected natural areas in the eastern Andes, Colombia. Biological Conservation. 2003;113(2):245–56. doi: 10.1016/S0006-3207(02)00359-2.

68. Hand BK, Cushman SA, Landguth EL, Lucotch J. Assessing multi-taxa sensitivity to the human footprint, habitat fragmentation and loss by exploring alternative scenarios of dispersal ability and population size: a simulation approach. Biodiversity and Conservation. 2014;23(11):2761-79. doi: 10.1007/s10531-014-0747-x.

69. Jiménez-Alvarado JS, Hurtado-Moreno AP, Gómez-Hoyos DA, Moreno-Díaz C, González-Maya JF. Monitoreo, evaluación y planificación de conservación de felinos y presas presentes en zonas de explotación de hidrocarburos en el departamento de

Casanare, Colombia: paisajes funcionales de conservación. Bogotá, Colombia: Proyecto de Conservación de Aguas y Tierras – ProCAT Colombia, GEOCOL Consultores & CEPSA Col., 2017.

70. ProCAT Colombia, CORPOGUAVIO. Análisis de conectividad ecológica usando mamíferos como herramientas de gestión entre los Farallones de Medina / Gachalá y el PNN Chingaza. Gachalá, Cundinamarca: Proyecto de Conservación de Aguas y Tierras – ProCAT Colombia, Corporación Autónoma Regional del Guavio – CORPOGUAVIO, 2015.

71. de la Torre JA, Rivero M, Camacho G, Álvarez-Márquez LA. Assessing occupancy and habitat connectivity for Baird's tapir to establish conservation priorities in the Sierra Madre de Chiapas, Mexico. Journal for Nature Conservation. 2018;41:16–25. doi: 10.1016/j.jnc.2017.10.004.

72. McRae BH, Beier P. Circuit theory predicts gene flow in plant and animal populations. Proceedings of the National Academy of Sciences. 2007;104(50):19885–90. Epub 2007/12/07. doi: 10.1073/pnas.0706568104. PubMed PMID: 18056641; PubMed Central PMCID: PMCPMC2148392.

73. McRae BH, Dickson BG, Keitt TH, Shah VB. Using circuit theory to model connectivity in ecology, evolution, and conservation. Ecology. 2008;89(10):2712–24.

74. McRae BH, Hall SA, Beier P, Theobald DM. Where to restore ecological connectivity? Detecting barriers and quantifying restoration benefits. PLoS One. 2012;7(12):e52604. Epub 2013/01/10. doi: 10.1371/journal.pone.0052604. PubMed PMID: 23300719; PubMed Central PMCID: PMCPMC3531461.

75. Environmental Systems Research Institute. ArcGIS 10.5. Redlands, California, USA.: Environmental Systems Research Institute; 2016.

76. Tortato M, de Oliveira T. Ecology of the Oncilla (*Leopardus tigrinus*) at Serra do Tabuleiro State Park, Southern Brazil. CatNews. 2005;42:28-30.

77. Oliveira TGd, Kasper CB, Tortato MA, Marques RV, Mazim FD, Soares JBG, et al. Aspectos da ecologia e conservação de *Leopardus tigrinus* e outros felinos de pequeno-médio porte no Brasil. In: Oliveira TGd, editor. Estudos para o manejo de *Leopardus tigrinus*/Plano de ação para conservação de Leopardus tigrinus no Brasil Relatório final. Atibaia, SP, Brazil: Instituto Pró-Carnívoros/Fundo Nacional do Meio Ambiente; 2008. p. 37-105.

78. Parques Nacionales Naturales de Colombia. Registro Único Nacional de Áreas Protegidas - RUNAP Bogotá, Colombia: Parques Nacionales Naturales de Colombia; 2021 [cited 2021 24 March]. Available from: runap.parquesnacionales.gov.co.

79. United States National Aeronautics and Space Administration (NASA). ASTER global digital elevation model. In: Administration USNAaS, editor. 2009.

80. González-Maya JF, Arias Alzate A. An "update" on the small spotted cats of Colombia (Felidae: *Leopardus* spp.): neglected charismatic species. Wild Felid Monitor. 2015;8(2):19-20.

81. Kitchener AC, Breitenmoser-Würsten C, Eizirik E, Gentry A, Werdelin L, Wilting A, et al. A revised taxonomy of the Felidae. The final report of the Cat Classification Task Force of the IUCN/SSC Cat Specialist Group. CatNews. 2017;11:80.

82. Trigo TC, Freitas TRO, Kunzler G, Cardoso L, Silva JCR, Johnson WE, et al. Inter-species hybridization among Neotropical cats of the genus *Leopardus*, and evidence for an introgressive hybrid zone between *L. geoffroyi* and *L. tigrinus* in southern Brazil. Molecular ecology. 2008;17(19):4317-33. Epub 2008/09/13. doi:

10.1111/j.1365-294X.2008.03919.x. PubMed PMID: 18785898; PubMed Central PMCID: PMCPMC6993176.

83. Gardner AL. Notes on the Little Spotted Cat, *Felis tigrina* oncilla Thomas , in Costa Rica. Journal of Mammalogy. 1971;52(2):464-5.

84. González-Maya JF, Schipper J. A high-elevation report of Oncilla in Mesoamerica. CatNews. 2008;49:33-.

85. Blanco K, Pena R, Hernandez C, Jimenez M, Araya LN, Romero JJ, et al. Serological detection of viral infections in captive wild cats from Costa Rica. Vet Med Int. 2011;2011:879029. Epub 2011/05/07. doi: 10.4061/2011/879029. PubMed PMID: 21547230; PubMed Central PMCID: PMC3087557.

86. Deem SL, Davis R, Pacheco LF. Serologic evidence of nonfatal rabies exposure in a free-ranging Oncilla (*Leopardus tigrinus*) in Cotapata National Park, Bolivia. Journal of Wildlife Diseases. 2004;40(4):811-5. Epub 2005/01/15. doi: 10.7589/0090-3558-40.4.811. PubMed PMID: 15650107.

87. González-Maya JF, Rodríguez-Álvarez C, Arias-Bernal L. Registros de mamíferos medianos y grandes en la Reserva Natural de la Sociedad Civil Jaime Duque, Cundinamarca, Colombia. Revista Mexicana de Mastozoología (Nueva Epoca). 2021;11(1):64-9. doi: 10.22201/ie.20074484e.2021.11.1.328.

88. de Oliveira T, Trigo T, Tortato M, Paviolo A, Bianchi R, Leite-Pitman MRP. *Leopardus guttulus* Gland, Switzerland: International Union for Conservation of Nature; 2016 [cited 2022 06/21/2022].

89. Beger M, McGowan J, Treml EA, Green AL, White AT, Wolff NH, et al. Integrating regional conservation priorities for multiple objectives into national policy. Nat Commun. 2015;6:8208. Epub 2015/09/15. doi: 10.1038/ncomms9208. PubMed PMID: 26364769; PubMed Central PMCID: PMCPMC4579602.

90. Arponen A, Lehtomäki J, Leppänen J, Tomppo E, Moilanen A. Effects of connectivity and spatial resolution of analyses on conservation prioritization across large extents. Conservation Biology. 2012;26(2):294-304. Epub 2012/01/25. doi: 10.1111/j.1523-1739.2011.01814.x. PubMed PMID: 22268786.

91. Clerici N, Salazar C, Pardo-Díaz C, Jiggins CD, Richardson JE, Linares M. Peace in Colombia is a critical moment for Neotropical connectivity and conservation: Save the northern Andes-Amazon biodiversity bridge. Conservation Letters. 2018;12(1). doi: 10.1111/conl.12594.

92. Barriga JC, Diaz-Pulido A, Santamaría M, García H. Catálogo de biodiversidad de las regiones andina, pacífica y piedemonte amazónico. Nivel Local. Volumen 2 Tomo 2. Bogotá, D.C., Colombia: Instituto de Investigación de Recursos Biológicos Alexander von Humboldt – Ecopetrol S.A.; 2016. 423 p.

93. Portocarrero-Aya M, Corzo G, Chaves ME. Catálogo de biodiversidad para las regiones andina, pacífica y piedemonte amazónico. Nivel Regional. Volumen 2 Tomo 1. Bogotá, D.C., Colombia: Instituto de Investigación de Recursos Biológicos Alexander von Humboldt – Ecopetrol S.A.; 2015. 175 p.

94. Romero M, Maldonado- Ocampo JA, Bogotá JD, Usama S, Umaña AM, Murillo J, et al. Informe sobre el estado de la biodiversidad en Colombia 2007-2008 : Piedemonte orinoquense, sabanas y bosques asociados al norte del río Guaviare. Bogotá, Colombia: Instituto de Investigación de Recursos Biológicos Alexander von Humboldt; 2009. 154 p.

95. Vásquez VH, Serrano MA. Las Áreas Naturales Protegidas de Colombia. Bogotá, Colombia: Conservación Internacional-Colombia y Fundacion BioColombia; 2009. 696 p.

96. Gómez-Hoyos DA, Ríos-Franco CA, Marín-Gómez OH, Suarez-Joaqui T, González-Maya JF. Representatividad de mamíferos amenazados en el Sistema Departamental de Áreas Protegidas (SIDAP) del Quindío, Colombia. Mammalogy Notes. 2014;1(2):35-41. doi: 10.47603/manovol1n2.35-41.

97. González-Maya JF, Víquez-R LR, Belant JL, Ceballos G. Effectiveness of Protected Areas for representing species and populations of terrestrial mammals in Costa Rica. Plos One. 2015;10(5):e0124480. Epub 2015/05/15. doi: 10.1371/journal.pone.0124480. PubMed PMID: 25970293; PubMed Central PMCID: PMCPMC4430271.

98. Mayorquín A, Valenzuela S, Rangel - Ch JO. Assessing management effectiveness in Natural Reserves of Civil Society: a methodological proposal. Caldasia. 2010;32(2):381-97.

99. Wolf C, Levi T, Ripple WJ, Zarrate-Charry DA, Betts MG. A forest loss report card for the world's protected areas. Nature Ecology and Evolution. 2021. Epub 2021/02/13. doi: 10.1038/s41559-021-01389-0. PubMed PMID: 33574606.

100. Dudley N, Jonas H, Nelson F, Parrish J, Pyhälä A, Stolton S, et al. The essential role of other effective area-based conservation measures in achieving big bold conservation targets. Global Ecology and Conservation. 2018;15. doi: 10.1016/j.gecco.2018.e00424.

101. Gomes VHF, SD IJ, Raes N, Amaral IL, Salomao RP, de Souza Coelho L, et al. Species Distribution Modelling: Contrasting presence-only models with plot abundance data. Sci Rep. 2018;8(1):1003. Epub 2018/01/19. doi: 10.1038/s41598-017-18927-1. PubMed PMID: 29343741; PubMed Central PMCID: PMCPMC5772443.

102. Guillera-Arroita G, Lahoz-Monfort JJ, Elith J, Gordon A, Kujala H, Lentini PE, et al. Is my species distribution model fit for purpose? Matching data and models to

applications. Global Ecology and Biogeography. 2015;24(3):276-92. doi: 10.1111/geb.12268.

103. Arias-Alzate A, editor. Sistema Local de Áreas Protegidas de Envigado (SILAPE). Envigado, Colombia: Universidad CES, Secretaria de Medio Ambiente y Desarrollo Agropecuario, Corantioquia.; 2016.

104. Andrade GI, Mesa C, Ramírez A, Remolina F. Estructura Ecológica Principal y Áreas Protegidas de Bogotá: oportunidad de integración de políticas para la construcción y el ordenamiento del territorio de la ciudad-región. Foro Nacional Ambiental: Documento de Políticas Públicas. 2008;25:1-12.

105. González-Maya JF, Vela-Vargas IM, Moreno-D. C, Hurtado-Moreno A, Gómez-Junco GP, Aconcha-Abril I, et al. First confirmed record of the Striped Hog-nosed Skunk *Conepatus semistriatus* from peri-urban Bogotá, Colombia. Small Carnivore Conservation. 2017;55:91–6.

Supporting information

S1 Table. Records used for species distribution modeling of *Leopardus tigrinus* in Colombia classified according to filters for type of evidence, source, and geographic precision.

S2 Figure. Map of ecoregions selected for the species distribution modeling of *Leopardus tigrinus* in Colombia. Background shows topography [79]; map created using ArcGIS [75].

S3 Table. Spearman correlation tests for bioclimatic variables used for the distribution model of *Leopardus tigrinus* in Colombia.

S4 Figure. Effect of the predictor variables over *Leopardus tigrinus* distribution model for Colombia; Bio20: Elevation, Bio4: Temperature seasonality, Bio12: Annual precipitation, Bio14: precipitation of the driest month and Bio02: Mean diurnal range.

S5 Table. Models constructed for species distribution modeling and corresponding parameters for *Leopardus tigrinus* in Colombia.
