# Supplementary material for: Spotting what’s important: Priority areas, connectivity, and conservation of the Northern Tiger Cat (Leopardus tigrinus) in Colombia
Source: PLoS One. 2022 Sep 13;17(9):e0273750. doi: 10.1371/journal.pone.0273750 (PMC9469974; doi:10.1371/journal.pone.0273750)
Supplement: Attachment — Submitted filename: Response Letter.doc [file pone.0273750.s007.doc]

**Responses to reviewers**

# Reviewer #1

Reviewer #1: Dear Editor, I believe that the authors have made a good review, appropriately answering all my comments and making the necessary changes and corrections. In my opinion, the manuscript is now suitable to be accepted for publication in PlosOne. I am satisfied with the review performed, having only pointed out a few minor text corrections in the article submission pdf itself. My comments are on the version with track changes. I do not need to see a revised version. Best wishes.

R/Thanks for the comments, we really appreciate the feedback and constructive criticism!

Eliminate: Mostly known from Brazil (currently suggested as two distinct species)

R/Changed.

Since you are talking now only about the northern tiger cat, this part of the introduction makes no more sense. The cited works (20 and 21) were done in regions of L. guttulus occurrence and if we follow Nascimento & Feijó's classification, we will have L. tigrinus with a restricted distribution in Brazil and as far as I know, we have no studies with these populations. I think it is better to avoid comparative texts with Brazilian populations mainly due to taxonomic uncertainty of tigrinus/emiliae in Brazil.

Also, Nascimento & Feijó split it into 3 species in Brazil: L. tigrinus, L. emiliae and L. guttulus

R/Agreed. We eliminated the phrase and changed for readability.

three criteria: source, evidence and geographic precision.

R/Changed.

Text suggestion:

Each criterion showed different attributes with an assigned reliability category (high, medium, low) that were latter....

R/Changed.

criteria as in previous studies

R/Corrected.

Erase Three and Criteria

R/Corrected.

You need to standardize the terms used here, from what I understand from the text, "Category" title of the first column would be "Criteria", "Evidence" in the second column would be Attributes

R/Corrected.

Geographic precision

R/Corrected

Brazilian tiger cats

R/Corrected.

(Southern Tiger Cat)

R/Added.

whole country

R/Corrected.

According to the areas with potential occurrence identified by our model, the number of Northern Tiger Cat individuals estimated for Colombia is...

R/Changed.

Southern Tiger Cat

R/Changed.

# Reviewer #2

Dear Editor,

I believe the quality of the manuscript has greatly increased, but there are some points that still require attention:

R/ Thank you so much for all the revisions and comments!

Lines 75-76 – I would add “central and northern Brazil” as according to the IUCN, L. tigrinus’ range encompasses Brazil; and is not clear if the species from Costa Rica (identified by Trindade et al. 2021) is the same or different one from the one found in Colombia.

R/We changed to reflect that the Costa Rica form could change, but given the evidence from recent papers and the comments from the other reviewer, we didn’t include Brazil.

Line 221 – Add “potential” dispersal distance.

R/Corrected.

Line 228 – change “though” to “through”.

R/Corrected.

Line 290-292 – change “Among the most important predicting variables, elevation (64.3%) and temperature seasonality (27.9%), accounting as the most important according to permutation importance” to “Among the selected variables, elevation (64.3%) and temperature seasonality (27.9%) accounted as the most important according to permutation importance”.

R/Corrected.

Figure 1 – Figure 1A is a bit confusing. According to the legend, only the lighter green is SDM, so what is the darker green in figure A? It seems the darker green is the habitat cores, but the figure caption states that figure A only demonstrates the species records and the SDM. If it is the habitat cores, I do not see a reason for the existence of figure B. Please clarify.

R/Absolutely right! We changed and just left one map.

Line 323 – I believe the 83% is related to the habitat cores and not the SDM, as previously it is stated that the potentially occupied areas represent ~40% of the SDM. Therefore, it would be good to clarify it, maybe changing “83% of the species distribution” to “83% of the identified core areas”, as species distribution may also be related to the SDM.

R/Corrected.

Line 335 – Here says that the maximum dispersal distance used was 9km, but on the methods (line 219) says it was 15km. Please clarify.

R/Corrected.

Figure 6 – the colors of figure 6A are hard to distinguish. It may be due to lower resolution of the figure attached in the file. Perhaps it would be interesting to change the colors or try to use polygons or lines overlap to make it easier for the reader.

R/We changed the colors; we do think is a matter of resolution. Shadows, given the complexity of shapes, just looks more confusing and messier.

Line 450 – change “a different” to “the same” or “was until recently considered” to “was recently recognized as different species”. The Andean and Costa Rican tigrina were until recently considered the same species from L. guttulus. They were separated in two species in 2013 (Trigo et al.). In addition, Trindade et al. 2021 showed that the Costa Rican tigrina is also a separate species from the L. tigrinus that occurs in the north and northeastern Brazil.

R/Changed to reflect both aspects.

Line 480 – remove “same”.

R/Corrected.

Line 495 – I suggest removing “and South America” or rephrasing this sentence.

R/Removed.

Line 526 – add “the”: is likely that “the” total coverage for the species…

R/Corrected.

Line 529 – remove “it”.

R/Removed.
